# Assessing the Impact of Geographical Distribution and Genetic Diversity on Metabolic Profiles of a Medicinal Plant, *Embelia ribes* Burm. f.

**DOI:** 10.3390/plants11212861

**Published:** 2022-10-27

**Authors:** Shubhangi Raskar, Vishwabandhu Purkar, Milind Sardesai, Sirsha Mitra

**Affiliations:** Department of Botany, Savitribai Phule Pune University, Pune 411007, India

**Keywords:** ethnobotany, herbal medicine, genetic variation, geographic distribution, liquid chromatography-mass spectrometry, metabolomics

## Abstract

The extensive use of *Embelia ribes* Burm. f. (Embelia) in tribal medicine proclaimed global attention as a promising candidate in complementary and alternative medicine. The knowledge of chemical blends is a prerequisite for the selection of raw materials for herbal medicine formulations; however, the influence of geographical distance and genetic diversity on the metabolome of Embelia fruits is unknown. Therefore, we collected Embelia fruits from four locations across the Western Ghats of India and analyzed the metabolic profile and genotypic diversity of Embelia fruits by liquid chromatography-tandem mass spectrometry (LC-MS/MS) and inter simple sequence repeats (ISSR), respectively. LC-MS/MS analysis yielded 583 compounds; however, the trimmed data resulted in 149 compounds. Further, MS/MS analysis identified 36 compounds, among which we reported 30 compounds for the first time from Embelia. These compounds belong to 11 compound classes that suggest location-specific chemical blends of Embelia fruits. Multivariate analysis showed 94% compound diversity across the accessions. ISSR analysis suggests 95% polymorphism across the accessions. A significant positive correlation (80%) between metabolomics and genotypic data matrices validates the genotype’s influence in tuning Embelia’s metabolic profiles. We conclude that the chemical profiles of Embelia are location-specific, which can be explored for the selection of herbal trade sustainably.

## 1. Introduction

*Embelia ribes* Burm. f. (Embelia) [1] is a liana that belongs to the family Primulaceae. Since ancient times, the use of Embelia fruits has been widespread in the form of the drug *Vidanga*. Embelia’s distribution in India ranges from the outer Himalayas to the Western Ghats, at an elevation of up to 1500 m [2] in fragmented populations. The sparse distribution of Embelia in the evergreen to moist deciduous forests of Maharashtra, Karnataka, and Kerala in the Western Ghats and Tamil Nadu in the Eastern Ghats, is evident. The different plant parts of Embelia are used in herbal formulations, as it is rich in various medicinally important molecules, namely embelin and its derivatives, embeliol, 5-O-Methylembelin, and vilangin that have high commercial value. Hence, the pharmacology of Embelia has attracted global attention as a promising candidate in various traditional, complementary, and alternative systems of medicine. In traditional and tribal medicine, the fruits, stems, roots, and leaves of Embelia are used as anthelmintic, antipyretic, and antimicrobial agents. In addition, they are also used to treat diarrhea, kidney stones, snake bites, and bronchitis. However, the fruits of Embelia are more common in traditional medicine and herbal formulations than their leaves and roots. Interestingly, the use of the same plant parts varies from region to region. For example, fruit decoction of Embelia is given for intestinal worms in Kerala [3], antimicrobial medication in Karnataka [4], diarrhea in Arunachal Pradesh, and influenza and snakebite in the Khandesh region of Maharashtra [5]. However, as the climatic condition of the Khandesh region is not suitable for Embelia growth, its use in this region questions the authentication of the used fruits.

The geographical origin and climatic conditions are the notable factors that affect the metabolome of a plant. Plants are adapted to the different geographical, climatic, and edaphic conditions by genotypic and phenotypic alterations. The genotypic alteration also influences the production and accumulation of secondary metabolites in plants [6,7]. Though the specialized metabolite profile is unique to individuals within a species or a close taxonomic group, it may alter if its biosynthetic pathways are influenced by environmental conditions such as climate, soil, pathogens infection, and pest infestation. Therefore, regional variation in the use of the same plant parts can be due to the presence of different blends or proportions of active compounds in them, which in turn links the geography and climate of the habitat of Embelia. In 2011, Saurabh demonstrated that geographical topography and climatic conditions profoundly affect the levels of polyphenols in the bark of *Bridelia retusa* [8]; similarly, the effect of geographical and climatic conditions on the camptothecin content in *Nothapodytes nimmoniana* is also demonstrated [9]. Recently, a comparative study of the concentrations of embelin, Embelia’s principle component in its fruit from different geographical regions, underlined that the geographic location of a plant could govern the change in the concentration of its principal component [10]. Previously, a comparative analysis of fatty acid composition in the seeds of *E. schimperi* [11] and fatty acids and esters from *E. basal* have been attempted [12]; however, the non-targeted metabolomics of Embelia fruits from different geographical regions are yet to be revealed.

Metabolomics has been used for the identification of marker compounds to detect physiological changes, genotype differences, geographic origin, and quality control [13,14]. In a study with the fruit of *Butia* sp., rutin, epicatechin, isorhamnetin, and stilbene were identified as the significant markers in discriminating the geographic origins and species [15]. Moreover, with the globalization of traditional medicine systems such as Ayurvedic medicine and traditional Chinese medicine, many Asian medicinal plant species are being introduced to cultivation outside of their geographical origins, particularly in the EU and US [16]. As these ecosystems are significantly different from their native origin, these species suffer from measurable differences in chemical composition. In general, studies focus on a single compound or a class of compounds to evaluate the effect of climatic or geographical conditions [17]; however, a plant’s whole metabolome is rarely considered for intensive analysis in response to changes in its geographical conditions. To improve global health through traditional and complementary medicine, the World Health Organization (WHO) emphasizes the rational use of raw materials based on evidence and strategic research in traditional and complementary medicine. Embelia, a tremendously important medicinal plant of traditional and complementary medicine, is widely exploited. Therefore, determining the metabolic profile of Embelia is a prerequisite for developing new drugs. However, the metabolome of Embelia and the impact of different geographical regions on it is not known. Determining Embelia’s metabolic profile from different locations will provide its complete metabolic blend and reveal new compounds with potential medicinal importance. Moreover, it will allow the sustainable use of Embelia fruits. Therefore, in the current study, we analyzed the non-targeted metabolomic profiles of the Embelia fruits collected from different regions of the Western Ghats of India using liquid chromatography coupled with tandem mass spectroscopy (LC-MS/MS) to identify potential compounds for drug development, and attempt to reveal the relationship between the metabolomic and genotypic diversity.

## 2. Results

### 2.1. Molecular Identification of Fruit Samples Collected from Different Geographical Regions

**Figure 1:**
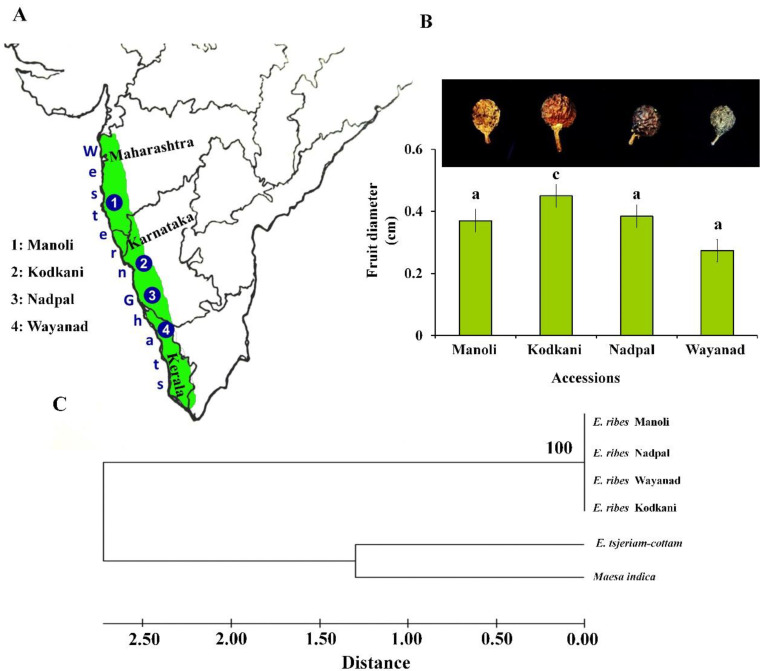
**Sampling locations of *Embelia ribes* (Embelia) fruit samples and their morphological and molecular identification.** (**A**) The highlighted portion of the magnified map of India depicting the four locations, namely: (1) Manoli (Maharashtra), (2) Kodkani (Karnataka), (3) Nadpal (Karnataka), (4) Wayanad (Kerala), across the range of Western Ghats. (**B**) Fruit samples corresponding to each of the four accessions show variation in their pigmentation and size (inset). The fruit diameter from the Kodkani accession is significantly more than that of other accessions (F_4,18_ = 12.91; *p* = 0.0002). Values are the mean (±SE) of five replicate fruits. Significant difference was determined by one way ANOVA and Tukey’s *post hoc* test at *p* ≤ 0.05. (**C**) Phylogenetic tree was constructed using the *maturase kinase* (*matk*) nucleotide sequences from different accessions of Embelia fruits with 1000 bootstrap values, and maximum composite likelihood method. It confirms the identity of the collected samples as *E. ribes* (Embelia). Sequences obtained from *E. tsjeriam-cottam* and *Maesa indica* leaves were used as an outgroup. Figures at the nodes indicate bootstrap values determined after bootstrapping the clusters 1000 times. The scale at the bottom of the tree quantifies the average distance between clusters.

Fruit samples collected across all the accessions (Figure 1A) were evaluated for their size and texture. Interestingly, fruits from Kodkani, Karnataka, and Wayanad, Kerala, are significantly larger and smaller than those from other accessions, respectively (Figure 1B). The fruit size of the Kodkani accession is significantly more than the other accessions (one way ANOVA, F_4,18_ = 12.91; *p* = 0.0002). Moreover, the fruits from Kodkani were spherical, whereas fruits from other locations were obovate. On the other hand, Wayanad fruits are the least wrinkled among other accessions (Figure 1B inset). Dissimilarities in Embelia fruit morphology necessitate the molecular identification of the collected samples. Chloroplast maturase K gene (*m*atK**) is one of the most variable coding genes of angiosperms and is used for the identification of plant species. Molecular identification by *matK* amplification is often used for the accurate genetic identification of an organism. Therefore, all the samples were evaluated by comparing the sequence homology of the *matK* gene with the reported sequence of *E. ribes* in the NCBI database (Table S1). The maximum percent identity and query cover threshold was more than 98% and 85%, respectively. Further, a phylogenetic tree was constructed with the *matK* sequences from all the Embelia accessions, *E. tsjeriam-cottam* (Roem. & Schult.) A. DC. and *Maesa indica* (Roxb.) A. DC. sequences (Figure 1C); *E. tsjeriam-cottam* and *M. indica* were used as outgroup. Embelia accessions collected across the Western Ghats did not show any genetic distances, whereas the outgroups showed a significant genetic distance from the Embelia accessions (Figure 1C). Our results confirmed the identity of the collected fruits as *E. ribes*.

### 2.2. Metabolic Profiles of Embelia Fruits Vary across the Accessions

The ethonopharmacological uses of Embelia fruits are different across geographical regions. The use of Embelia fruits for different ailments suggests that fruits from different locations may have a different blend of compounds that are useful for specific diseases. Therefore, we analyzed the metabolic profiles of Embelia fruits from different accessions. A total of 583 compounds are obtained from negative and positive modes from all the accessions (Data S1). The maximum number of compounds were present in the Kodkani accession (380 compounds); the Manoli accession is comprised of 352 compounds; Wayanad and Nadpal accessions are comprised of 346 and 341 compounds, respectively (Data S1).

The compounds present in all five replicates of any of the accessions are tentatively identified based on the formula. This trimmed data resulted in 149 compounds (Table S2), and the identity of 36 compounds was determined in MS/MS mode (Table 1, Figures S1 and S2). The identity of embelin, a prime compound of Embelia, from fruits samples was confirmed by HPLC (Figure S3). All further analysis was done with the 36 identified compounds. Similar to the raw data, Kodkani accession possesses the maximum number of compounds (34), followed by Wayanad (27), Manoli (8), and Nadpal (6). Interestingly, out of 36 identified compounds, 30 are reported for the first time from Embelia.

**Figure 2:**
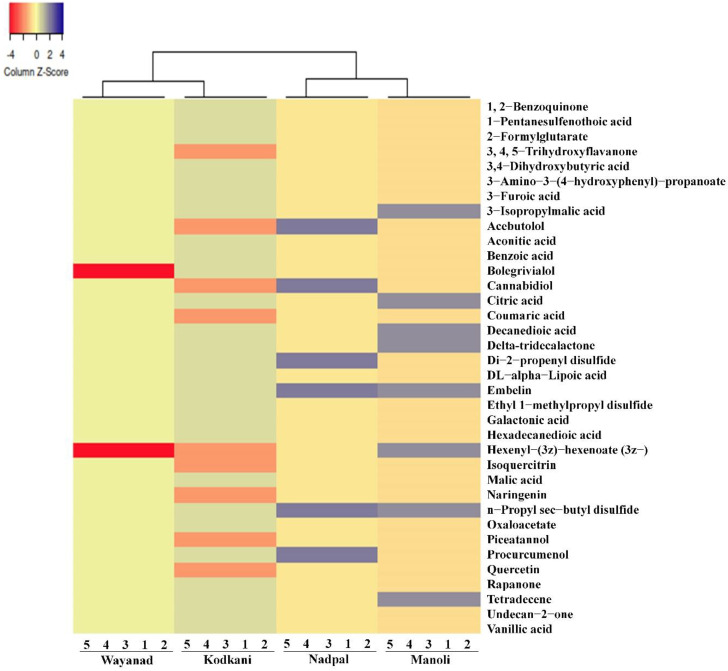
**The distribution of identified metabolites across the accessions.** The accessions showed a variation in the distribution of metabolites across the accessions. The color scheme at the top left corner codes for the Z-scores (−4 to +4) calculated over binary co-ordinates of the samples. The dendrogram at the top of the heatmap indicates the average linkage clustering computed between the samples based on the Manhattan distance.

**Figure 3:**
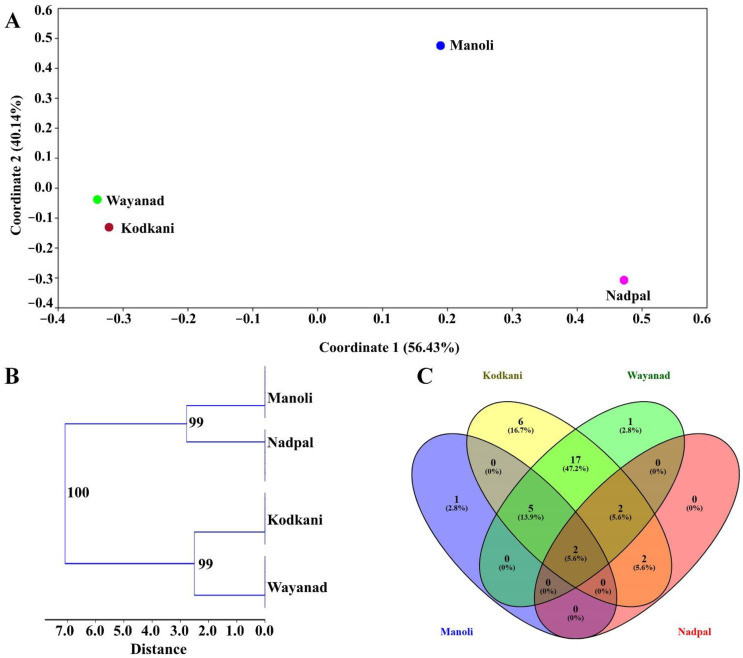
**The multivariate analysis of metabolomics data.** (**A**) The plot of principle coordinate analysis (PCoA) with identified metabolites across the accessions using the Jaccard coefficient and transformation exponent value 2. The percentage of the variation is captured in Coordinates 1 and 2. The values in parentheses show the percentages of the variation. The x- and y-axes are indicated by the eigenvalue scale. (**B**) A phylogenetic tree with the single linkage algorithm and Jaccard similarity index at the 1000 bootstrapping threshold. The tree shows the clusters among the accessions based on the similarities in the metabolites in each of the five samples of a given accession. Bootstrap values of the respective clusters are depicted at each tree node. (**C**) The Venn diagram depicted the number of compounds shared and uniquely present along with all the combinations. Kodkani and Wayanad accessions show the maximum number of shared compounds, whereas Manoli and Nadpal accessions show the least number of shared compounds. Similarly, Kodkani accession showed a maximum (six) number of uniquely present compounds. However, Nadpal accession does not have any unique one.

A heatmap was created with the identified compounds to visualize the compound diversity (Figure 2). Principal coordinate (PCoA) and cluster analysis determined the relationship between the accessions. PCoA ordination captured maximum variation in coordinates 1 and 2, where Kodkani and Wayanad accession form a group, but Manoli and Nadpal accession is placed separately (Figure 3A). Like the PCoA, the cluster analysis also placed Kodkani and Wayanad accessions in the same clade (Figure 3B); Manoli accession is close to this cluster but in a separate clade. The three accessions are grouped as one large cluster that is further connected to a separated clade of Nadpal accession. The high bootstrap value (99) of the cluster of Kodkani and Wayanad accessions signifies their metabolic similarity compared to the other accessions.

We look further to find the compound similarities across the accessions by creating the Venn diagram (Figure 3C, Table 1). We found that only two compounds, embelin and n-propyl sec-butyl disulfide, are present in all the accessions. The number of unique compounds present in Manoli (hexenyl-(3z)-hexenoate (3z-)) and Wayanad (bolegrivialol) accessions is one; however, Kodkani accession possesses six unique compounds (Figure 3C, Table 1) that belong to the phytophenols, namely 3,4,5-Trihydroxyflavanone, Dihydro 3-coumaric acid, quercetin, isoquercitrin, naringenin, and piceatannol (Table 1). On the contrary, Nadpal accession did not show the occurrence of any unique compounds. Moreover, through the pairwise analysis of similarity percentage (SIMPER test; Bray-Curtis dissimilarity index) in the compounds at a 50% cut-off, we identified 15 compounds responsible for the grouping accessions (Table S3).

### 2.3. Identified Compounds Represent Major Chemical Groups and Potential Medicinal Properties

**Figure 4:**
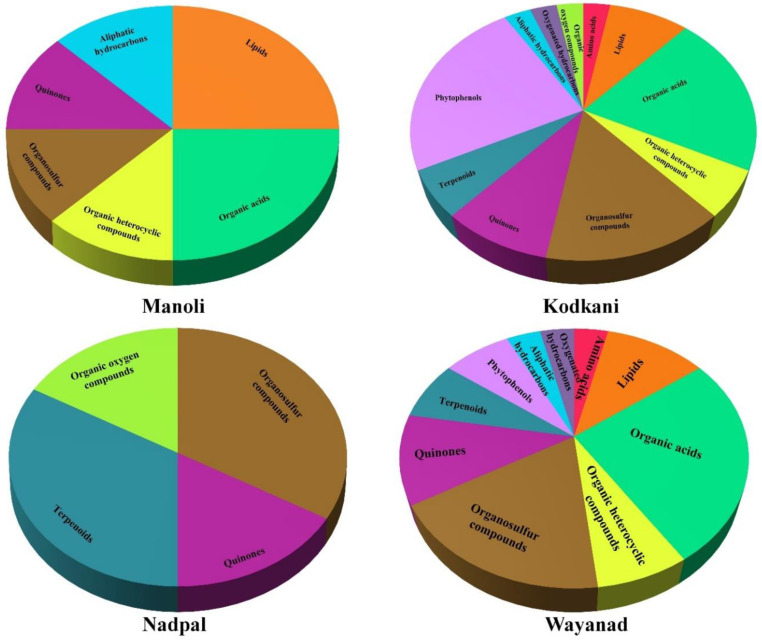
**Distribution of chemical classes across the accessions**. Identified compounds were grouped according to their compound classes. A total of 11 classes of compounds were found. Kodkani accession showed all 11 groups, followed by ten groups in Wayand, six in Manoli, and four in Nadpal.

Compounds identified by MS/MS analysis were classified according to their chemical classes, which include quinones, phytophenols, terpenoids, organosulfur compounds, organic acids, aliphatic hydrocarbons, lipids, oxygenated hydrocarbons, organic oxygen compounds, organic heterocyclic compounds, and amino acids (Figure 4). Interestingly, Kodkani accession possesses all the 11 groups of compounds that suggest it as the most diverse accession among all. Compounds present in Manoli, Nadpal, and Wayanad accessions represent six, four, and 10 compound groups, respectively. Quinones and organosulfur compounds are present across all the accessions; however, different accessions show a different blend of compounds (Figure 4). Reports suggest that most identified compounds are active against different ailments mainly cancer, cardiac disorder, and oxidative stress (Table S4). There are a few new compounds, namely 1-pentanesulfenothoic acid, 2-formylglutarate, ethyl 1-methylpropyl disulfide, galactonic acid, 3-isopropylmalic acid, and 3-furoic acid whose functions are not known yet (Table S4).

### 2.4. Accessions of Embelia Are Genetically Diverse

**Figure 5:**
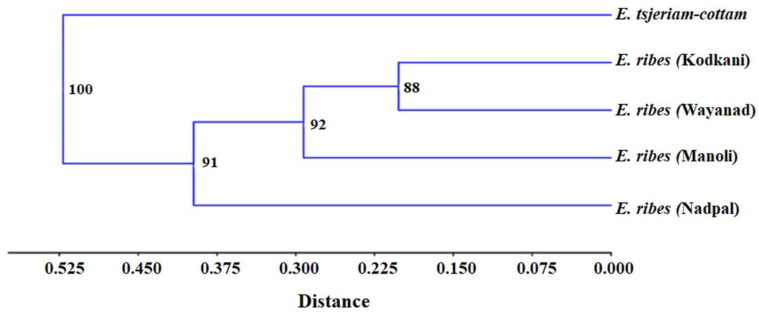
**Genotypic diversity among *Embelia ribes* accessions from different geographical regions.** After scoring the amplified products on Embelia and *E. tsjeriam-cottam* by nine IISR primers, obtained binary data was used to generate a phylogenetic tree. *E. tsjeriam-cottam* was used as an outgroup. Wayanad and Kodkani accession share the same clade, whereas the Manoli accession connects the Nadpal accession with Wayanad and Kodkani accessions. The outgroup is forming a separate clade. The scale at the bottom of the phylogenetic tree quantifies the Jaccard distance between the genotypes. Numbers at each tree node indicate the bootstrap value of the respective cluster computed at the 1000 bootstrapping threshold.

Differences in fruit phenotype hinted towards the genetic diversity of Embelia across the accessions. Moreover, genetic variability is one of the prime factors that tunes plants’ metabolic profiles. Therefore, to check if the genotypic diversity orchestrates the metabolic variations across the Embelia accessions, we examined the genotypic diversity of Embelia accessions with inter simple sequence repeat (ISSR) markers. As demonstrated in a previous study, we used nine ISSR markers (Table S5) that are polymorphic for Embelia plants [18]. All the markers yielded robust and reproducible polymorphic amplification patterns. Ninety-four bands were generated, with an average of 10.4 products per primer. Among these, 90 bands (95.74%) were polymorphic and four (4.25%) were monomorphic (Table S6). The number of polymorphic bands ranged from six (in the case of primer 816) to 13 (in the case of primer 857) (Table S6). While the primers 809, 857, and 881, had a high percentage of polymorphic bands (PPB), primer 816 showed the lowest PPB (6.38%) (Table S6). A hierarchical cluster was generated with the binary matrix using an unweighted neighbor-joining method with Jaccard’s coefficient of dissimilarity that clustered the accessions into two main clades, and the clades having Embelia accessions are further formed into three clades (Figure 5). The presence of Wayanad and Kodkani accessions in the same clade suggests that they share the maximum similarity. Interestingly, Manoli accession shares similarities with Wayanad, Kodkani, and Nadpal accessions. *E. tsjeriam-cottam* was used as an outgroup that showed significant differences with Embelia accessions and clustered separately. In the Embelia cluster, the high percentage of polymorphism suggests high genetic variability among the accessions under study.

### 2.5. Metabolic Diversity in Embelia Fruits Correlates with Genotypic Diversity

As the cluster analysis with metabolite and ISSR matrix showed similar grouping of the accessions, we hypothesized that metabolomic variation in different Embelia accession is due to their genotypic diversity. Therefore, we performed Mantel’s test with both matrices. Mantel’s test showed significant positive correlation (R = 0.799; *p* = 0.043) between genotypic and metabolic data matrix. Therefore, we conclude that their genotype governs the metabolic diversity in Embelia fruits along the Western Ghats accessions.

## 3. Discussion

The different parts of Embelia plants, especially the fruits, are predominantly used in folklore medicine. However, a detailed study of the active principles and their benefits and risks need to be evaluated to integrate traditional medicine practices into healthcare systems. However, limited information on the chemical profiles of Embelia fruits made it challenging to predict the compound or blend of compounds presumably responsible for a specific ailment’s remedy. The geographical region-specific use of Embelia fruits against various ailments stemmed us to study their metabolic profiles across different geographical regions. Our study is the first attempt at the non-targeted metabolic profiling of Embelia fruits that reveals Embelia’s chemical diversity associated with their geographical location, genotypic diversity, and ethnopharmacology. Moreover, new compounds can be explored further for their functionality against ailments.

Our study highlighted that the metabolic blend of Embelia fruit varies across geographical regions. The climatic and edaphic differences restricted to a particular geographical area could govern the chemistry of a plant [8]. Chemical diversity in the plants also validates the region-specific use of the Embelia fruits against various ailments. Unfortunately, the metabolic profile of Embelia fruit is mainly unknown, as most of the studies focused on the major chemical groups, namely, quinones, flavonoids, essential oils, etc. [10,19,20]. We demonstrate a spectrum of varied compounds across the different accessions from the Western Ghats of India. On a geographical scale, two Karnataka accessions, Nadpal and Kodkani, are situated in proximity with an aerial distance of 132.35 km. Therefore, these accessions share fewer climatic and topographical variations. However, the multivariate analysis of the chemical compositions did not group Kodkani and Nadpal; surprisingly, Kodkani is grouped with Wayanad accession, which is 361.38 km (aerial distance) away from the Kodkani accession. Interestingly, the Manoli accession is 361.38 km and 632.65 km away from Kodkani and Wayanad accession, respectively, and showed similar percent similarity in the compound profile with both locations. These observations question the contribution of geographical parameters to the chemical diversity among different locations. Plant populations may respond genetically to differential selection pressures brought by environmental factors, such as climatic heterogeneity and geographic isolation [21], and plants’ genetic diversity could be the major player in their observed metabolic diversity. An analysis of genetic diversity with ISSR markers revealed high genetic variation across the accessions. Embelia requires particular geographical conditions to grow and distribute in patches; therefore, high genetic variability among the Embelia accessions contradicts the fact that geographically restricted species tend to have less genetic variation than the standard widespread species [22]. On the other hand, reports also suggest that species distributed in patches showed more significant differentiation than the more continuously distributed species [23]. Moreover, species with small populations and less genetic variability are vulnerable to extinction [24]. Therefore, presumably, being a sparsely distributed and threatened species, Embelia showed high genetic variation among the accessions, making it less prone to extinction. A significant correlation between the matrices of ISSR and metabolic data confirms that metabolic diversity in Embelia fruit is attributed to its genetic variability.

In traditional and folklore medicine, Embelia fruits are mainly used as antihelmintic, antidyspepsia, appetizer, mild-laxative, carminative, alexiteric, and antipyretic. However, to treat a particular disorder successfully, the presence of a specific compound or a blend of compounds is necessary. Chemical variation in the plant sample can influence the effectiveness of the formulated drugs against a particular disease. Therefore, the selecting of raw materials based on their chemical composition is a prerequisite. Variations in the traditional use maybe rely on this hypothesis. Therefore, we categorized the identified compounds from Embelia fruits according to their functionality. The compounds having antipyretic, antihelmintic, and antioxidant activity, is fairly equally distributed throughout all the accessions (Table S4); this supports the use of Embelia fruits as antipyretic and antihelmintic across all regions. In folklore medicine, Embelia fruits are used for the treatment of cancer, mainly in Karnataka [25]. Interestingly, Embelia fruits from Kodkani, one of the Karnataka accessions, are dominated by compounds with anticancerous properties. Previous reports have designated embelin and its derivatives from Embelia as anticancerous compounds; however, we identified other compounds with similar properties but different mechanisms of action. Moreover, piceatannol, a unique compound present in Kodkani accession, is a hypoglycemic agent [26,27]; this correlates with the use of Embelia fruits against polyuria, which may cause by the hyperglycemic condition. Compounds that control cardiac rhythm and have anticoagulant properties from the Karnataka accessions also justified the local use of Embelia fruits against cardiac ailments. There is a growing importance of phytochemicals as male contraceptives because the anti-fertility effects of phytochemicals are reversible. For instance, sterility induced by embelin in male albino rats was reversed within 15–30 days [28]. Therefore, the chemical constituents showed that anti-fertility properties are clinically crucial for developing male contraceptives [29]. Polyphenols from *Mucuna urens.* can inhibit the endogenous gonadotrophic activity in male albino rats [30]; therefore, the presence of different phytophenols can be examined as a potential target to develop male contraceptives. The current study is the first non-targeted metabolomics study of Embelia fruits that highlighted the region-specific compounds blend governed by intraspecific genetic variations. We found a few compounds, namely 1-pentanesulfenothoic acid, 2-formylglutarate, ethyl 1-methylpropyl disulfide, galactonic acid, 3-isopropylmalic acid, and 3-furoic acid, whose functions are unknown. Testing these compounds against mammalian cell lines may reveal their new potential use. Moreover, the generated metabolomic profiles can be utilized as a metabolic fingerprint of the accessions during the formulation of new herbal drugs. The identification of favorable chemotypes will support quality control and the sustainable use of the plant materials.

## 4. Materials and Methods

### 4.1. Plant Material

Embelia fruits were used for metabolic and genetic diversity analysis. Fruits collected by the local villagers from the forest around Manoli, Kolhapur, were procured from local roadside sellers; fruits were also collected from the forests of Nadpal, Karnataka (Nadpal accession), Kodkani, Karnataka (Kodkani accession), and Wayanad, Kerala (Wayanad accession). The leaf tissues of *E. tsjeriam-cottam* and *Maesa indica* were collected from the Savitribai Phule Pune University (SPPU) medicinal garden and used for genetic diversity analysis as an outgroup. Specimen samples from each location and genotype are deposited at the Department of Botany, SPPU.

### 4.2. Metabolite Extraction and LC-MS/MS Acquisition

Dried Embelia fruits were pulverized and ~40 mg of tissue was extracted in 400 µL 70% methanol (*v*/*v*) spiked with the internal standard formononetin (2 µg ml^−1^) by vortexing overnight. The extracts were centrifuged for 20 min at 13,000 rpm at 4 °C and the supernatant was collected. Further, the supernatant was filtered through microfilters (20 µm) and subjected to LC-QTOF-MS/MS (Agilent Technologies, Stuttgart, Germany) for analysis.

A Zorbax C18 (1.7 μm, 2.1 mm × 100 mm) column was used to separate metabolites. The mobile phases used were 0.1% formic acid (solvent A) and acetonitrile containing 0.1% formic acid (solvent B). Separation was achieved at a flow rate of 0.3 mL min^−1^ and a column temperature of 25 °C. The solvent gradient profile followed an initial isocratic separation with 95% A (1 min) followed by the gradient of solvent A 5%, B 95% until 12.00 min, solvent A 95%, and B 5%, until 12.5 min, that extended up to 16th minute. The mass spectrometer was used in centroid mode for both negative and positive ionization with a pump limit of 1 min, draw speed of 200 µL min^−1^, and eject speed of 400 µL min^−1^. Samples were analyzed with an injection volume of 10 µL where the pressure limit in the column was maintained at 0 to 800 bar, retention time (RT) exclusion tolerance was maintained at (±) 0.2 min, and ion source (dual ESI) with a limit of two precursors per minute.

### 4.3. LC-MS/MS Data Analysis

The personal compound database and library (PCDL) Mass Hunter Qualitative Analysis BO.07.00 tool (Agilent technologies) was used to analyze the compound spectra. The PCDL library was made by accessing mainly the METLIN database, all possible libraries for the medicinally important and general plant metabolites and phytochemicals associated with medicinally essential plants. MS/MS acquisition was performed with five replicate fruits to examine the biological variations within the accession and reproducibility. To eliminate the background contaminant compounds from the analysis, only the compounds with an abundance greater than 10,000 counts and a score of more than 70 are considered. These trimmed compounds were screened against PCDL library and resulted in 3574 compounds. Compounds were identified using the ‘find by formula’ function in the software package with a mass threshold of 7 ppm and a peak distance threshold of 10 ppm in MS mode. The resulting 583 compounds were further trimmed based on their presence in all five replicates of any accessions resulting in 149 compounds across the accessions. These compounds were analyzed in MS/MS mode for the presence of the daughter ions to confirm their identity. Finally, 36 compounds were identified in MS/MS mode (Table 1, Figures S1 and S2).

### 4.4. Genomic DNA Isolation, and Polymerase Chain Reaction (PCR)

DNA was isolated using a CTAB-STE method by Doyle and Doyle with modifications [31,32]. Isolated DNA was dissolved in TE buffer and quantified in Nanodrop (ND-1000 Spectrophotometer). 100 ng of isolated DNA was amplified using the *matK* gene-specific primer pairs (forward 5′ TCCGCTACTGGGTAAAAGATG 3′, reverse 5′ ATATCGCCCCAAATCGGTCA 3′) and nine inter simple sequence repeat (ISSR) primers (Table S5) in a thermocycler (Biorad) with one cycle of initial denaturation (95 °C for 2 min), 40 cycles (*matk*) or 34 cycles (ISSR) of denaturation (95 °C for 15 s), annealing (60 °C for *matK* or 50–60 °C for ISSR) (Table S5) for 1 min, extension (72 °C for 30 s), and one cycle of final elongation (72 °C for 7 min). The amplified DNA was separated on a 1.5% agarose gel and documented on a gel documentation system (Biorad).

### 4.5. Sequencing and Data Analysis

Amplified products by *matK* primers were outsourced for Sanger sequencing (Bioserve). Obtained sequences were analyzed using the CROMAS (version 2.6.6) software and aligned using multiple sequence comparison by log expectation (MUSCLE www.ebi.ec.uk (accessed on August 2021). Homology searches were performed within the Genbank non-reductant database using the BLAST algorithm (http://www.ncbi.nim.nih.gov./BLAST/ (accessed on 31 August 2021). All the *matK* sequences generated have been deposited in the NCBI GenBank database (Accession numbers: Bank It2604113: Seq1-OP081086, Seq2-OP081087, Seq 3-OP081088, Seq4-OP081089, Seq5-OP081090, and Seq6-OP081091). Evolutionary analyses were conducted in the MEGA X (version 10.0.5) to generate an optimal tree with a sum of branch length 6.743 and a bootstrap of 1000 replicates. The evolutionary distances were computed using the maximum composite likelihood method with Tamura 3 parameter model and are in the units of the number of base substitutions per site. All ambiguous positions were removed for each sequence pair using the pairwise deletion option. There was a total of 831 positions in the final dataset. The tree was drawn to scale, with branch lengths in the same units as the evolutionary distances.

### 4.6. Analysis of Genetic Diversity and Construction of Phylogenetic Tree

Amplicons obtained from each ISSR primer are scored for their presence (1) and absence (0) across the accessions to analyze the genetic diversity. The percentage of polymorphism and polymorphic bands were calculated across the accessions and the used primers, respectively [18]. The binary data matrix was converted into a genetic similarity matrix, and a neighbor-joining tree was obtained with maximum likelihood using the Jaccard coefficient with DARwin 6 (6.0.21) genetic analysis tool. The length of the obtained tree branches was verified with the unweighted neighbor-joining method.

### 4.7. Statistical Analysis

Data were analyzed by one-way ANOVA followed by Tukey’s *post hoc* test, and the significance was determined at *p* ≤ 0.05. Multivariate analysis (PCoA, clustering, SIMPER) of metabolomics data was done in PAST 3 [33]. DARwin 6 genetic analysis tool [34] and MEGA X were used for genetic diversity analysis.

## Figures and Tables

**Table 1:** **Compounds identified from different Embelia accessions by LC-MS/MS.** Total of 36 compounds were identified based on the fragment ion spectra in both negative and positive ionization modes. 1–33 in negative ionization mode and 34–36 in positive ionization mode (M—Manoli, K—Kodkani, N—Nadpal, W—Wayanad).

Sr. No.	Molecular Formula	Abundance (%)	Adducts	Mass	*m*/*z*	RT	Score	Compound Name	M	K	N	W
1	C_6_ H_4_O_2_	167.0353 (100), 108.0219 (84.96), 153.0198 (16.49), 123.0113 (0.46)	(M+HCOO-), (M+CH3COO-)	108.0211	167.0556	9.141	98.87	1,2-Benzoquinone	**-**	**+**	**-**	**+**
2	C_5_H_12_S_2_	195.0514 (100), 129.0197 (57.31), 177.0409 (6.16), 159.0312 (7.27)	(M-H-), (M+CH3COO-)	136.0387	135.0307	2.922	81.86	1-pentanesulfenothoic acid	**-**	**+**	**-**	**+**
3	C_6_H_8_O_5_	111.0092 (100), 125.0248 (11.05), 143.0351 (7.81), 169.0146 (23.32)	(M-H-), (M+CH3COO-), (M+HCOO-)	160.0372	205.0361	6.443	97.15	2-Formylglutarate	**-**	**+**	**-**	**+**
4	C_15_H_12_O_5_	271.061 (100), 151.0035 (32.56), 119.051 (13.26), 107.0127 (2.66), 177.0177 (4.73)	(M-H-), (M+CH3COO-), (M+HCOO-)	272.0685	271.0626	10.89	93.68	3,4,5-Trihydroxyflavanone	**-**	**+**	**-**	**-**
5	C_4_H_8_O_4_	136.8745 (100), 134.8767 (72.48), 119.3337 (30.23), 138.8695 (33.82), 112.4422 (23.18)	(M-H-), (M+CH3COO-), (M+HCOO-)	120.0423	179.0566	3.756	94.87	3,4-Dihydroxybutyric acid	**-**	**+**	**-**	**+**
6	C_9_H_11_NO_3_	163.0407 (100), 180.068 (82.43),135.0464 (91.86), 119.0486 (34.69), 121.0316 (3.34)	(M-H-), (M+CH3COO-)	181.0739	180.0672	4.68	84.77	3-Amino-3-(4-hydroxyphenyl) propanoate	**-**	**+**	**-**	**+**
7	C_5_H_4_O_3_	111.0091 (100), 112.9853 (59.93), 105.6042 (10.74), 137.3377 (5.76), 168.1806 (11.1)	(M-H-), (M+CH3COO-)	112.016	111.0093	3.256	85.76	3-Furoic acid	**-**	**+**	**-**	**+**
8	C_7_H_12_O_5_	119.0352 (100), 163.0618 (18.61), 101.0256 (5.33), 221.0677 (16.91)	(M-H-), (M+CH3COO-)	176.0685	221.0675	6.781	93.91	3-Isopropylmalic acid	**+**	**+**	**-**	**+**
9	C_6_H_6_O_6_	111.0089 (100), 101.049 (0.93), 117.0174 (4.29), 129.0177 (1.58), 155.0332 (5.15), 173.0828 (2.73), 175.0604 (12.66), 191.02185(0.34)	(M-H-),	174.0164	173.0097	6.363	97.02	Aconitic acid	**-**	**+**	**-**	**+**
10	C_7_H_6_O_2_	121.0298 (100), 120.0214 (20.48), 119.9136 (0.35), 109.8534 (0.28)	(M-H-), (M+CH3COO-)	122.0368	121.0356	9.141	96.01	Benzoic acid	**-**	**+**	**-**	**+**
11	C_28_H_40_O_4_	439.2885 (100), 279.2339 (8.98), 101.0243 (2.78), 170.8337 (1.48), 131.0358 (0.79), 161.0442 (0.84)	(M-H-), (M+CH3COO-)	440.2954	439.2854	15.52	86.76	Bolegrevilol	**-**	**-**	**-**	**+**
12	C_6_H_8_O_7_	111.0093 (100), 129.0197 (5.64), 131.9995 (0.18), 154.9984 (0.71), 173.0095 (1.3)	(M-H-), (M+CH3COO-)	192.027	191.0201	4.259	95.64	Citric acid	**+**	**+**	**-**	**+**
13	C_9_H_8_O_3_	119.0509 (100), 163.0407 (8.71), 117.9266 (0.38), 121.0308 (2.97)	(M-H-), (M+CH3COO-)	164.0484	163.0412	8.88	93.57	Coumaric acid	**-**	**+**	**-**	**-**
14	C_10_H_18_O_4_	201.1134 (100), 183.1021 (59.57), 139.7911 (65.43), 140.1177 (13.75), 141.11 (5.96), 157.0852 (5.47)	(M-H-)	202.1205	201.1143	9.982	95.36	Decanedioic acid	**+**	**+**	**-**	**+**
15	C_13_H_24_O_2_	213.1868 (100), 195.1759 (2.92), 193. 1605 (0.16), 211.3199 (0.06), 257.1764 (0.46), 196.179 (0.83)	(M-H-), (M+CH3COO-)	212.1776	271.1918	15.268	85.1	Delta-tridecalactone	**+**	**+**	**-**	**+**
16	C_6_H_10_S_2_	111.0093 (100), 191.0208 (10.89), 173.9144 (0.08), 154.9984 (0.71), 147.0297 (0.9), 130.9991 (0.88)	(M-H-)	146.0224	191.0199	4.26	81.97	Di-2-propenyl disulfide	**-**	**+**	**+**	**+**
17	C_8_H_14_O_2_S_2_	111.0095 (100), 125.0248 (12.2), 169.0149 (30.75), 101.0249 (0.1), 143.0356 (6.94), 154.9997 (2.13), 205.0366 (2.19), 173.0102 (1.98)	(M-H-), (M+CH3COO-)	206.0435	205.0365	6.365	80.91	DL-alpha-Lipoic acid	**-**	**+**	**-**	**+**
18	C_17_H_26_O_4_	293.1776 (100), 265.1822 (10.08), 152.0117(23.46), 195.1762 (0.05), 223.1980 (0.02), 265.1817 (0.98), 275.1672 (0.04), 179.0336 (0.02), 151.0399 (0.73), 163.0405 (0.03), 165.0197 (0.58), 193.1603 (0.01)	(M-H-)	294.1831	293.1771	14.42	94.7	Embelin	**+**	**+**	**+**	**+**
19	C_6_H_14_S_2_	195.0514 (100), 129.0197 (63.16), 101.0232 (1.78), 100.0129 (1.67)	(M-H-), (M+CH3COO-)	150.0537	195.0514	2.91	83.13	Ethyl-1-methylpropyl disulfide	**-**	**+**	**-**	**+**
20	C_6_H_12_O_7_	195.0541 (100), 129.0197 (63.16), 111.0089 (1.93), 101.0232 (1.78), 141.0172 (1.17), 100.0129 (1.67), 151.06 (2.87), 159.02 (4.72), 177.04 (7.5)	(M+CH3COO-)	196.0583	195.0514	2.92	96.12	Galactonic acid	**-**	**+**	**-**	**+**
21	C_16_H_30_O_4_	285.2076 (100), 195.1734 (0.69), 160.314 (0.24), 151.98(0.44)	(M-H-), (M+CH3COO-)	286.2144	285.2083	11.57	94.66	Hexadecanedioic acid	**-**	**+**	**-**	**+**
22	C_21_H_20_O_12_	463.0907 (100), 301.0354 (9.29), 316.0235 (4.15), 300.2459 (7.06), 271.0264 (1.1), 178.9978 (0.59), 287.0243 (0.21)	(M-H-)	464.0948	523.1085	8.79	76.8	Isoquercitrin	**-**	**+**	**-**	**-**
23	C_4_H_6_O_5_	115.0039 (100), 133.0137 (25.99), 134.0181(0.08), 103.0701 (2.41), 105.3022 (3.11)	(M-H-)	134.0215	133.0148	3.16	98.24	Malic acid	**-**	**+**	**-**	**+**
24	C_15_H_12_O_5_	271.062 (100), 151.0037 (37.98), 119.0509 (10.92), 177.0197 (5.23), 107.0142 (4.58)	(M-H-)	272.0697	271.0623	10.90	91.8	Naringenin	**-**	**+**	**-**	**-**
25	C_7_H_14_S_2_	119.0352 (100), 163.0618 (18.61), 221.0677 (16.91), 101.0256 (5.33)	(M-H-), (M+CH3COO-)	162.0537	221.0675	6.78	85.79	N-propyl sec-butyl disulfide	+	+	+	+
26	C_4_H_4_O_5_	111.0092 (100), 103.1223 (0.39), 106.2887 (0.38), 129.01 (7.82), 191.0208 (10.89)	(M-H-), (M+CH3COO-)	132.0059	191.0201	4.26	98.76	Oxaloacetate	**-**	**+**	**-**	**+**
27	C_14_H_12_O_4_	261.0395 (100), 103.0404 (17.34), 111.0115 (25.8), 135.0417 (13.41), 137.0252 (59.38), 148.0374 (31.73), 167.0366 (27.76), 231.0277 (50.05)	(M+CH3COO-)	244.0736	303.0885	8.37	94.96	Piceatannol	**-**	**+**	**-**	**-**
28	C_15_H_22_O_2_	293. 1772 (100), 152.01 (1.17), 124.0171 (1.09), 265.1818 (0.98), 166.0278 (0.29), 275.1665 (0.04), 195.1756 (0.01)	(M+CH3COO-), (M+HCOO-)	234.1626	293.1776	14.51	95.39	Procurcumenol	**-**	**+**	**+**	**+**
29	C_15_H_10_O_7_	301.0363 (100), 107.0142 (1.63), 151.0034 (19.11), 121.0294(2.17), 178.9991(14.1)	(M-H-)	302.0427	301.0367	10.15	88.89	Quercetin	**-**	**+**	**-**	**-**
30	C_19_H_30_O_4_	321.2089 (100), 124.0169 (0.71), 152.0117 (1.01), 293.2134 (0.66), 166.0257 (0.18), 317.8458 (0.09)	(M-H-)	322.2144	321.2093	13.16	92.93	Rapanone	**-**	**+**	**-**	**+**
31	C_14_H_28_	255.2335 (100), 210.9515 (6.19), 134.0953 (3.02), 119.037 (4.12), 112.98 (2.76)	(M+CH3COO-)	196.2191	255.2338	0.572	96.94	Tetradecene	**+**	**+**	**-**	**+**
32	C_11_H_22_O	229.1825 (100), 112.9864 (0.85), 151.9661 (0.95), 183.1758 (43.74)	(M+CH3COO-), (M+HCOO-)	170.1675	229.1817	11.31	93.1	Undecan-2-one	**-**	**+**	**-**	**+**
33	C_8_H_8_O_4_	167.0354 (100),108.0222 (95.03), 168.8361 (83.37), 152.0119 (14.39), 128.0691 (3.21), 125.0234 (1.95)	(M-H-)	168.0429	167.0356	9.14	83.19	Vanillic acid	**-**	**+**	**-**	**+**
34	C_18_H_28_N_2_O_4_	127.1205 (100), 129.1004 (58.35), 145.1369 (55.08), 319.1974 (15.5), 273.1822 (30.12), 147.1169 (30.35), 175.1199 (44.14), 301.1838 (12.14), 109.1038 (27.28)	(M+H+)	336.2049	337.2135	3.16	75.88	Acebutolol	**-**	**+**	**+**	**-**
35	C_21_H_30_O_2_	127.1204 (100), 129.1005 (55.48), 145.1367 (55), 175.1199 (46.27), 147.1168 (28.57), 109.1039 (27.64), 157.1043 (14.87), 111.084 (3.29), 193.1358 (9.45), 163.1531 (4.65), 139.0882 (11.36), 273.1821 (28.48), 301.1835 (12.39), 291.1975 (7.41), 319.1984 (15.9)	(M+Na+)	314.2246	337.2134	4.17	76.21	Cannabidiol	**-**	**+**	**+**	**-**
36	C_12_H_20_O_2_	109.1032 (100), 219.1348 (16.32), 204.1907 (16.05), 202.1745 (25.12), 187.1584 (22.13), 169.1432 (52.16), 151.1255 (9.44), 145.1362 (3.92), 111.1186 (6.99), 106.1057 (0.58), 127.1189 (29.49)	(M+Na+)	196.1463	219.1353	2.83	73.22	Hexenyl-(3z)-hexenoate (3z-)	**+**	**-**	**-**	**-**

## Data Availability

All the *matK* sequences generated have been deposited in the NCBI GenBank database (Accession numbers: Bank It2604113: Seq1-OP081086, Seq2-OP081087, Seq 3-OP081088, Seq4-OP081089, Seq5-OP081090, and Seq6-OP081091).

## Supplementary Materials

The following supporting information can be downloaded at: https://www.mdpi.com/article/10.3390/plants11212861/s1. Data S1: compounds obtained from negative and positive modes from all the accessions in LC-MS/MS; Table S1: NCBI blast results for *matK* sequences; Table S2: compounds identified tentatively in both positive and negative mode of LC-MS; Table S3: contribution of differentially regulated compounds in groupings of the accessions; Table S4: reported functions of the identified compounds from Embelia accessions; Table S5: list of ISSR primers with their nucleotide sequences and annealing temperatures used for amplification; Table S6: analysis of genetic diversity in Embelia accession by ISSR primers. Figure S1: representative LC-MS chromatograms (BPC) from fruits samples from all accessions; Figure S2: MS and MS-MS spectra for 36 identified compounds from different accessions of Embelia fruits; Figure S3: confirmation of Embelin from fruit samples.

## References

[1] Burman N.L. (1768). Nicolai Laurentii Burmanni Flora Indica.

[2] Lal B., Mishra N. (2013). Importance of Embelia ribes: An update. Int. J. Pharm. Sci. Res..

[3] Udayan P., Harinarayanan M., Tushar K., Balachandran I. (2008). Some Common Plants Used by Kurichiar Tribes of Tirunelli Forest, Wayanad District, Kerala in Medicine and Other Traditional Uses. Ind. J. Tradit. Knowl..

[4] Thyloor R. (2018). Phytochemical analysis of Embelia ribes seeds for antimicrobial activities. J. Med. Plants.

[5] Chopda M., Mahajan R. (2009). Wound healing plants of Jalgaon district of Maharashtra state, India. Ethnobot. Leafl..

[6] Albert A., Sareedenchai V., Heller W., Seidlitz H.K., Zidorn C. (2009). Temperature is the key to altitudinal variation of phenolics in Arnica montana L. cv. ARBO. Oecologia.

[7] Karimi A., Krahmer A., Herwig N., Hadian J., Schulz H., Meiners T. (2020). Metabolomics approaches for analyzing effects of geographic and environmental factors on the variation of root essential oils of *Ferula assa-foetida* L. J. Agric. Food Chem..

[8] Saurabh K.B. (2011). Total phenolic content and antioxidant activity of extracts of Bridelia retusa Spreng Bark: Impact of dielectric constant and geographical location. J. Med. Plants Res..

[9] Namdeo A.G., Sharma A., Fulzele D.P., Mahadik K.R. (2010). Influence of geographical and climatic conditions on camptothecin content of Nothapodytes nimmoniana. Rec. Nat. Prod..

[10] Kamble V., Attar U., Umdale S., Nimbalkar M., Ghane S., Gaikwad N. (2020). Phytochemical analysis, antioxidant activities and optimized extraction of embelin from different genotypes of *Embelia ribes* Burm f.: A woody medicinal climber from Western Ghats of India. Physiol. Mol. Biol. Plants.

[11] Atlabachew M., Mehari B., Combrinck S., McCrindle R. (2017). Single-step isolation of embelin using high-performance countercurrent chromatography and determination of the fatty acid composition of seeds of *Embelia schimperi*. Biomed. Chromatogr..

[12] Kamble G.S., Torane R.C., Ghayal N.A., Tambe A.S., Deshpande N.R., Salvekar J.P. (2010). GC-MS study of fatty acids and esters from *Embelia basal*. J. Pharm. Res..

[13] Puiggròs F., Solà R., Bladé C., Salvadó M.-J., Arola L. (2011). Nutritional biomarkers and foodomic methodologies for qualitative and quantitative analysis of bioactive ingredients in dietary intervention studies. J. Chromatogr. A.

[14] Ibáñez C., Valdés A., García-Cañas V., Simó C., Celebier M., Rocamora-Reverte L., Gómez-Martínez Á., Herrero M., Castro-Puyana M., Segura-Carretero A. (2012). Global Foodomics strategy to investigate the health benefits of dietary constituents. J. Chromatogr. A.

[15] Hoffmann J.F., Carvalho I.R., Barbieri R.L., Rombaldi C.V., Chaves F.C. (2017). *Butia* spp. (Arecaceae) LC-MS-based metabolomics for species and geographical origin discrimination. J. Agric. Food Chem..

[16] Brinckmann J.A. (2015). Geographical indications for medicinal plants: Globalization, climate change, quality and market implications for geo-authentic botanicals. World J. Tradit. Chin. Med..

[17] Sampaio B.L., Edrada-Ebel R., Da Costa F.B. (2016). Effect of the environment on the secondary metabolic profile of Tithonia diversifolia: A model for environmental metabolomics of plants. Sci. Rep..

[18] Chrungoo N., Rout G., Balasubramani S., Rajasekharan P., Haridasan K., Rao B., Manjunath R., Nagduwar G., Venkatasubramanian P., Nongbet A. (2018). Establishing taxonomic identity and selecting genetically diverse populations for conservation of threatened plants using molecular markers. Curr. Sci..

[19] Dwivedi S., Ghatuary S.K., Prasad S., Jain P.K., Parkhe G. (2019). Phytochemical Screening and In Vivo Anti-inflammatory Activity of Hydroalcoholic Extract of Embelia Ribes Burm. F. J. Drug Deliv. Ther..

[20] Saraf A., Srinivas K.S., Chaturvedi A. (2016). Phytochemical and Elemental Profile of Embelia ribes Burn. F. Res. J. Pharm. Biol. Chem. Sci..

[21] Jelinski D.E. (1997). On genes and geography: A landscape perspective on genetic variation in natural plant populations. Landsc. Urban Plan..

[22] Gitzendanner M.A., Soltis P.S. (2000). Patterns of genetic variation in rare and widespread plant congeners. Am. J. Bot..

[23] Levy E., Byrne M., Coates D., Macdonald B., McArthur S., Van Leeuwen S. (2016). Contrasting influences of geographic range and distribution of populations on patterns of genetic diversity in two sympatric Pilbara acacias. PLoS ONE.

[24] Szczecińska M., Sramko G., Wołosz K., Sawicki J. (2016). Genetic diversity and population structure of the rare and endangered plant species *Pulsatilla patens (L.) Mill* in East Central Europe. PLoS ONE.

[25] Sasi R., Rajendran A. (2012). Diversity of wild fruits in Nilgiri Hills of the Southern Western Ghats-ethnobotanical aspects. Int. J. Appl. Biol. Pharm. Technol.

[26] Oritani Y., Okitsu T., Nishimura E., Sai M., Ito T., Takeuchi S. (2016). Enhanced glucose tolerance by intravascularly administered piceatannol in freely moving healthy rats. Biochem. Biophys. Res. Commun..

[27] Zhang A.J., Rimando A.M., Mizuno C.S., Mathews S.T. (2017). α-Glucosidase inhibitory effect of resveratrol and piceatannol. J. Nutr. Biochem..

[28] Gupta S., Sanyal S., Kanwar U. (1989). Antispermatogenic effect of embelin, a plant benzoquinone, on male albino rats in vivo and in vitro. Contraception.

[29] Ogbuewu I.P., Unamba-Oparah I.C., Odoemenam V.U., Etuk I.F., Okoli I.C. (2011). The potentiality of medicinal plants as the source of new contraceptive principles in males. North Am. J. Med. Sci..

[30] Etta H.E., Bassey U.P., Eneobong E.E., Okon O.B. (2009). Anti-spermatogenic effects of ethanol extract of Mucuna urens. J. Reprod. Contracept..

[31] Doyle J.J., Doyle J.L. (1990). Isolation of plant DNA from fresh tissue. Focus.

[32] Khan S., Qureshi M.I., Alam T., Abdin M. (2007). Protocol for isolation of genomic DNA from dry and fresh roots of medicinal plants suitable for RAPD and restriction digestion. Afr. J. Biotechnol..

[33] Hammer Ø., Harper D.A., Ryan P.D. (2001). PAST: Paleontological statistics software package for education and data analysis. Palaeontol. Electron..

[34] Perrier X., Jacquemoud-Collet J.-P. (2015). DARwin software. 2006. https://darwin.cirad.fr/.

