# Supplementary material for: Assessing the Impact of Geographical Distribution and Genetic Diversity on Metabolic Profiles of a Medicinal Plant, Embelia ribes Burm. f
Source: Plants (Basel). 2022 Oct 27;11(21):2861. doi: 10.3390/plants11212861 (PMC9653573; doi:10.3390/plants11212861)

Supplemental Figures

Figure S1: Representative LC-MS chromatograms (BPC) from fruits samples from all accessions. Representative chromatograms are showing in (A) negative and (B) positive mode.

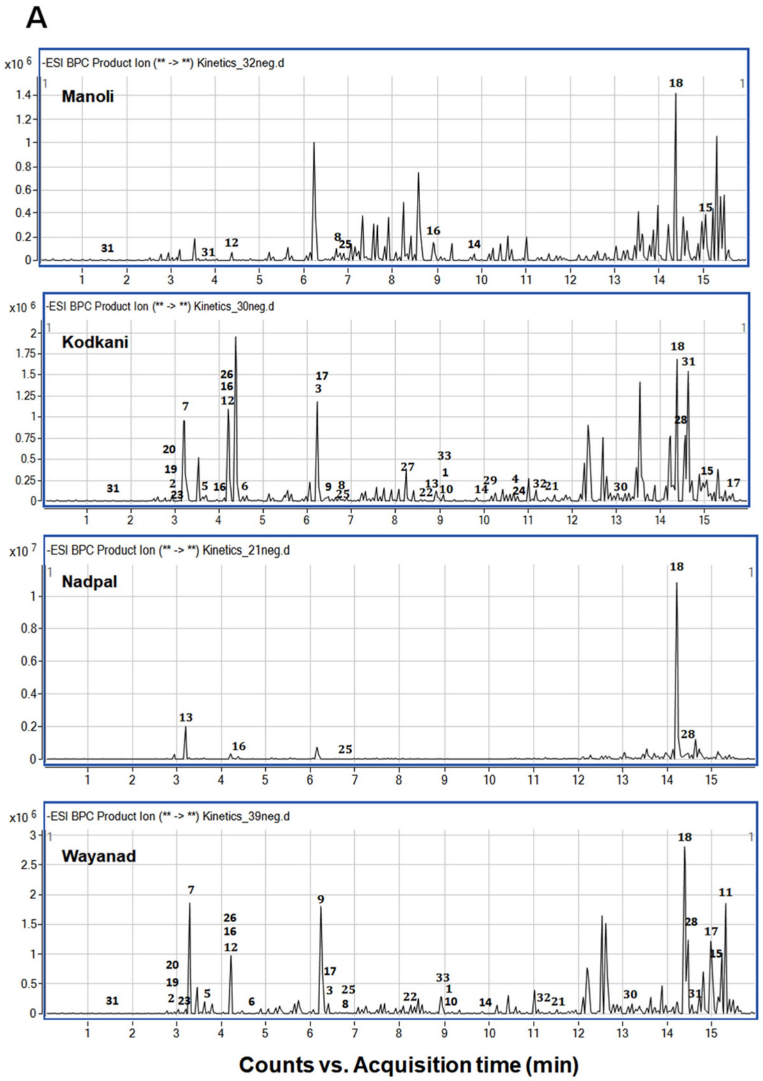

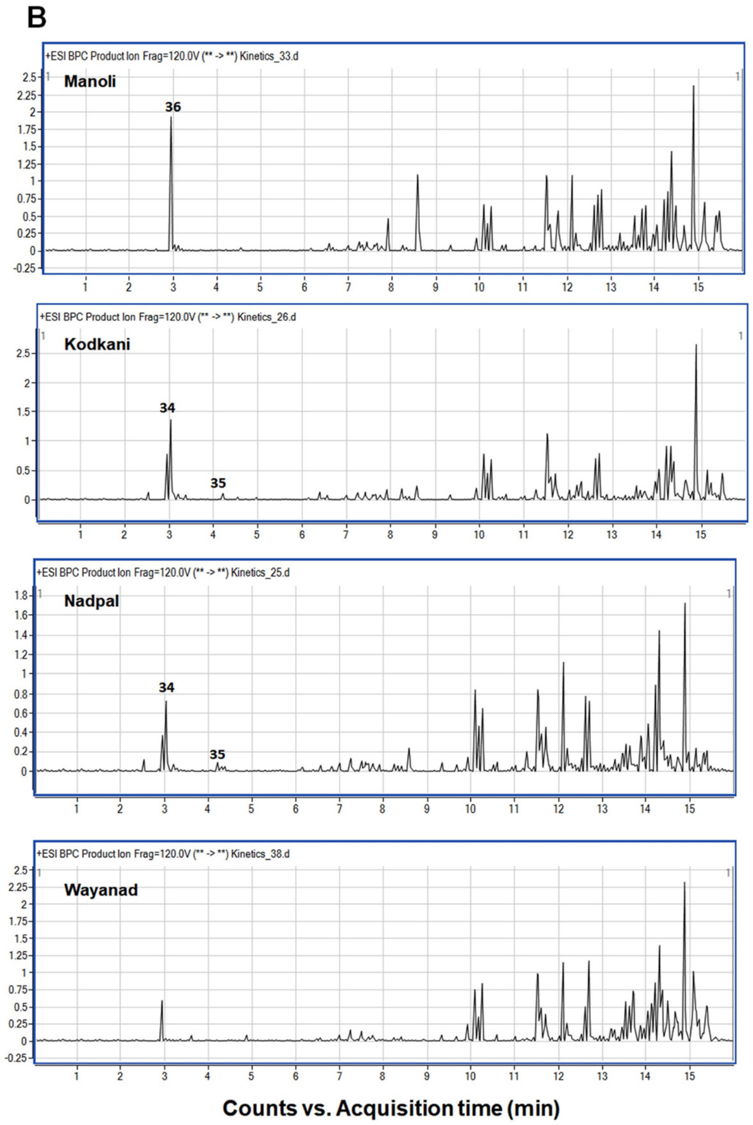

Figure S2: MS and MS-MS spectra for 36 identified compounds from different accessions of Embelia fruits.

1. **$C_6H_4O_2$, m/z-153.0197, 1, 2-Benzoquinone**

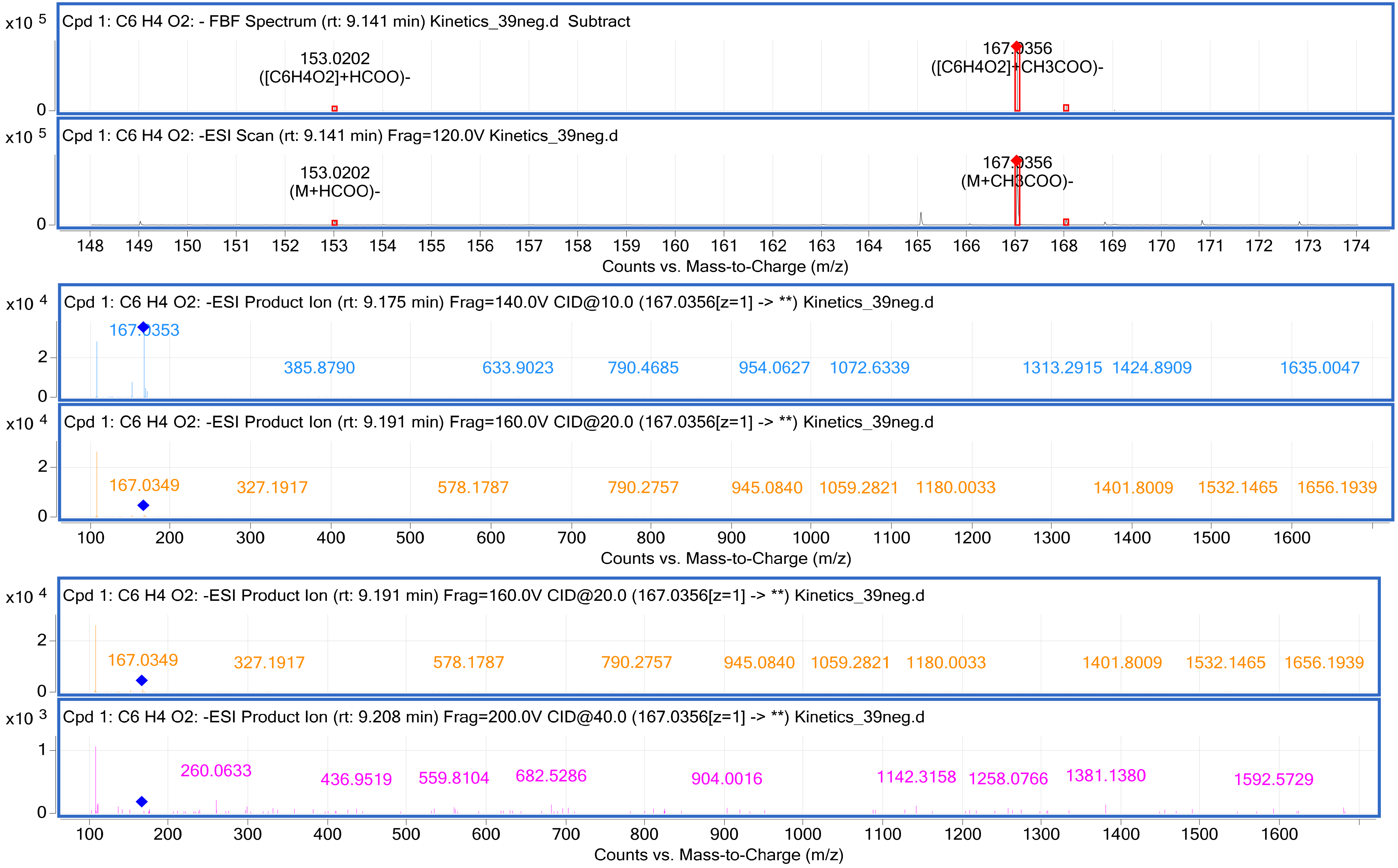

2. $C_5H_{12}S_2$, m/z - 135.0307, 1-pentanesulfenothoic acid

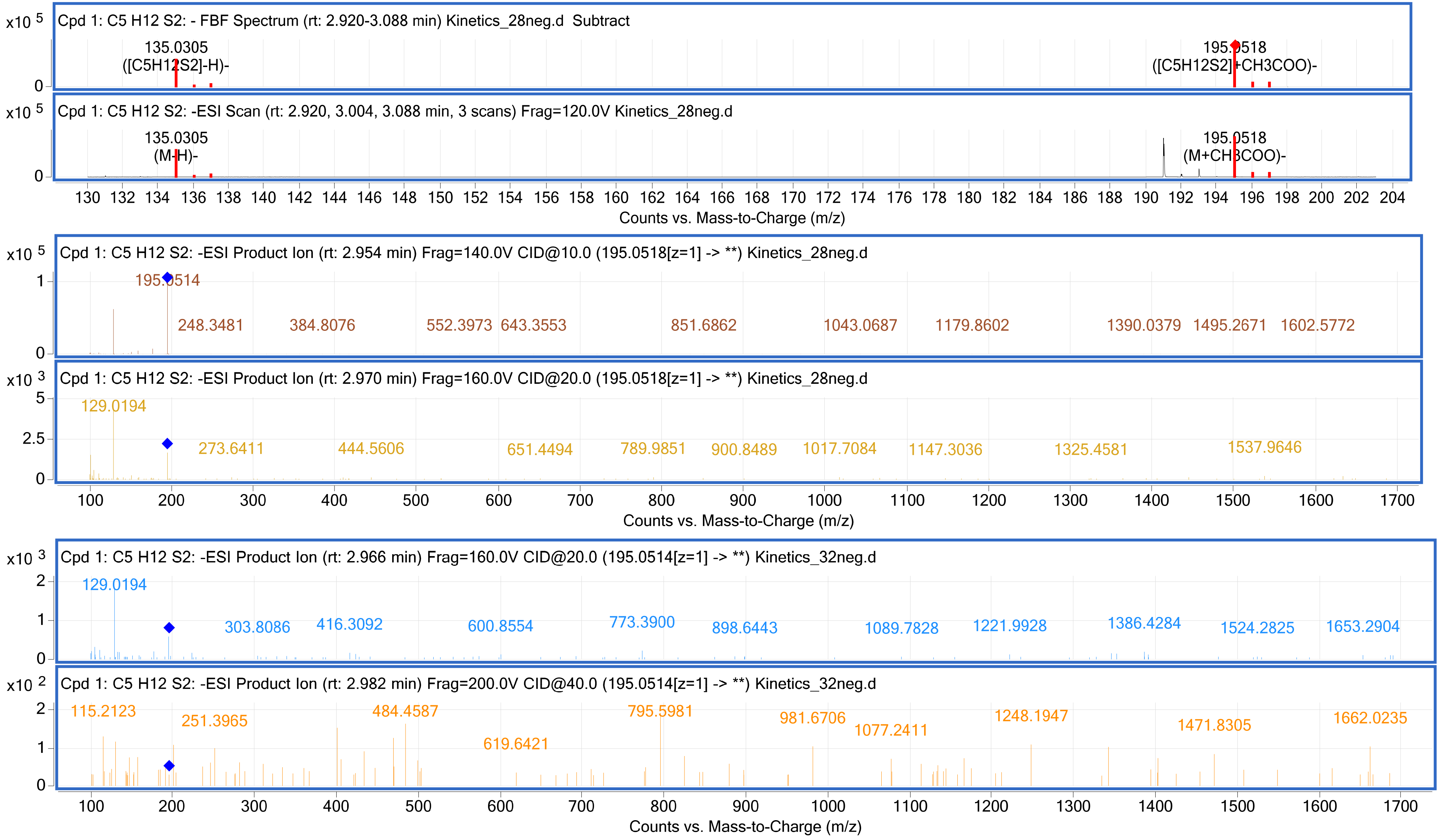

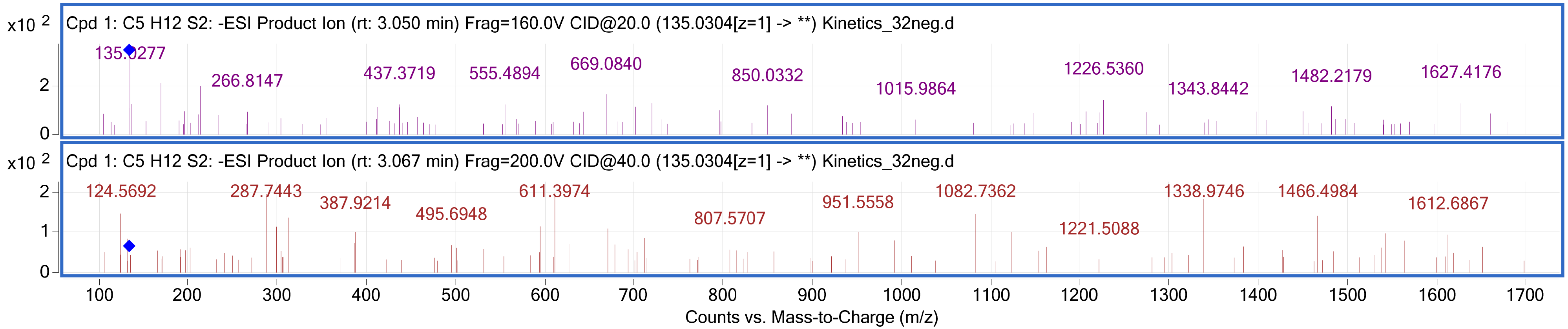

3. C_6 H_8 O_5, m/z- 205.0361, 2-Formylglutarate

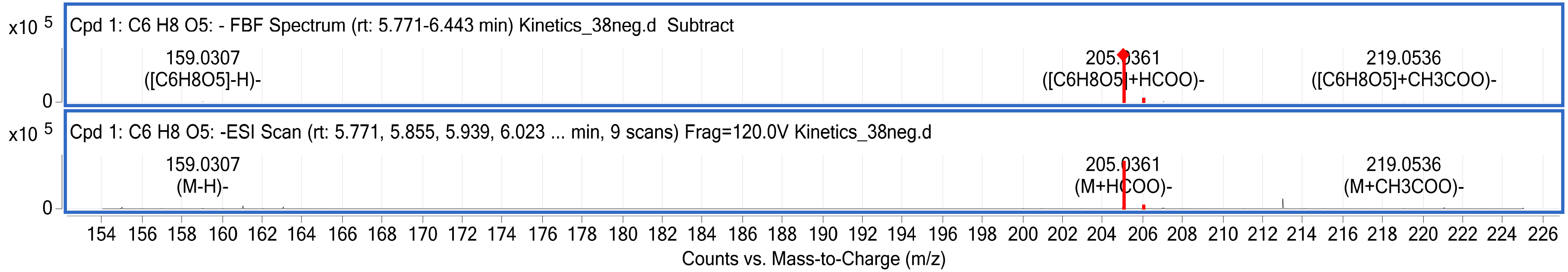

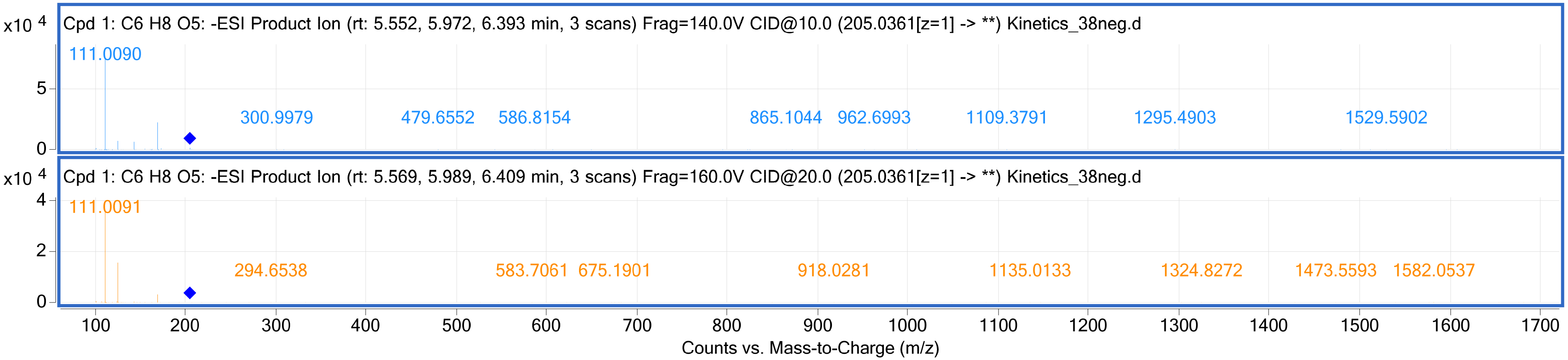

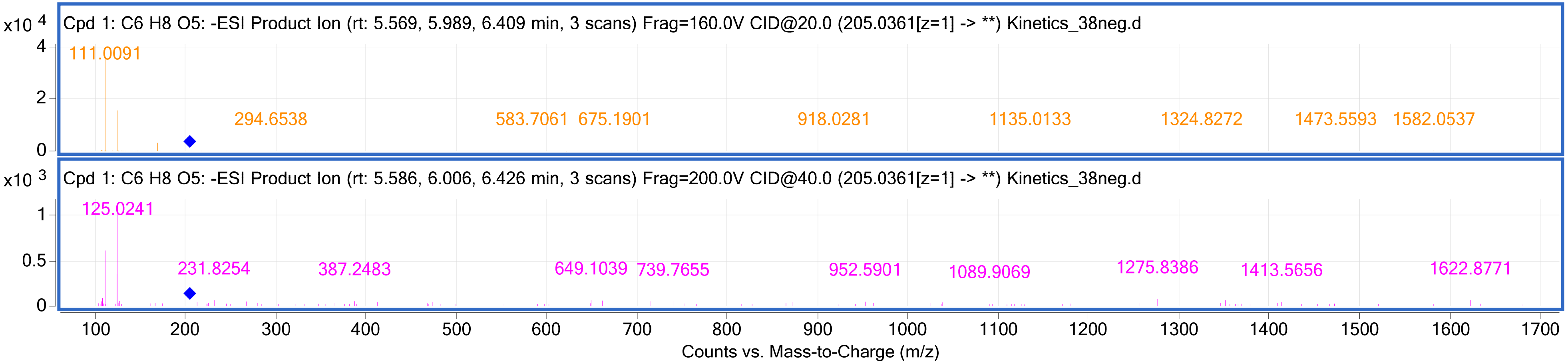

4. **$C_{15}H_{12}O_5$, m/z- 271.0626, 3,4,5-Trihydroxyflavanone**

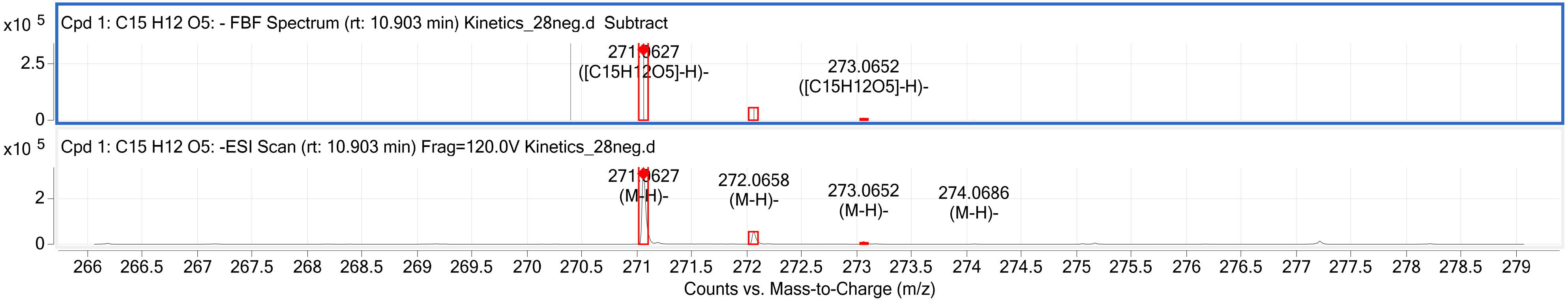

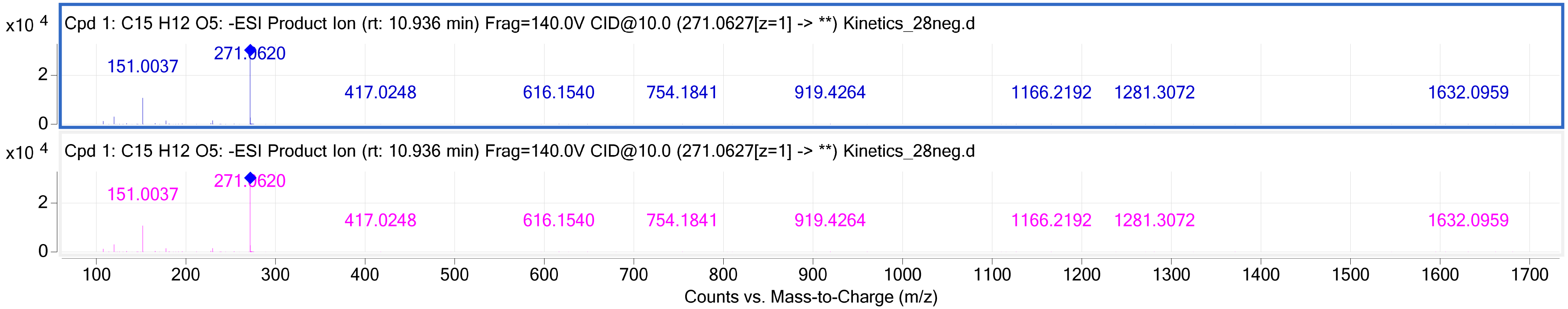

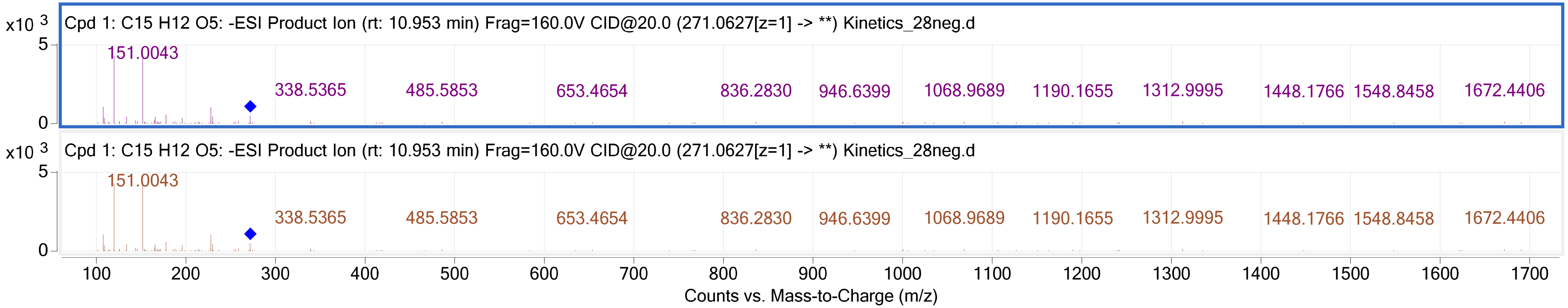

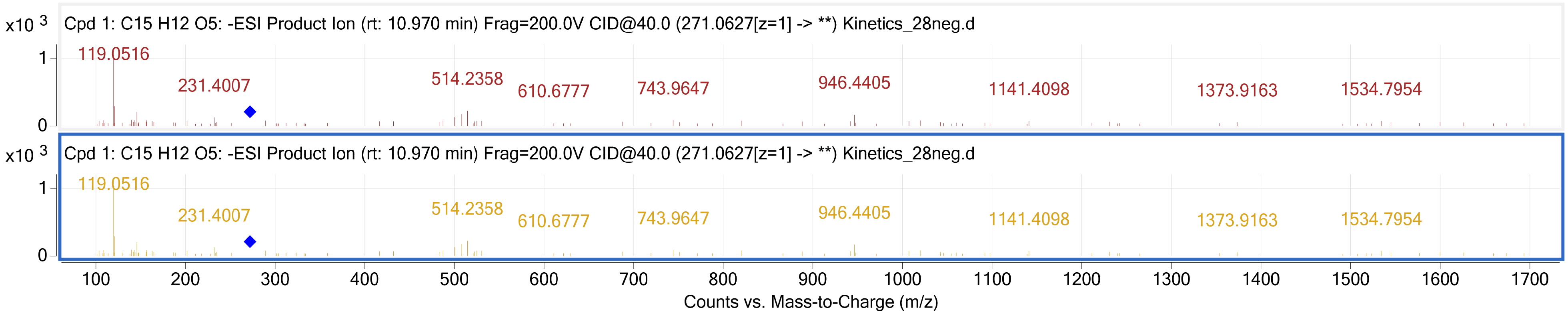

5. $C_4H_8O_4$, m/z- 179.0566, mass-120.0432, 3, 4-Dihydroxybutyric acid

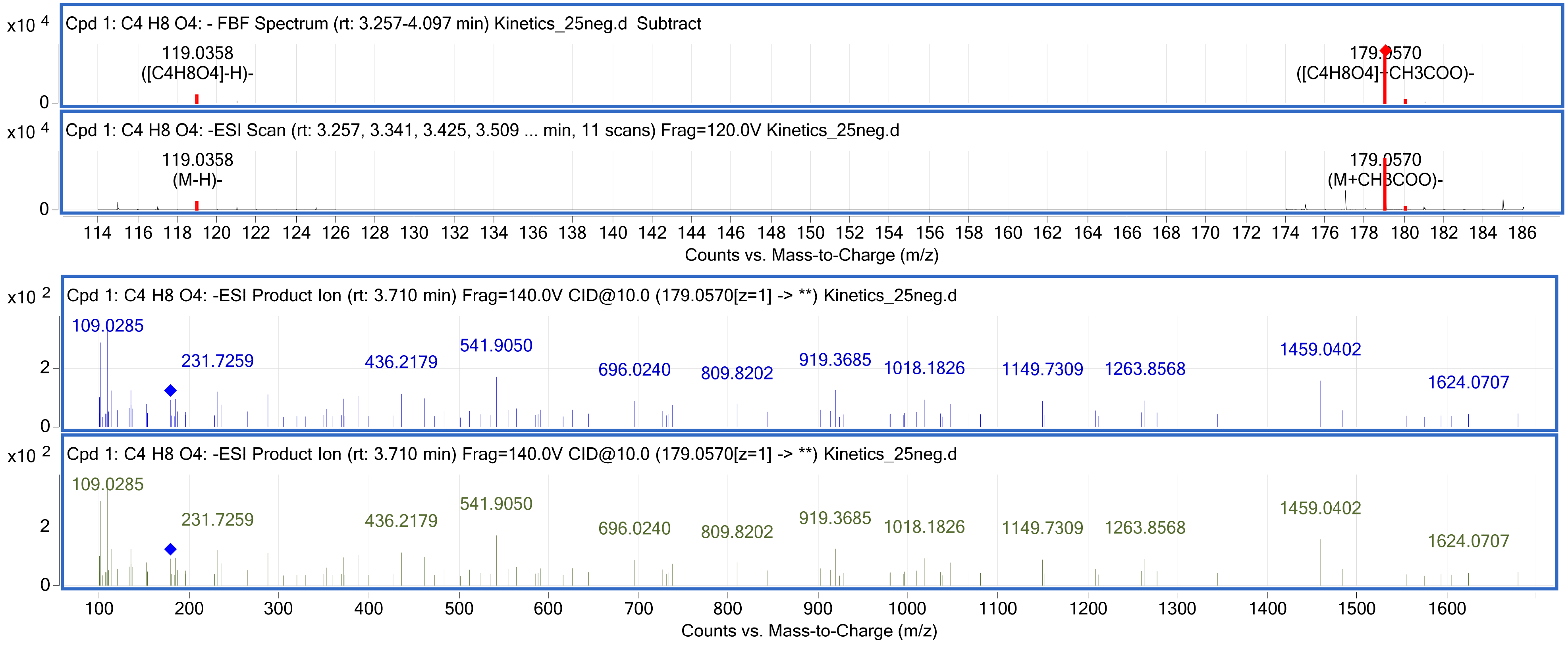

x10 4
Cpd 1: C4 H8 O4: - FBF Spectrum (rt: 3.257-4.097 min) Kinetics_25neg.d Subtract
119.0358
([C4H8O4]-H)-
179.0570
([C4H8O4]+CH3COO)-
0
x10 4
Cpd 1: C4 H8 O4: -ESI Scan (rt: 3.257, 3.341, 3.425, 3.509 ... min, 11 scans) Frag=120.0V Kinetics_25neg.d
119.0358
(M-H)-
179.0570
(M+CH3COO)-
0
114 116 118 120 122 124 126 128 130 132 134 136 138 140 142 144 146 148 150 152 154 156 158 160 162 164 166 168 170 172 174 176 178 180 182 184 186
Counts vs. Mass-to-Charge (m/z)
x10 2
Cpd 1: C4 H8 O4: -ESI Product Ion (rt: 3.710 min) Frag=140.0V CID@10.0 (179.0570[z=1] -> **) Kinetics_25neg.d
109.0285
231.7259
436.2179
541.9050
696.0240
809.8202
919.3685
1018.1826
1149.7309
1263.8568
1459.0402
1624.0707
2
0
x10 2
Cpd 1: C4 H8 O4: -ESI Product Ion (rt: 3.710 min) Frag=140.0V CID@10.0 (179.0570[z=1] -> **) Kinetics_25neg.d
109.0285
231.7259
436.2179
541.9050
696.0240
809.8202
919.3685
1018.1826
1149.7309
1263.8568
1459.0402
1624.0707
2
0
100 200 300 400 500 600 700 800 900 1000 1100 1200 1300 1400 1500 1600
Counts vs. Mass-to-Charge (m/z)

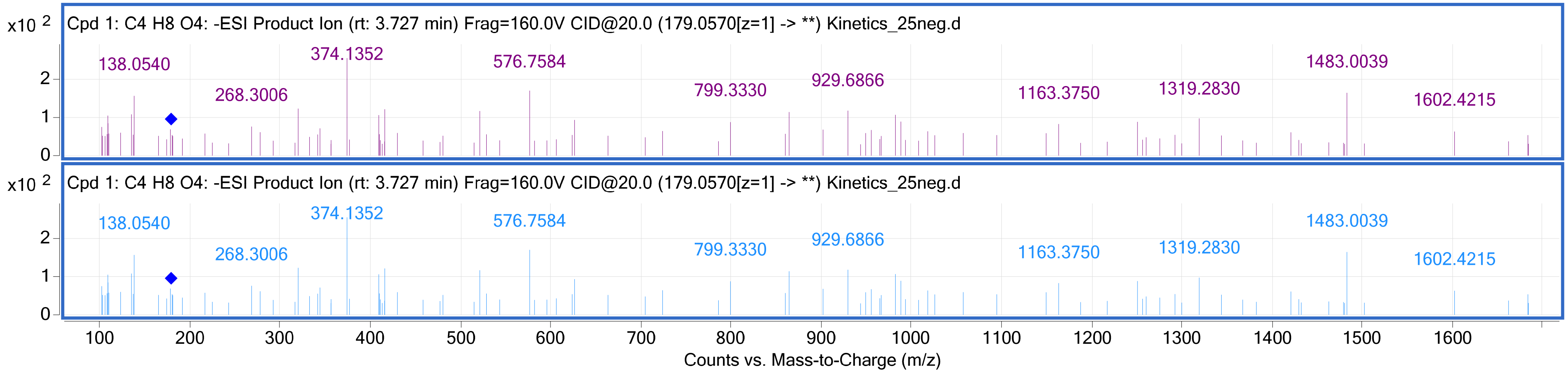

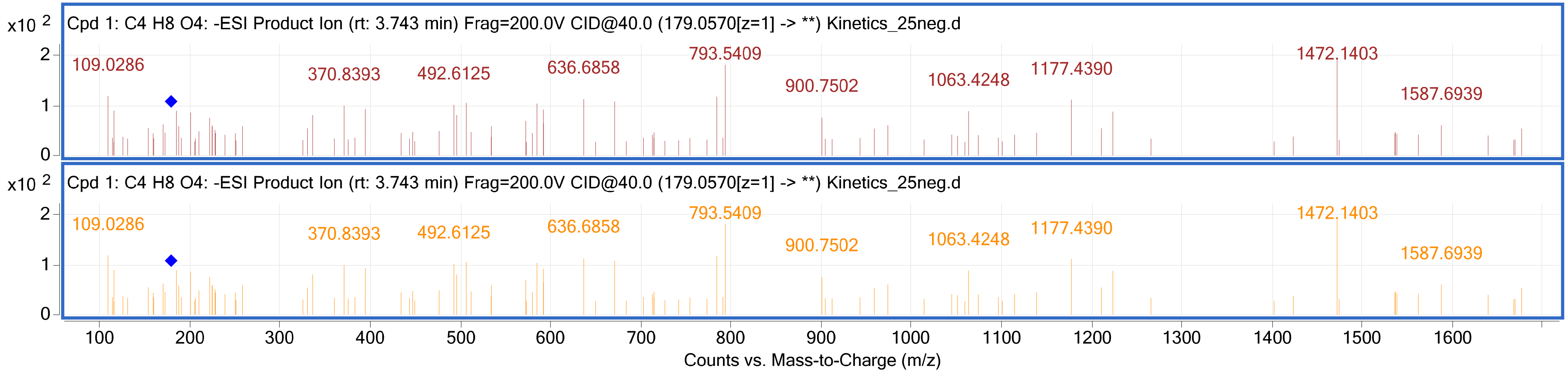

6. **$C_9H_{11}NO_3$, m/z- 180.0672, 3-Amino-3-(4-hydroxyphenyl) propanoate**

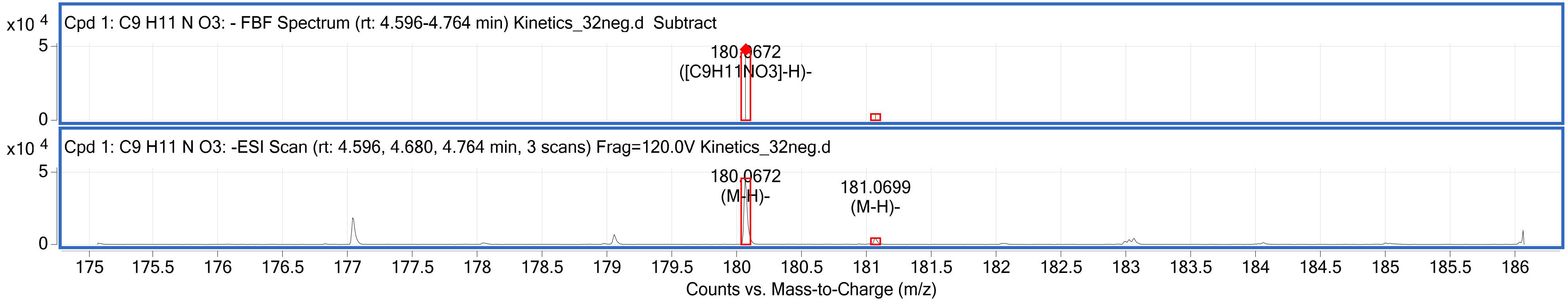

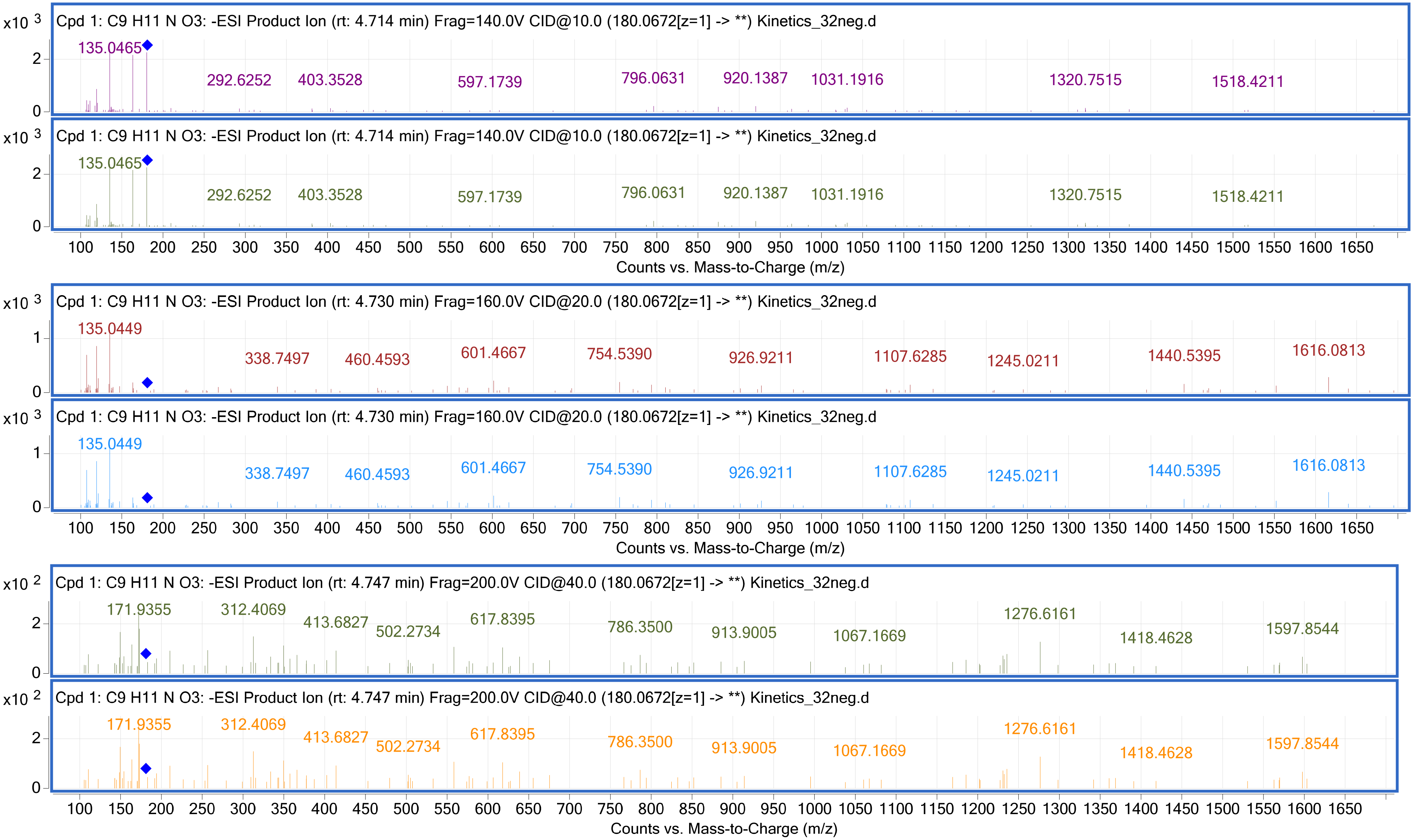
x10 3
Cpd 1: C9 H11 N O3: -ESI Product Ion (rt: 4.714 min) Frag=140.0V CID@10.0 (180.0672[z=1] -> **) Kinetics_32neg.d
135.0465
292.6252
403.3528
597.1739
796.0631
920.1387
1031.1916
1320.7515
1518.4211
x10 3
Cpd 1: C9 H11 N O3: -ESI Product Ion (rt: 4.714 min) Frag=140.0V CID@10.0 (180.0672[z=1] -> **) Kinetics_32neg.d
135.0465
292.6252
403.3528
597.1739
796.0631
920.1387
1031.1916
1320.7515
1518.4211
Counts vs. Mass-to-Charge (m/z)
x10 3
Cpd 1: C9 H11 N O3: -ESI Product Ion (rt: 4.730 min) Frag=160.0V CID@20.0 (180.0672[z=1] -> **) Kinetics_32neg.d
135.0449
338.7497
460.4593
601.4667
754.5390
926.9211
1107.6285
1245.0211
1440.5395
1616.0813
x10 3
Cpd 1: C9 H11 N O3: -ESI Product Ion (rt: 4.730 min) Frag=160.0V CID@20.0 (180.0672[z=1] -> **) Kinetics_32neg.d
135.0449
338.7497
460.4593
601.4667
754.5390
926.9211
1107.6285
1245.0211
1440.5395
1616.0813
Counts vs. Mass-to-Charge (m/z)
x10 2
Cpd 1: C9 H11 N O3: -ESI Product Ion (rt: 4.747 min) Frag=200.0V CID@40.0 (180.0672[z=1] -> **) Kinetics_32neg.d
171.9355
312.4069
413.6827
502.2734
617.8395
786.3500
913.9005
1067.1669
1276.6161
1418.4628
1597.8544
x10 2
Cpd 1: C9 H11 N O3: -ESI Product Ion (rt: 4.747 min) Frag=200.0V CID@40.0 (180.0672[z=1] -> **) Kinetics_32neg.d
171.9355
312.4069
413.6827
502.2734
617.8395
786.3500
913.9005
1067.1669
1276.6161
1418.4628
1597.8544
Counts vs. Mass-to-Charge (m/z)

7. $C_5H_4O_3$, m/s- 111.0093, 3-Furoic acid

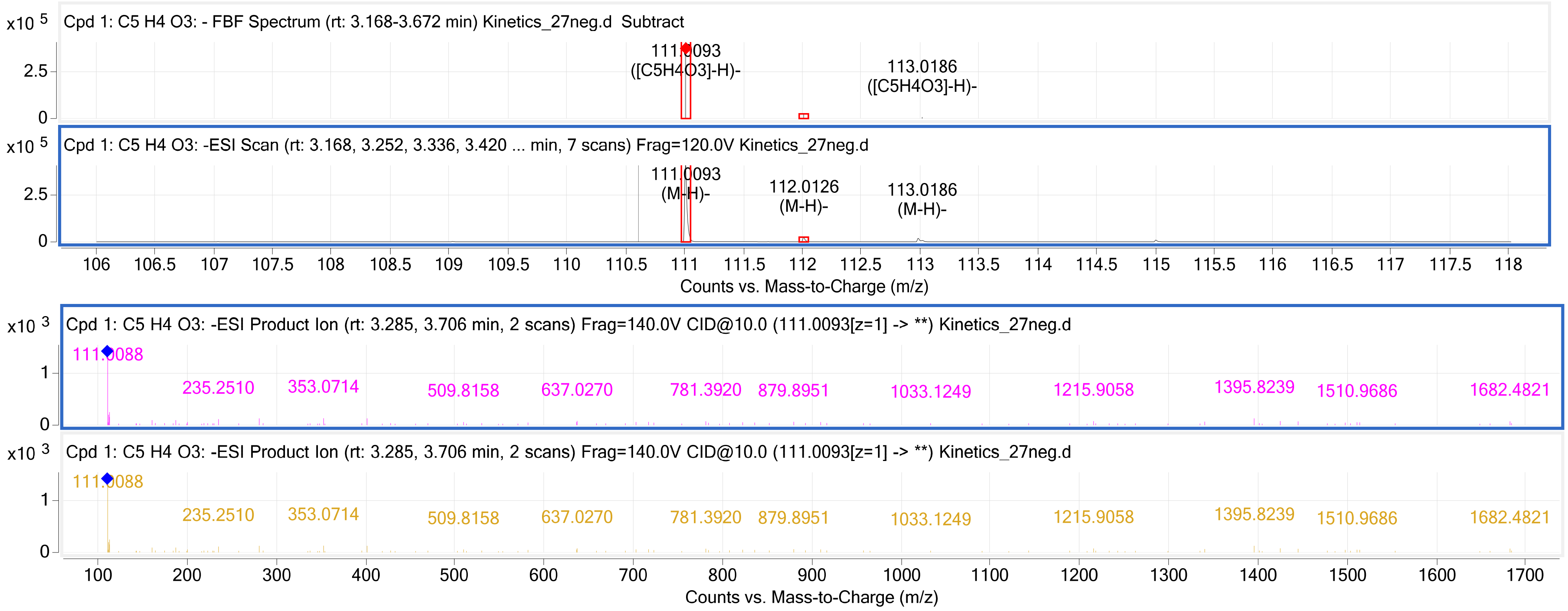

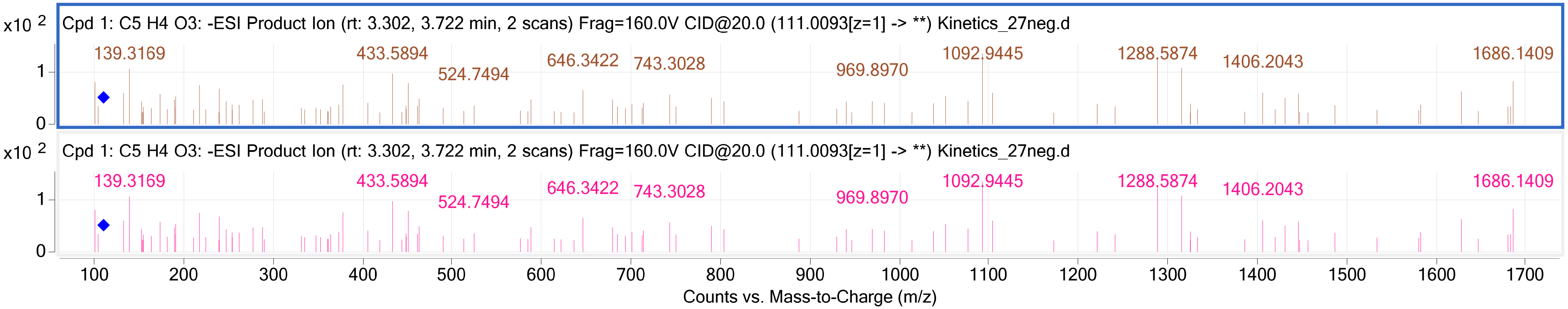

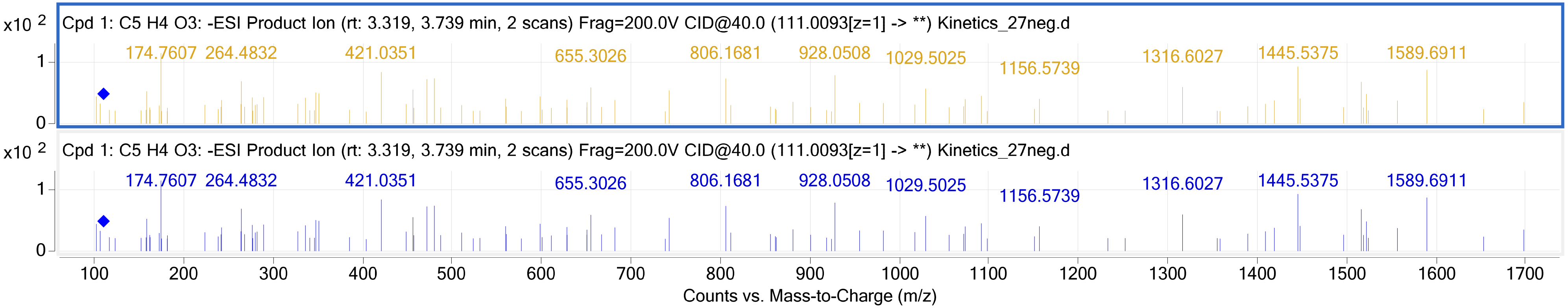

8. **$C_7H_{12}O_5$, m/z-221.0675, 3-Isopropylmalic acid**

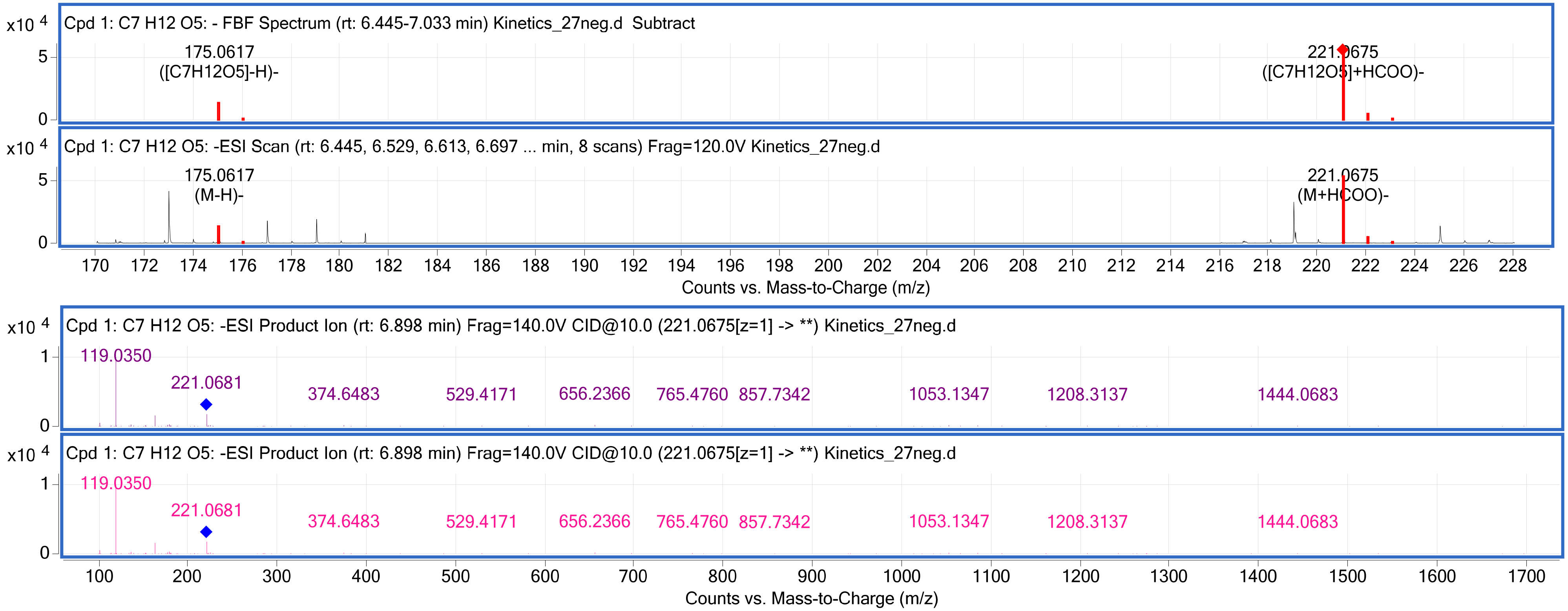
x10 4
Cpd 1: C7 H12 O5: - FBF Spectrum (rt: 6.445-7.033 min) Kinetics_27neg.d Subtract
175.0617
([C7H12O5]-H)-
221.0675
([C7H12O5]+HCOO)-
Cpd 1: C7 H12 O5: -ESI Scan (rt: 6.445, 6.529, 6.613, 6.697 ... min, 8 scans) Frag=120.0V Kinetics_27neg.d
175.0617
(M-H)-
221.0675
(M+HCOO)-
Counts vs. Mass-to-Charge (m/z)
Cpd 1: C7 H12 O5: -ESI Product Ion (rt: 6.898 min) Frag=140.0V CID@10.0 (221.0675[z=1] -> **) Kinetics_27neg.d
119.0350
221.0681
374.6483
529.4171
656.2366
765.4760
857.7342
1053.1347
1208.3137
1444.0683
Cpd 1: C7 H12 O5: -ESI Product Ion (rt: 6.898 min) Frag=140.0V CID@10.0 (221.0675[z=1] -> **) Kinetics_27neg.d
119.0350
221.0681
374.6483
529.4171
656.2366
765.4760
857.7342
1053.1347
1208.3137
1444.0683
Counts vs. Mass-to-Charge (m/z)

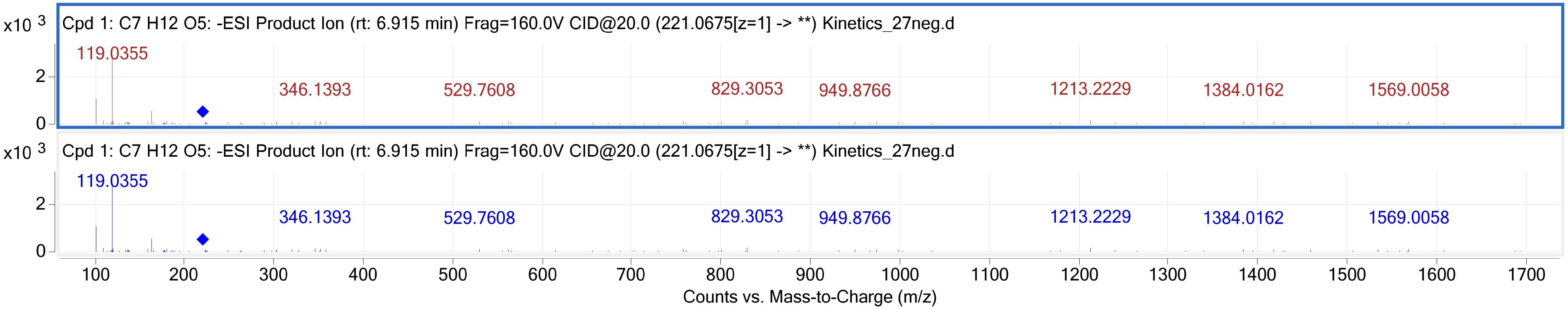

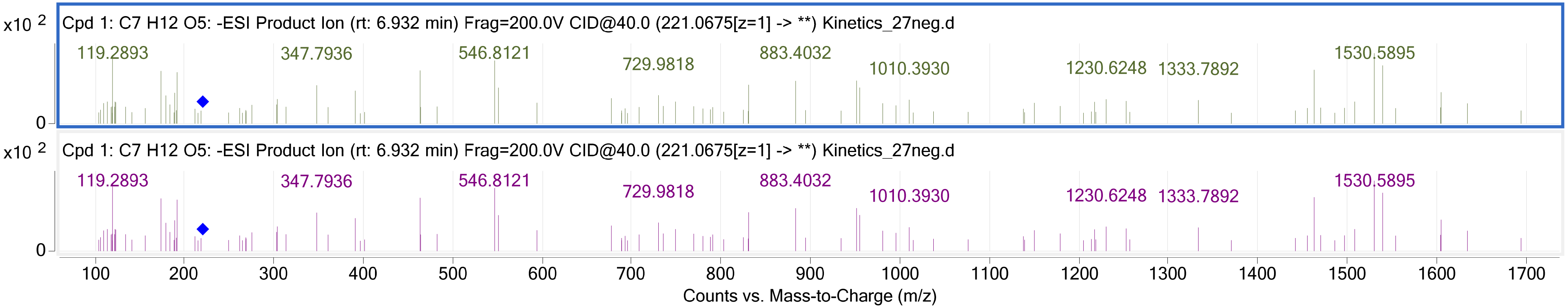

9. **$C_6H_6O_6$, m/z- 173.0097, Aconitic acid**

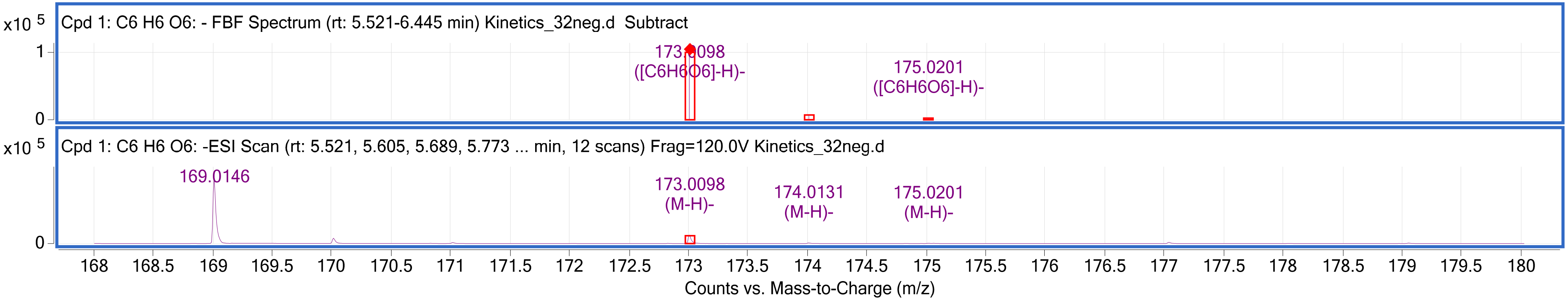

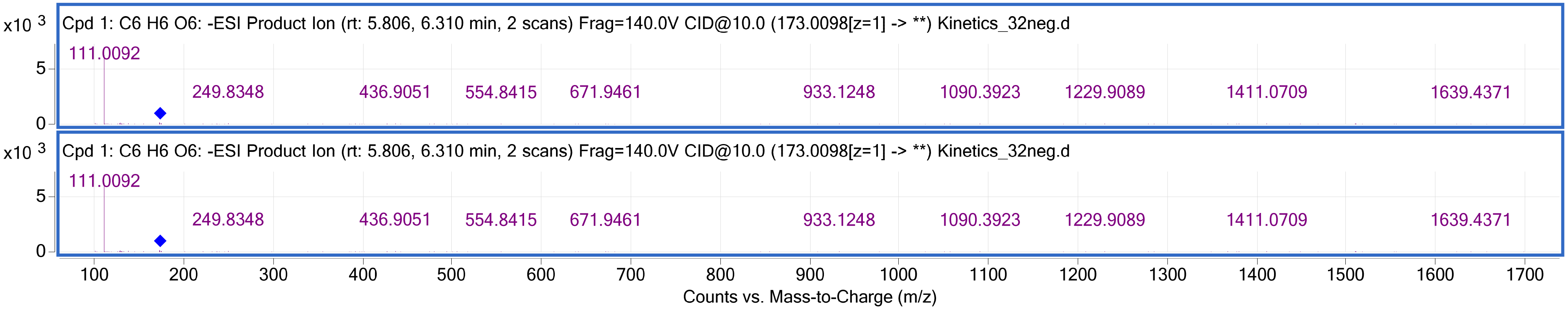

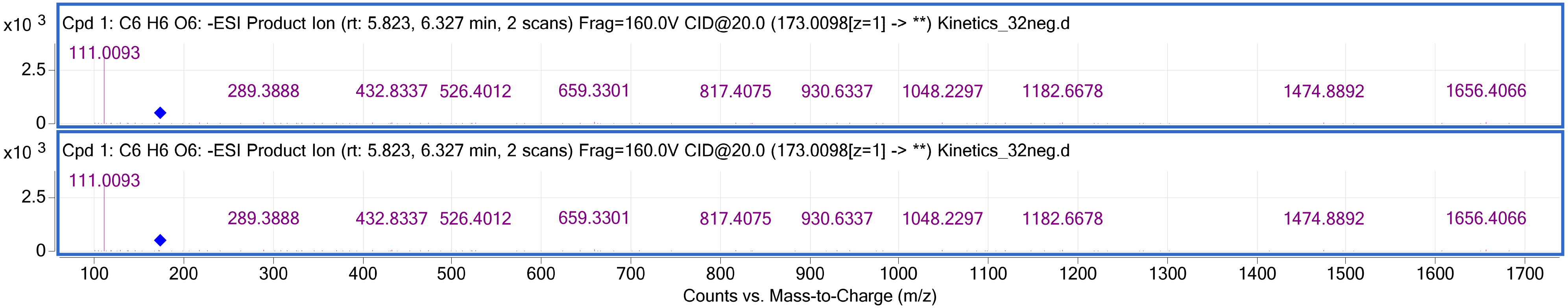

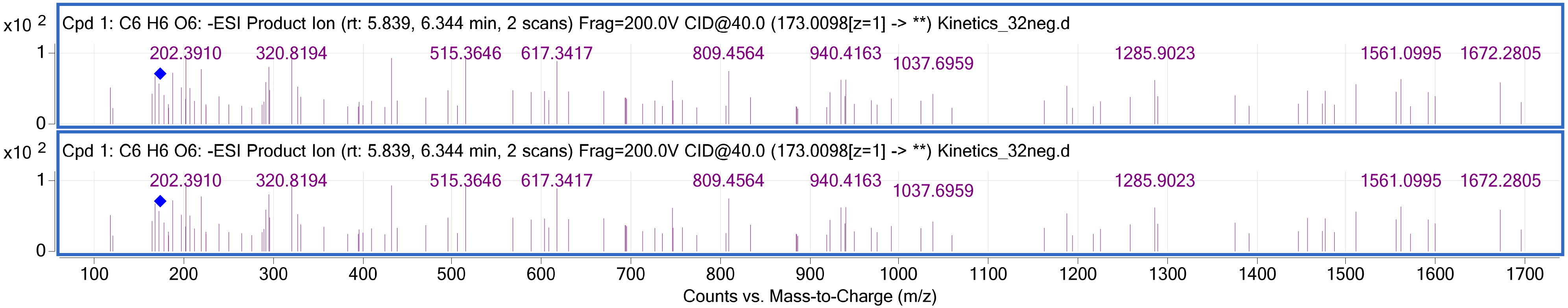

10. $C_7H_6O_2$, m/z-121.03, Benzoic acid

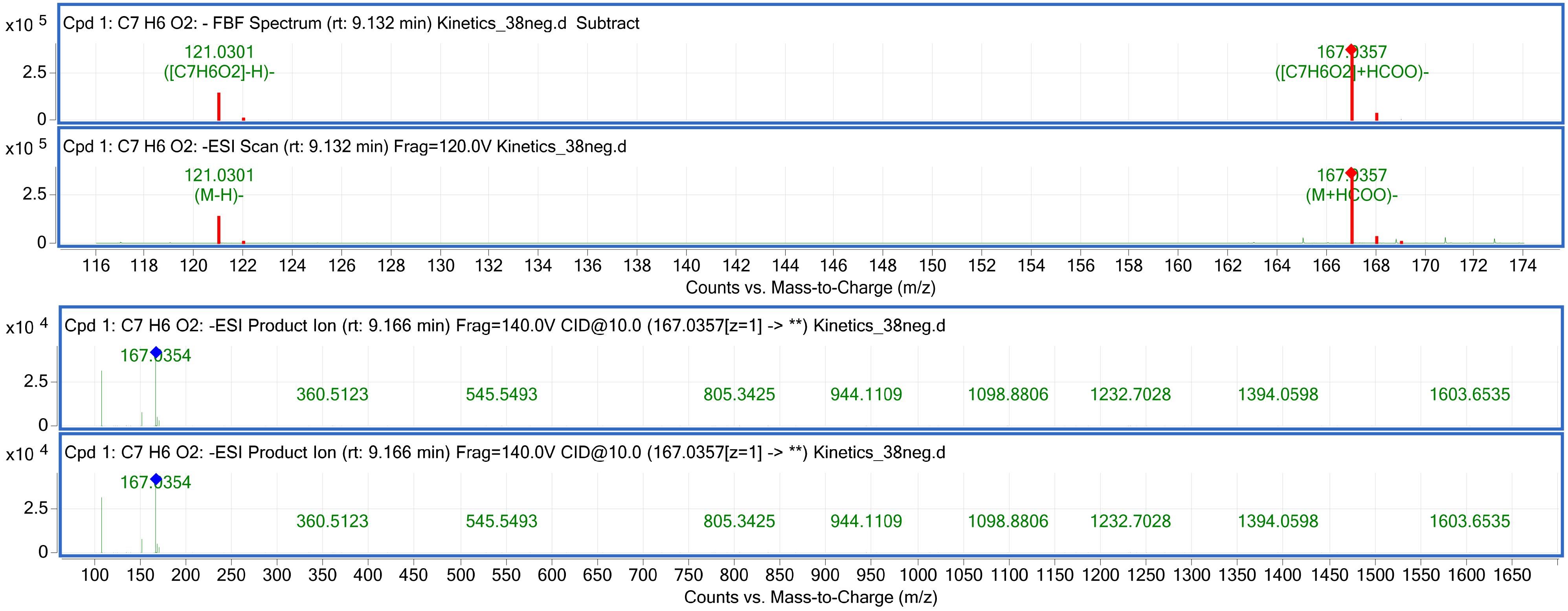

Cpd 1: C7 H6 O2: - FBF Spectrum (rt: 9.132 min) Kinetics_38neg.d Subtract
x10 5
121.0301
([C7H6O2]-H)-
167.0357
([C7H6O2]+HCOO)-
Cpd 1: C7 H6 O2: -ESI Scan (rt: 9.132 min) Frag=120.0V Kinetics_38neg.d
121.0301
(M-H)-
167.0357
(M+HCOO)-
Counts vs. Mass-to-Charge (m/z)
Cpd 1: C7 H6 O2: -ESI Product Ion (rt: 9.166 min) Frag=140.0V CID@10.0 (167.0357[z=1] -> **) Kinetics_38neg.d
x10 4
167.0354
360.5123
545.5493
805.3425
944.1109
1098.8806
1232.7028
1394.0598
1603.6535
Cpd 1: C7 H6 O2: -ESI Product Ion (rt: 9.166 min) Frag=140.0V CID@10.0 (167.0357[z=1] -> **) Kinetics_38neg.d
167.0354
360.5123
545.5493
805.3425
944.1109
1098.8806
1232.7028
1394.0598
1603.6535
Counts vs. Mass-to-Charge (m/z)

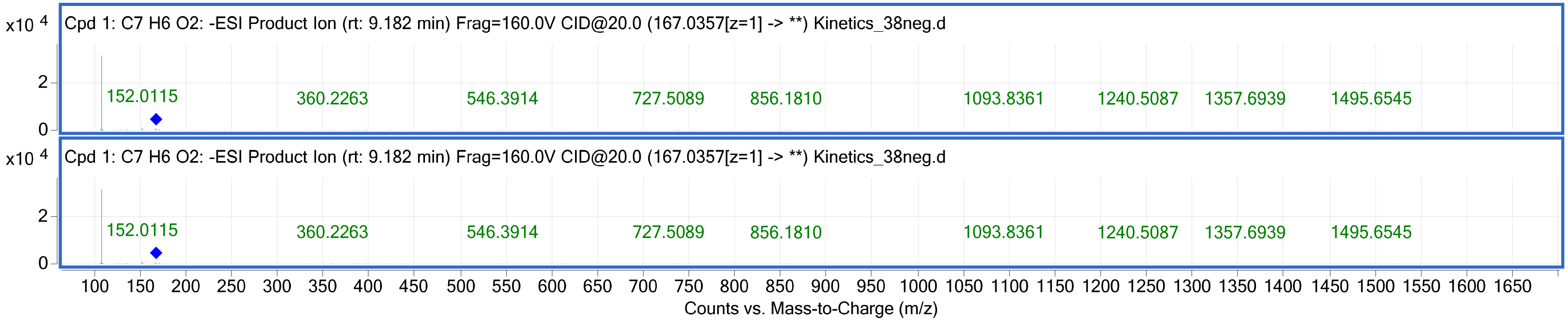

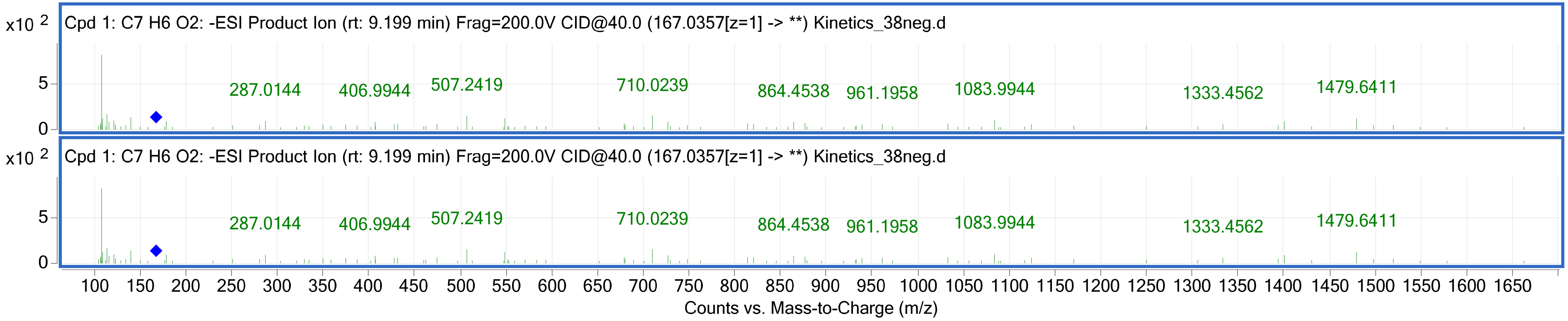

11. $C_{28}H_{40}O_4$, m/z-439.2854, Bolegrevilol

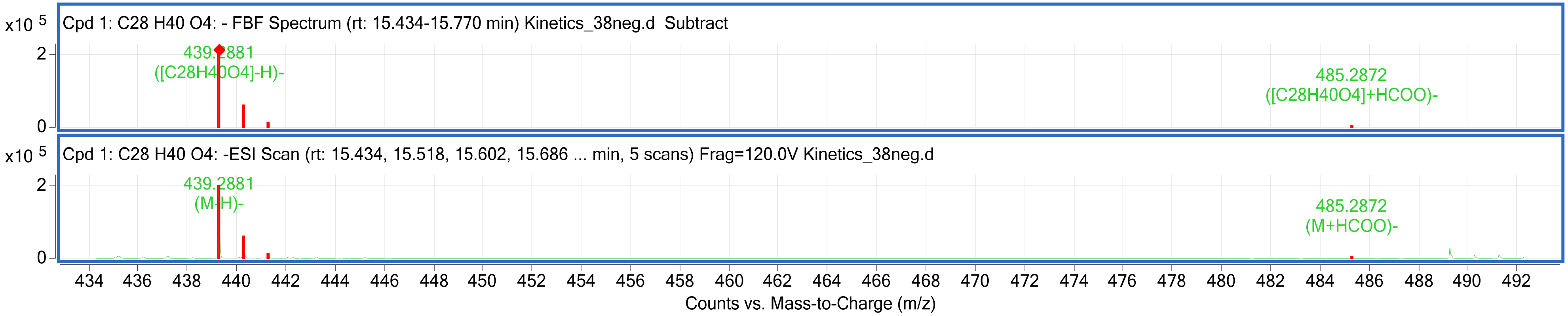

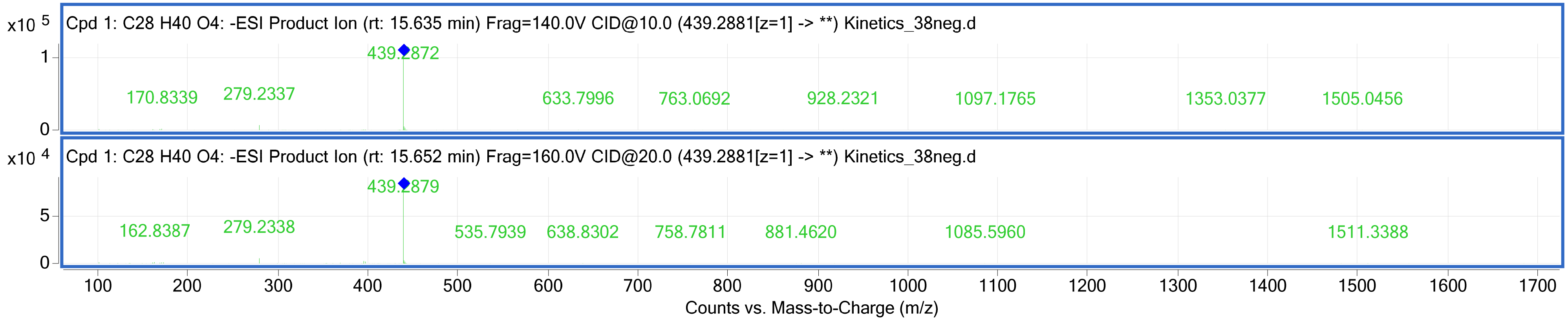

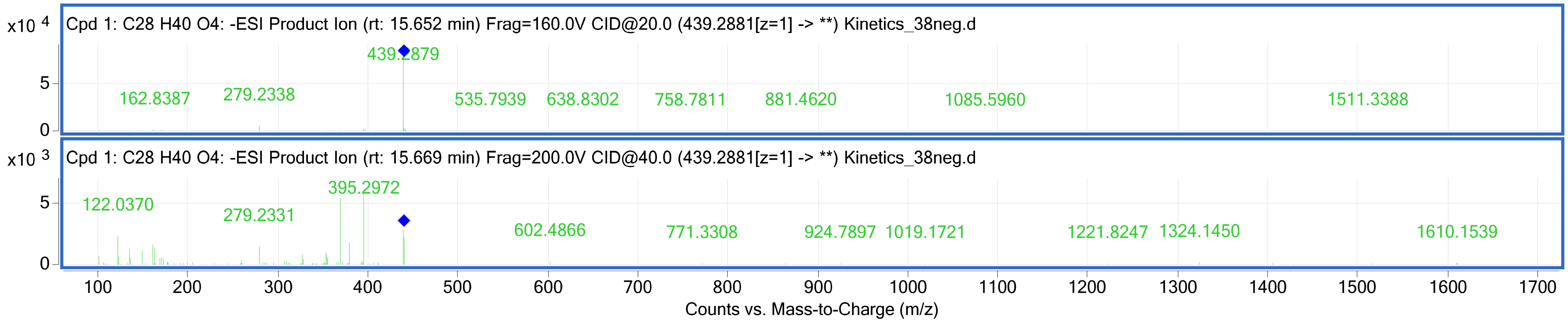

12. $C_6H_8O_7$, m/z-191.0201, Citric acid

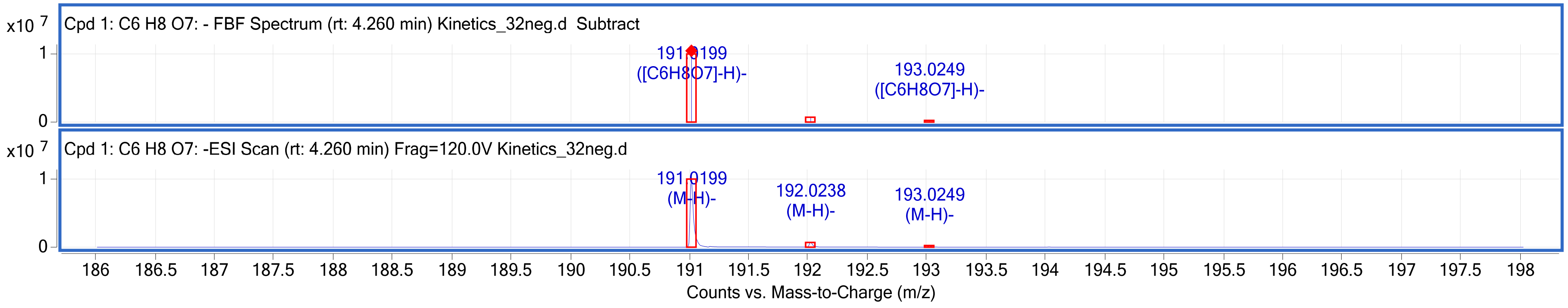

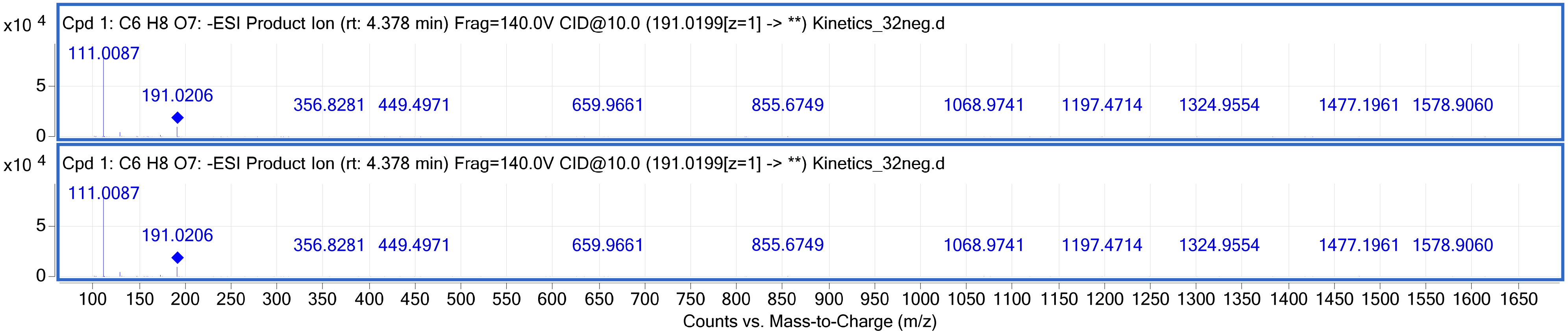
x10 4
Cpd 1: C6 H8 O7: -ESI Product Ion (rt: 4.378 min) Frag=140.0V CID@10.0 (191.0199[z=1] -> **) Kinetics_32neg.d
111.0087
191.0206
356.8281
449.4971
659.9661
855.6749
1068.9741
1197.4714
1324.9554
1477.1961
1578.9060
x10 4
Cpd 1: C6 H8 O7: -ESI Product Ion (rt: 4.378 min) Frag=140.0V CID@10.0 (191.0199[z=1] -> **) Kinetics_32neg.d
111.0087
191.0206
356.8281
449.4971
659.9661
855.6749
1068.9741
1197.4714
1324.9554
1477.1961
1578.9060
Counts vs. Mass-to-Charge (m/z)

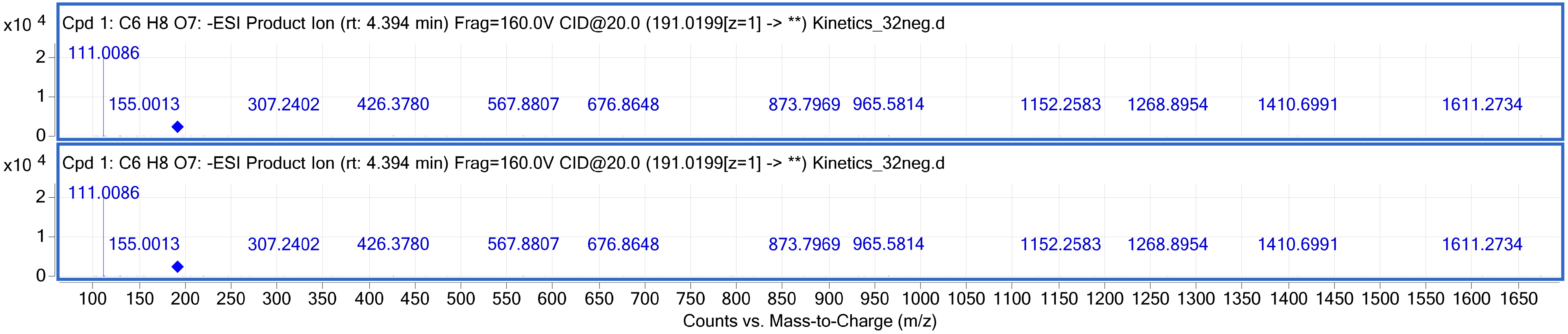
x10 4
Cpd 1: C6 H8 O7: -ESI Product Ion (rt: 4.394 min) Frag=160.0V CID@20.0 (191.0199[z=1] -> **) Kinetics_32neg.d
111.0086
155.0013
307.2402
426.3780
567.8807
676.8648
873.7969
965.5814
1152.2583
1268.8954
1410.6991
1611.2734
x10 4
Cpd 1: C6 H8 O7: -ESI Product Ion (rt: 4.394 min) Frag=160.0V CID@20.0 (191.0199[z=1] -> **) Kinetics_32neg.d
111.0086
155.0013
307.2402
426.3780
567.8807
676.8648
873.7969
965.5814
1152.2583
1268.8954
1410.6991
1611.2734
Counts vs. Mass-to-Charge (m/z)

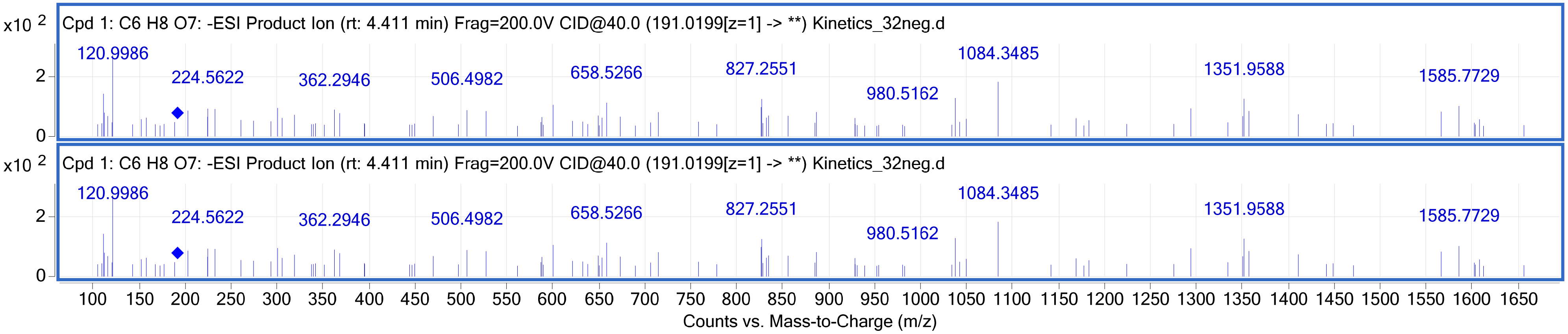
x10 2
Cpd 1: C6 H8 O7: -ESI Product Ion (rt: 4.411 min) Frag=200.0V CID@40.0 (191.0199[z=1] -> **) Kinetics_32neg.d
120.9986
224.5622
362.2946
506.4982
658.5266
827.2551
980.5162
1084.3485
1351.9588
1585.7729
x10 2
Cpd 1: C6 H8 O7: -ESI Product Ion (rt: 4.411 min) Frag=200.0V CID@40.0 (191.0199[z=1] -> **) Kinetics_32neg.d
120.9986
224.5622
362.2946
506.4982
658.5266
827.2551
980.5162
1084.3485
1351.9588
1585.7729
Counts vs. Mass-to-Charge (m/z)

13. $C_9H_8O_3$, m/z-163.04, Coumaric acid

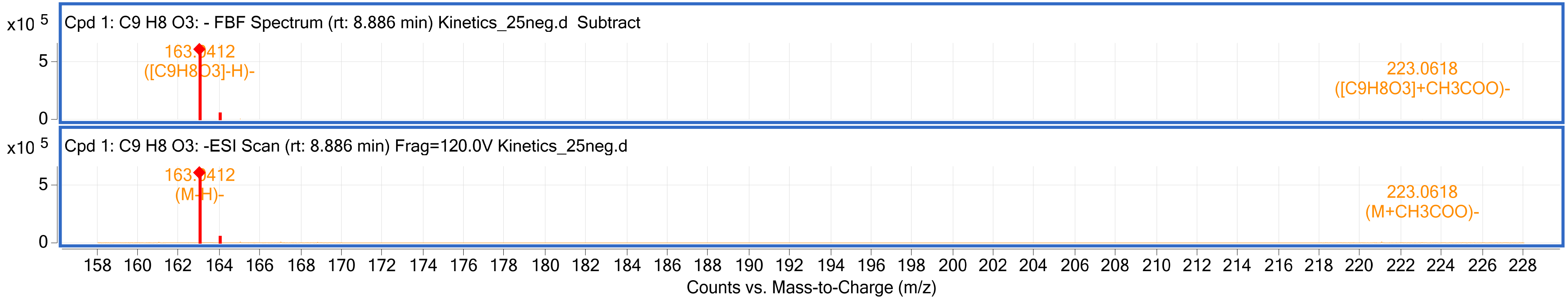

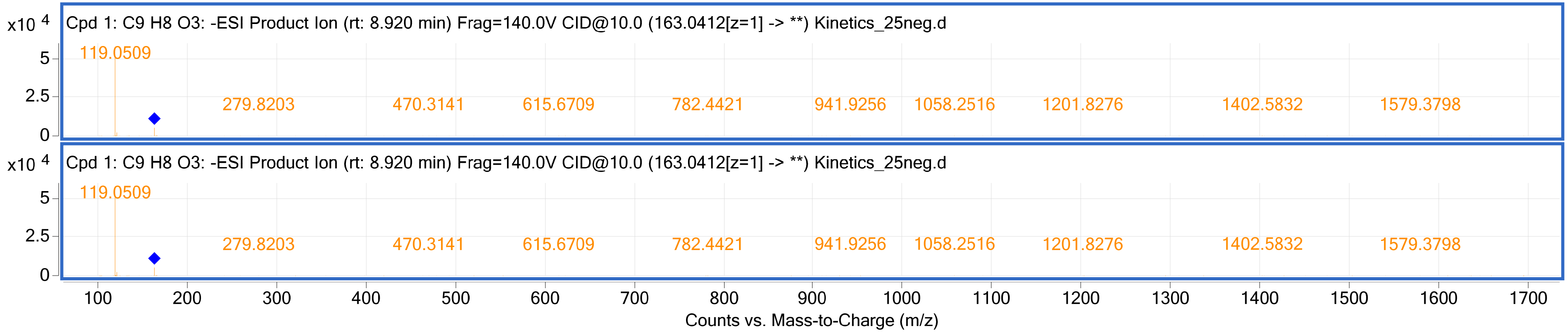

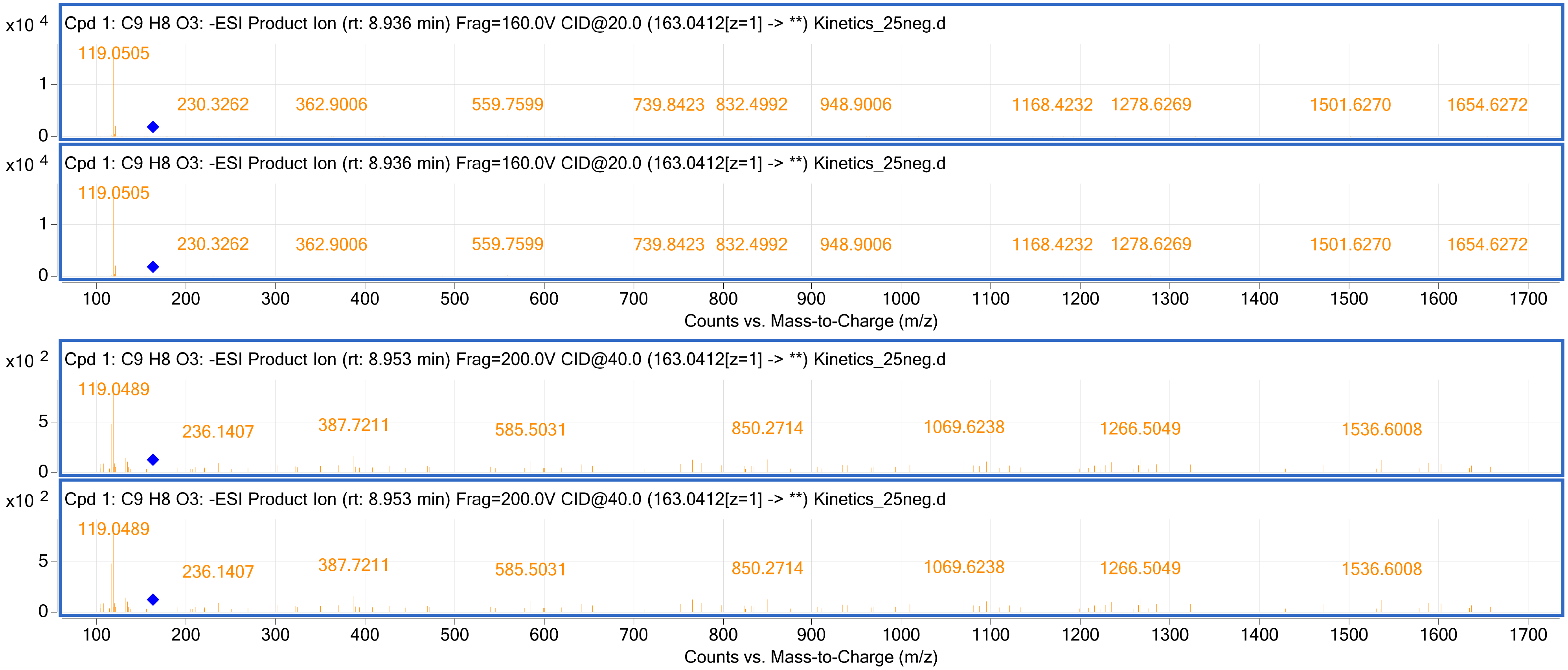

14. $C_{10}H_{18}O_4$, m/z-201.1143, Decanedioic acid

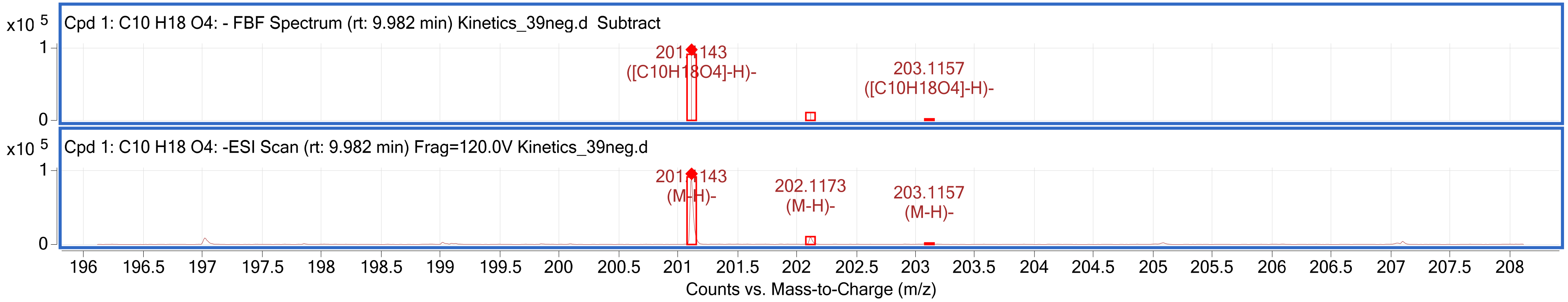

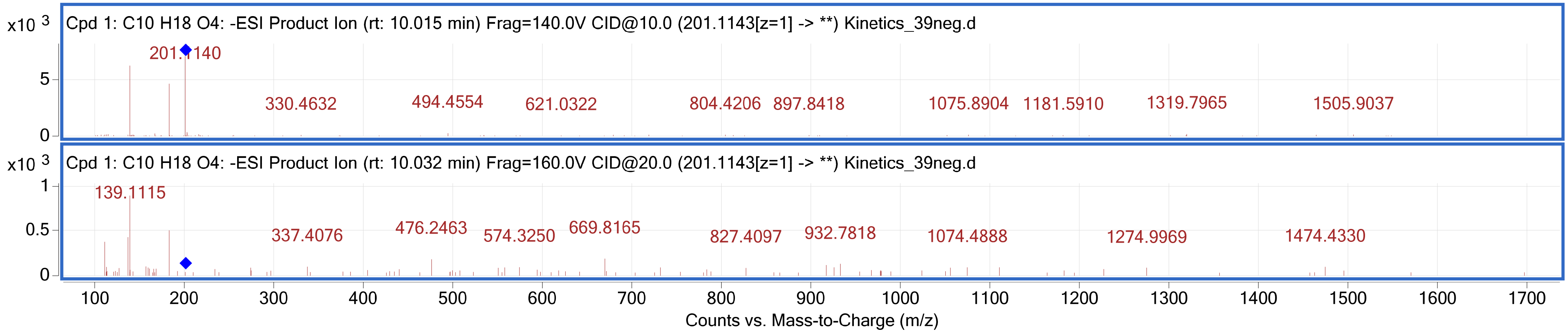

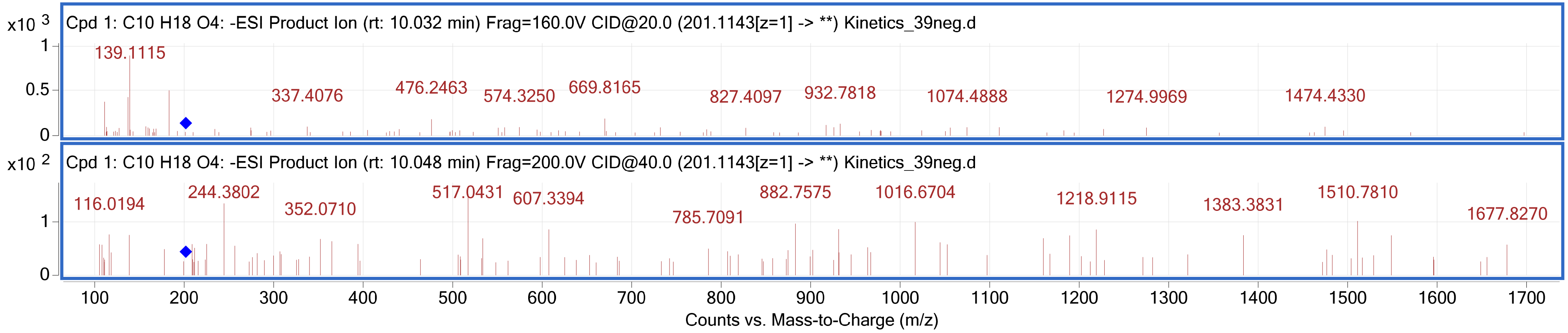

15. $C_{13}H_{24}O_2$, m/z-271.1918, Delta-tridecalactone

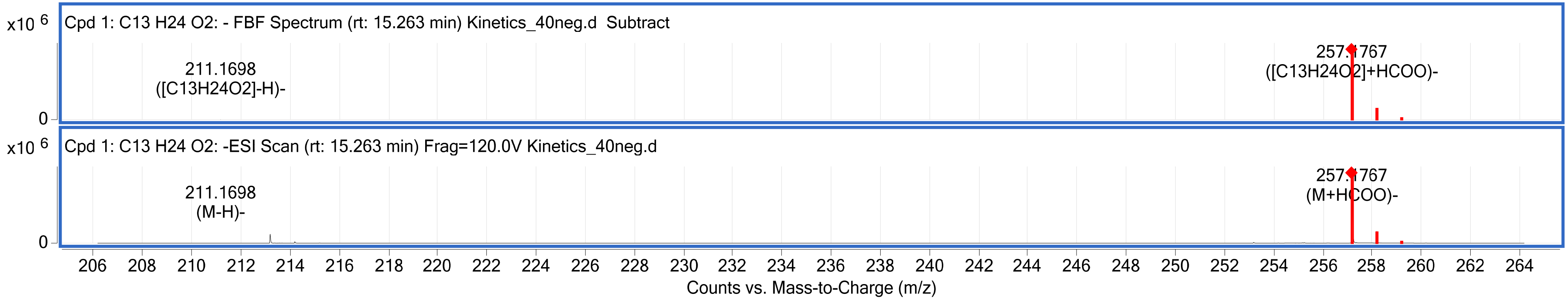

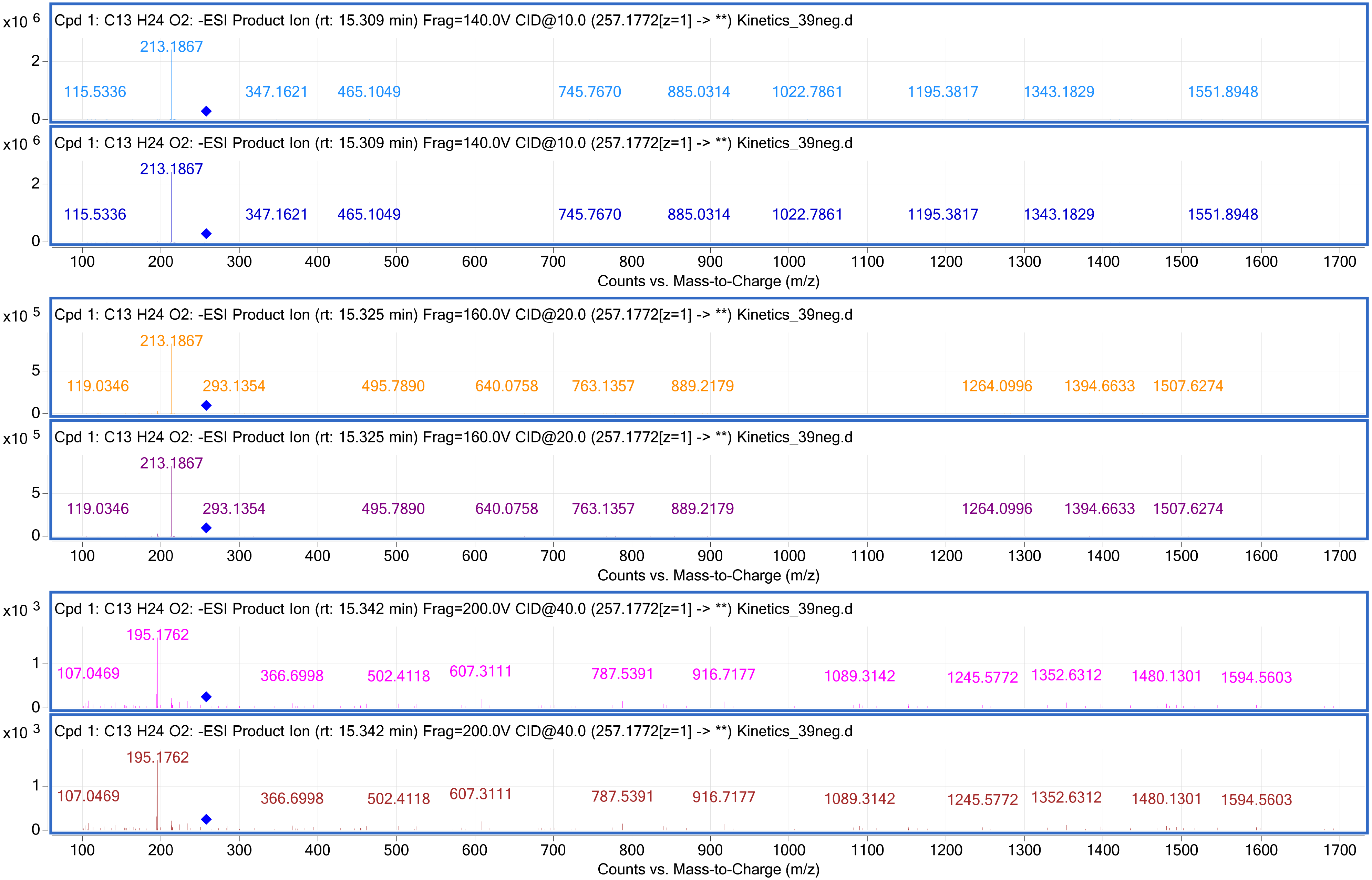

16. $C_6 H_{10} S_2$, m/z-191.0199, Di-2-propenyl disulfide

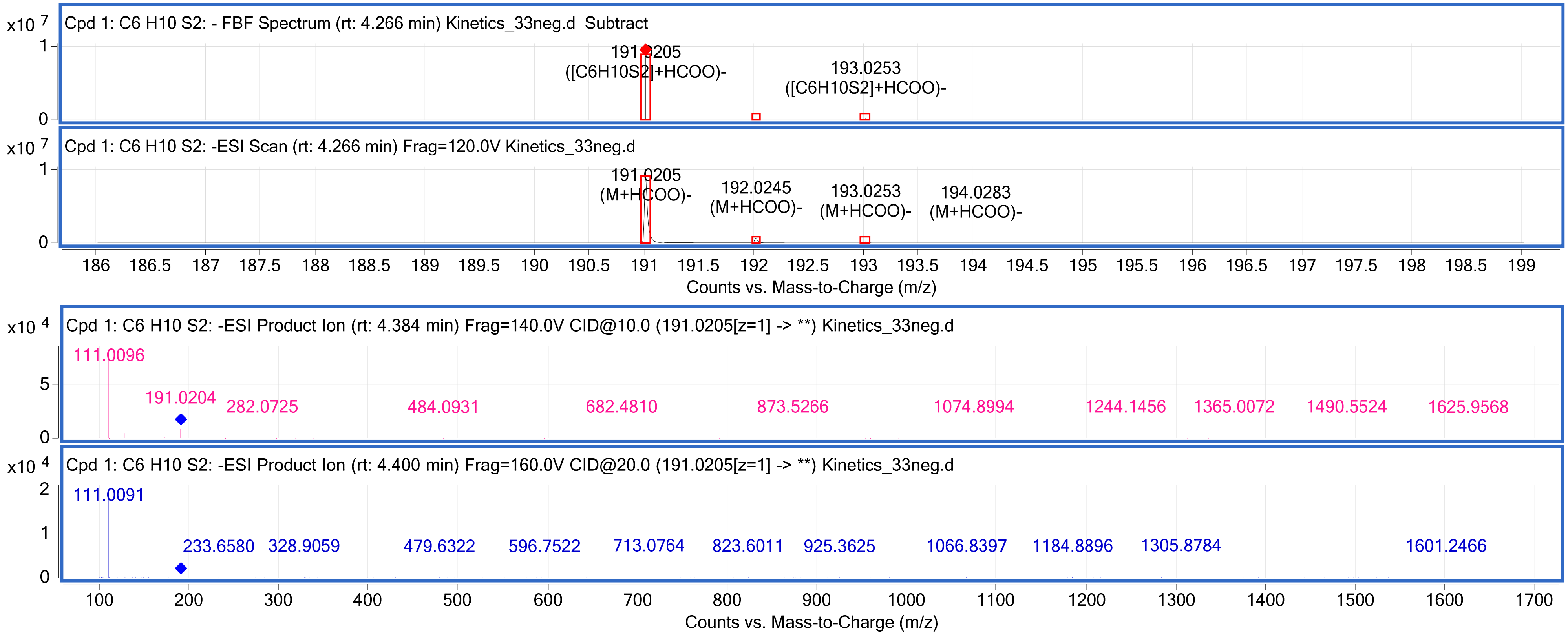

x10 7
Cpd 1: C6 H10 S2: - FBF Spectrum (rt: 4.266 min) Kinetics_33neg.d Subtract
191.0205
([C6H10S2]+HCOO)-
193.0253
([C6H10S2]+HCOO)-
Cpd 1: C6 H10 S2: -ESI Scan (rt: 4.266 min) Frag=120.0V Kinetics_33neg.d
191.0205
(M+HCOO)-
192.0245
(M+HCOO)-
193.0253
(M+HCOO)-
194.0283
(M+HCOO)-
Counts vs. Mass-to-Charge (m/z)
x10 4
Cpd 1: C6 H10 S2: -ESI Product Ion (rt: 4.384 min) Frag=140.0V CID@10.0 (191.0205[z=1] -> **) Kinetics_33neg.d
111.0096
191.0204
282.0725
484.0931
682.4810
873.5266
1074.8994
1244.1456
1365.0072
1490.5524
1625.9568
Cpd 1: C6 H10 S2: -ESI Product Ion (rt: 4.400 min) Frag=160.0V CID@20.0 (191.0205[z=1] -> **) Kinetics_33neg.d
111.0091
233.6580
328.9059
479.6322
596.7522
713.0764
823.6011
925.3625
1066.8397
1184.8896
1305.8784
1601.2466
Counts vs. Mass-to-Charge (m/z)

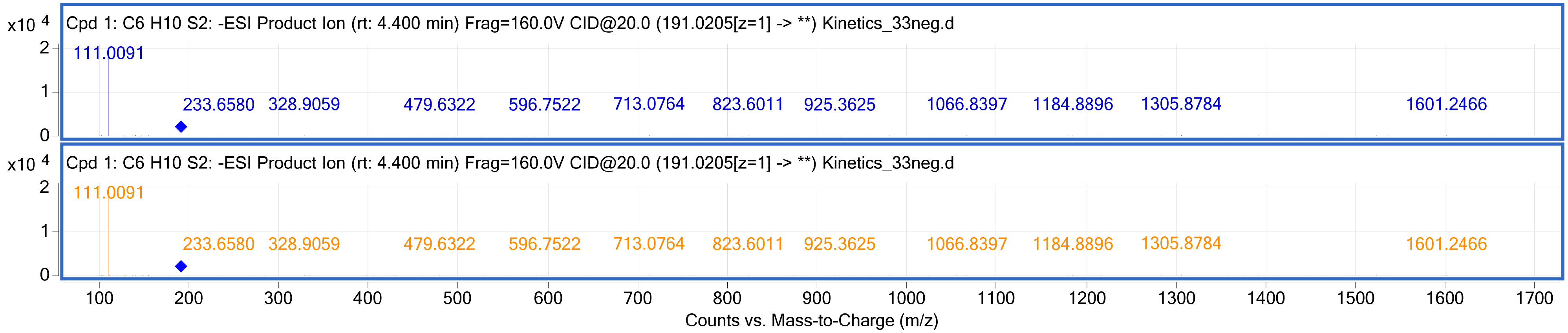

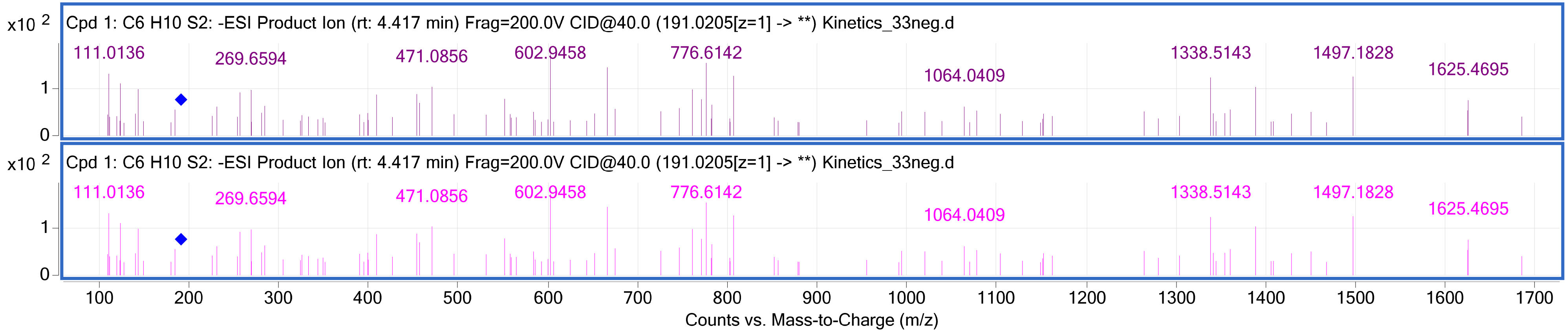

17. $C_8H_{14}O_2S_2$, m/z-205.0365, DL-alpha-Lipoic acid

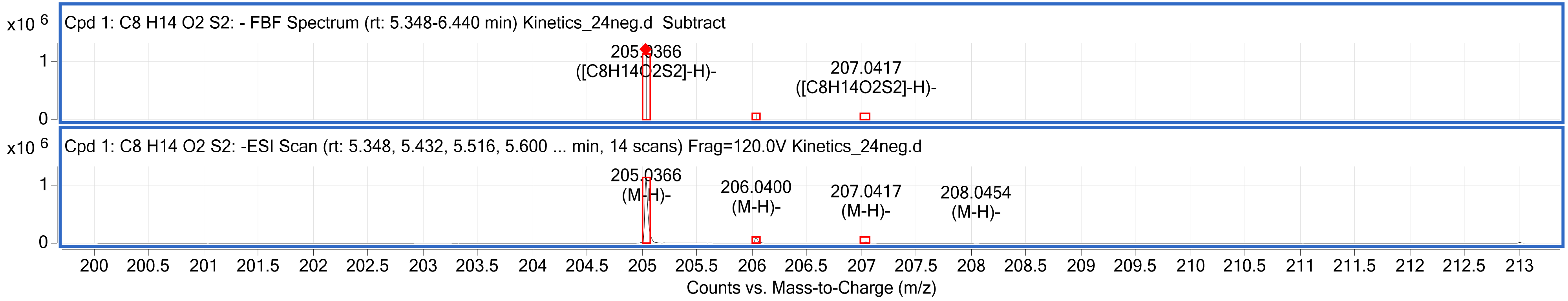

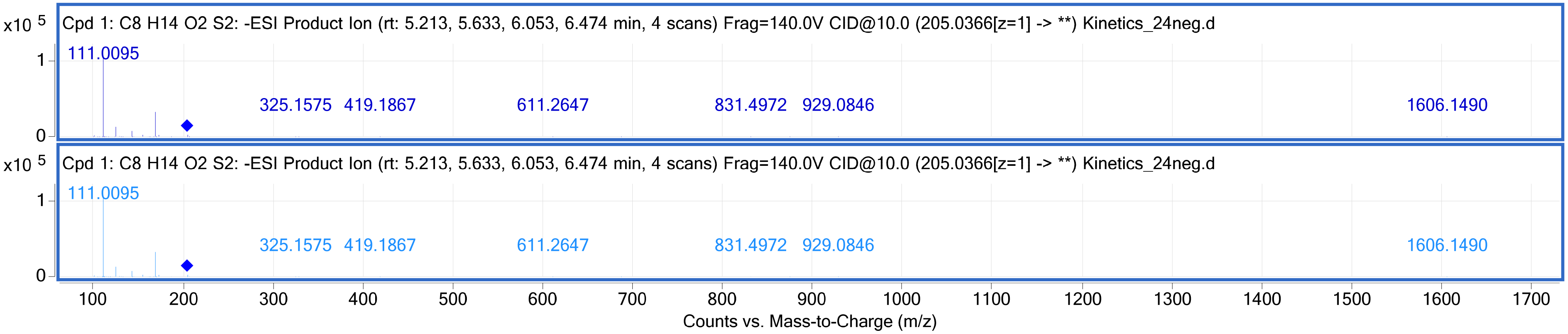

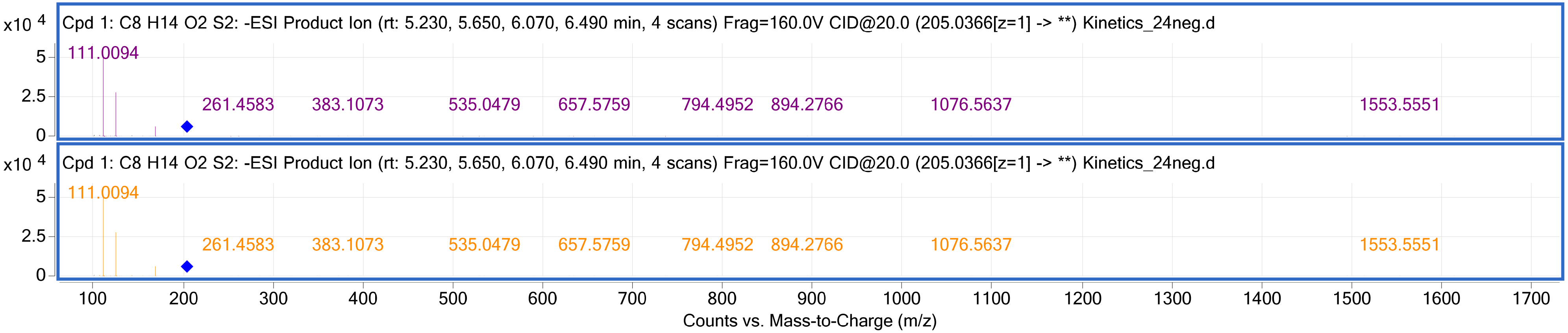

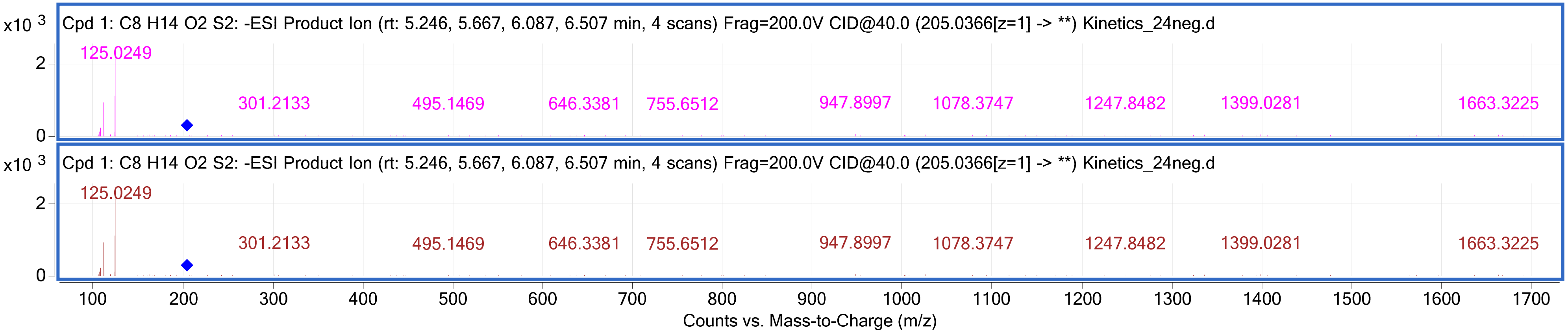

18. $C_{17}H_{26}O_4$, m/z-293.1771, Embelin

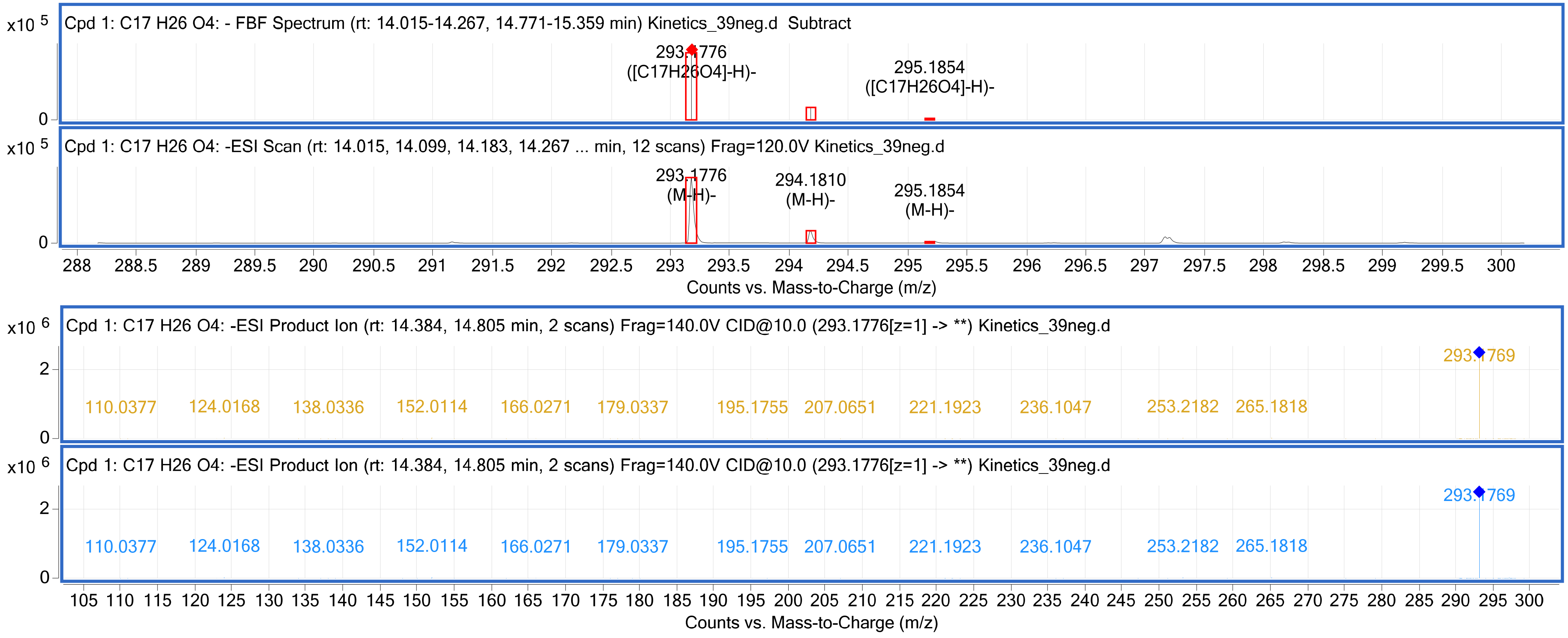
x10 5
Cpd 1: C17 H26 O4: - FBF Spectrum (rt: 14.015-14.267, 14.771-15.359 min) Kinetics_39neg.d Subtract
293.1776
([C17H26O4]-H)-
295.1854
([C17H26O4]-H)-
0
x10 5
Cpd 1: C17 H26 O4: -ESI Scan (rt: 14.015, 14.099, 14.183, 14.267 ... min, 12 scans) Frag=120.0V Kinetics_39neg.d
293.1776
(M-H)-
294.1810
(M-H)-
295.1854
(M-H)-
0
288 288.5 289 289.5 290 290.5 291 291.5 292 292.5 293 293.5 294 294.5 295 295.5 296 296.5 297 297.5 298 298.5 299 299.5 300
Counts vs. Mass-to-Charge (m/z)
x10 6
Cpd 1: C17 H26 O4: -ESI Product Ion (rt: 14.384, 14.805 min, 2 scans) Frag=140.0V CID@10.0 (293.1776[z=1] -> **) Kinetics_39neg.d
2
0
293.1769
110.0377 124.0168 138.0336 152.0114 166.0271 179.0337 195.1755 207.0651 221.1923 236.1047 253.2182 265.1818
x10 6
Cpd 1: C17 H26 O4: -ESI Product Ion (rt: 14.384, 14.805 min, 2 scans) Frag=140.0V CID@10.0 (293.1776[z=1] -> **) Kinetics_39neg.d
2
0
293.1769
110.0377 124.0168 138.0336 152.0114 166.0271 179.0337 195.1755 207.0651 221.1923 236.1047 253.2182 265.1818
105 110 115 120 125 130 135 140 145 150 155 160 165 170 175 180 185 190 195 200 205 210 215 220 225 230 235 240 245 250 255 260 265 270 275 280 285 290 295 300
Counts vs. Mass-to-Charge (m/z)

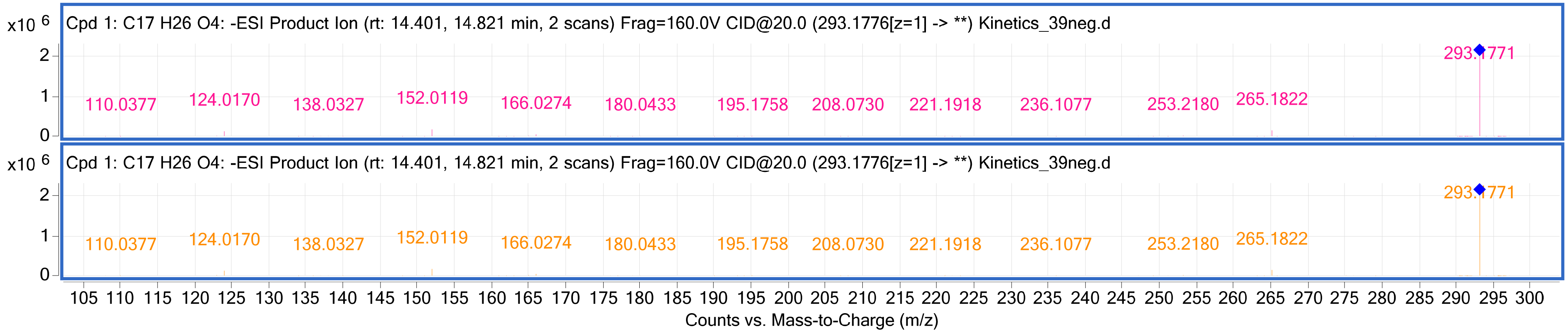

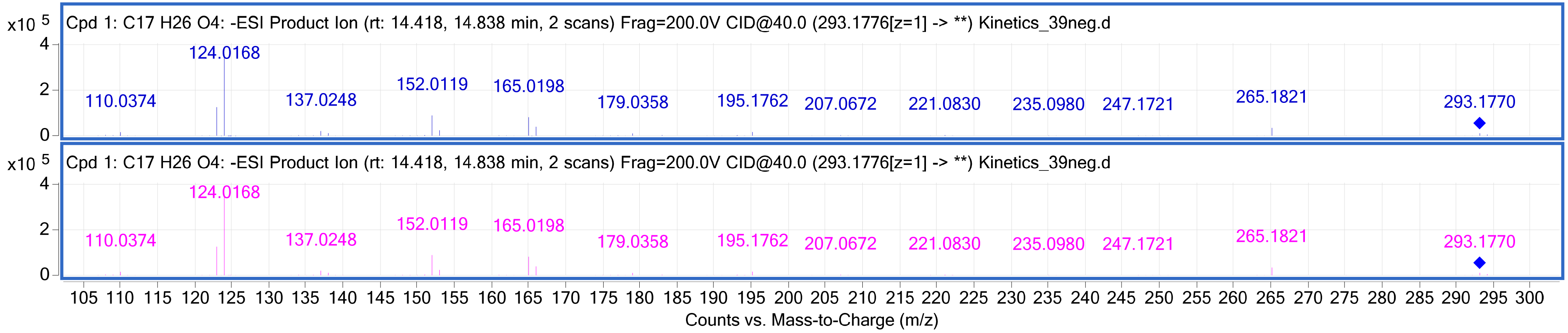

19. $C_6H_{14}S_2$, m/z-195.0514, Ethyl-1-methylpropyl disulphide

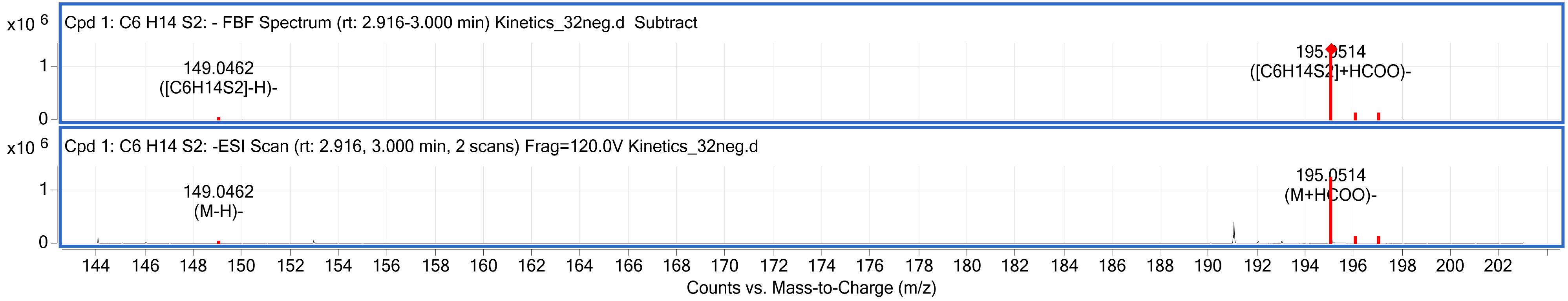

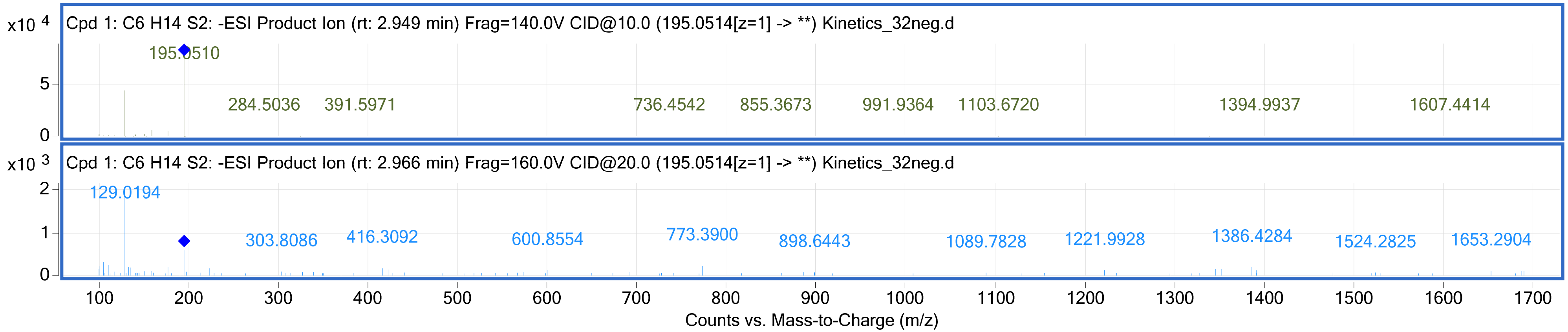

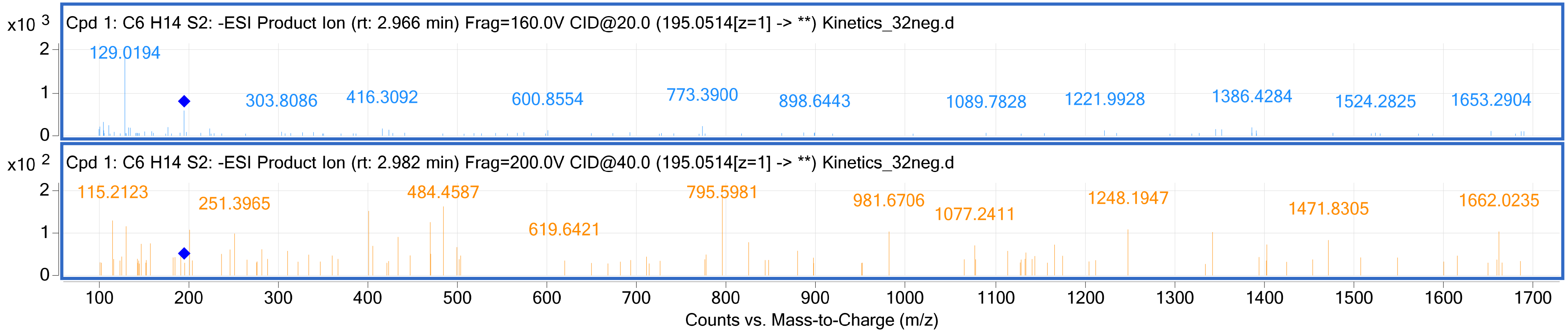

20. $C_6H_{12}O_7$, m/z-195.0514, Galactonic acid

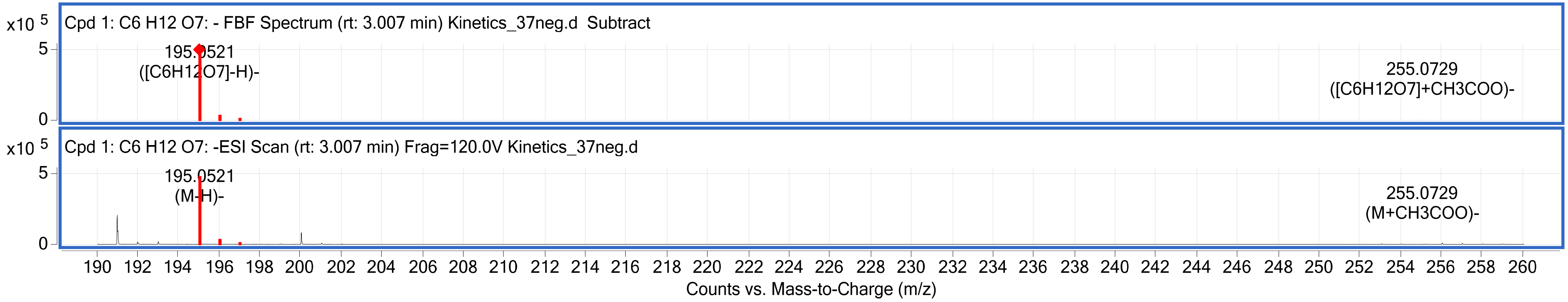

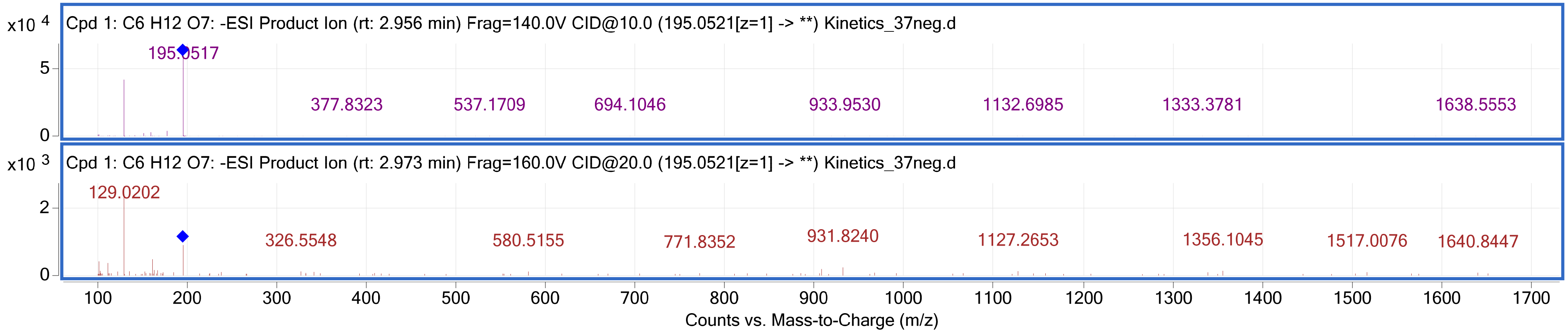

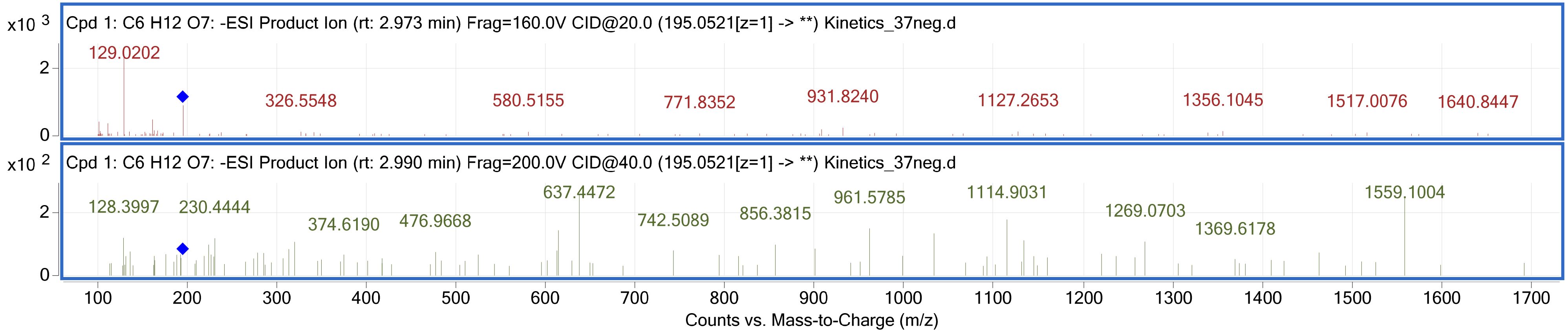

21. $C_{16}H_{30}O_4$, m/z-285.2083, Hexadecanedioic acid

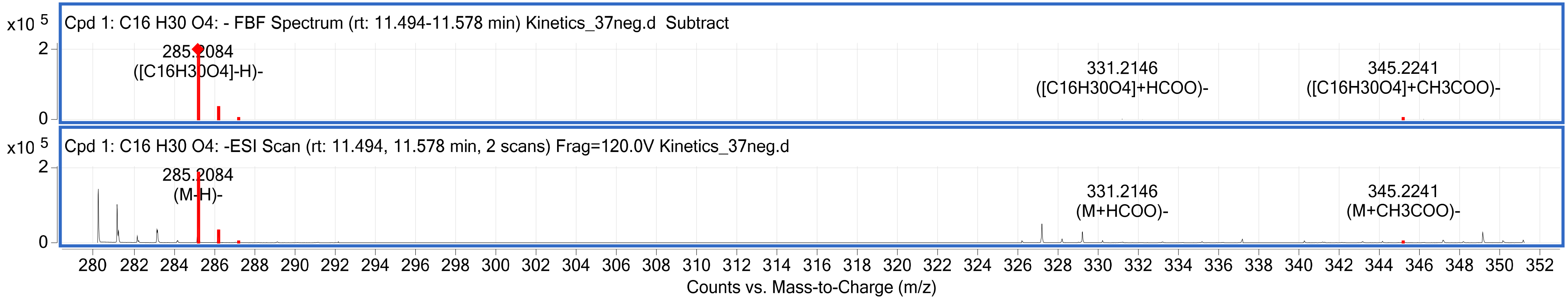

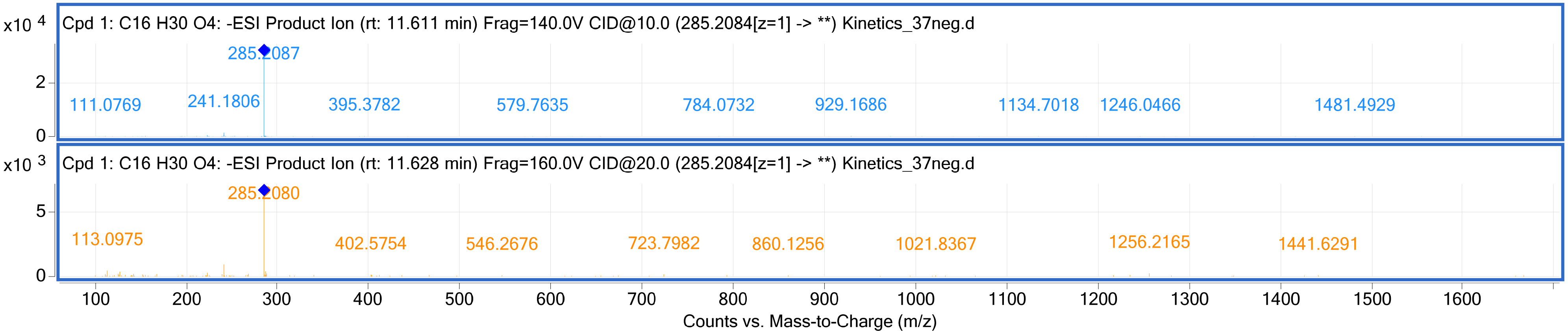

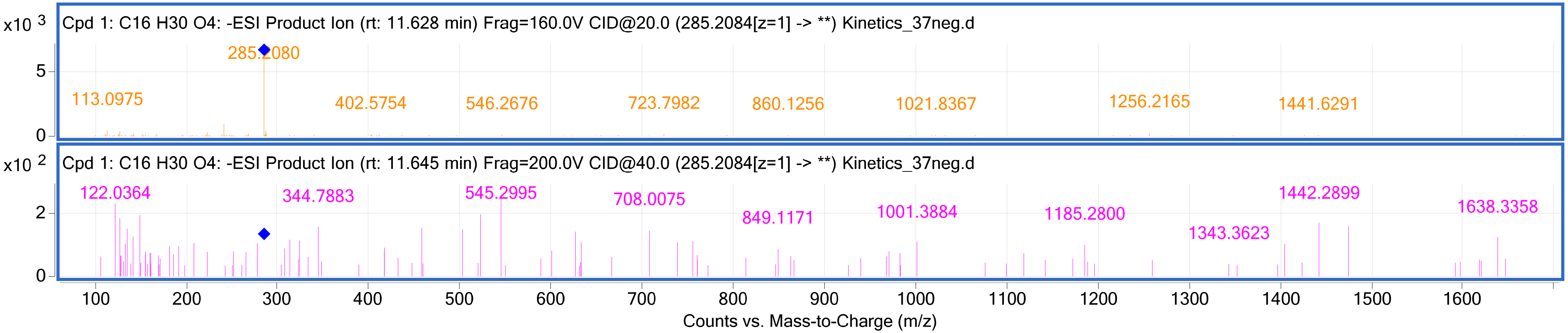

22. **$C_{21}H_{20}O_{12}$, m/z-219.135, Isoquercitrin**

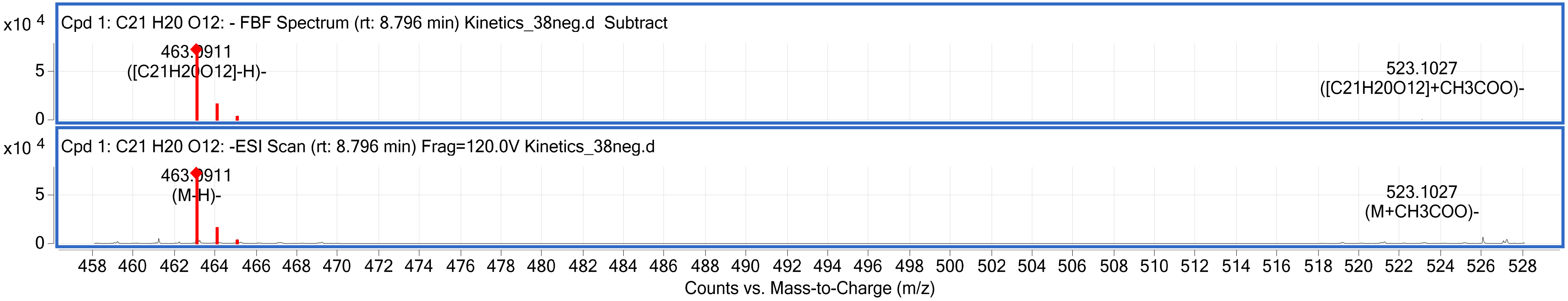

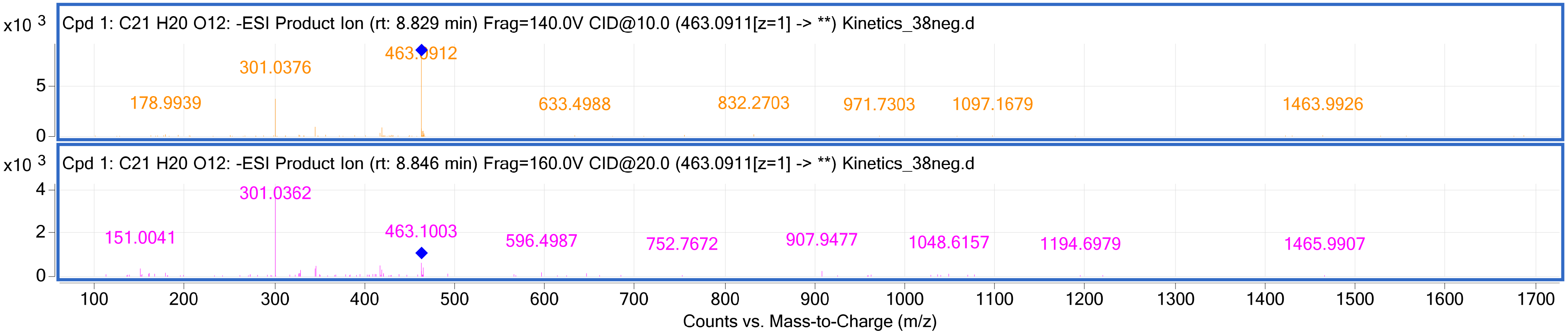

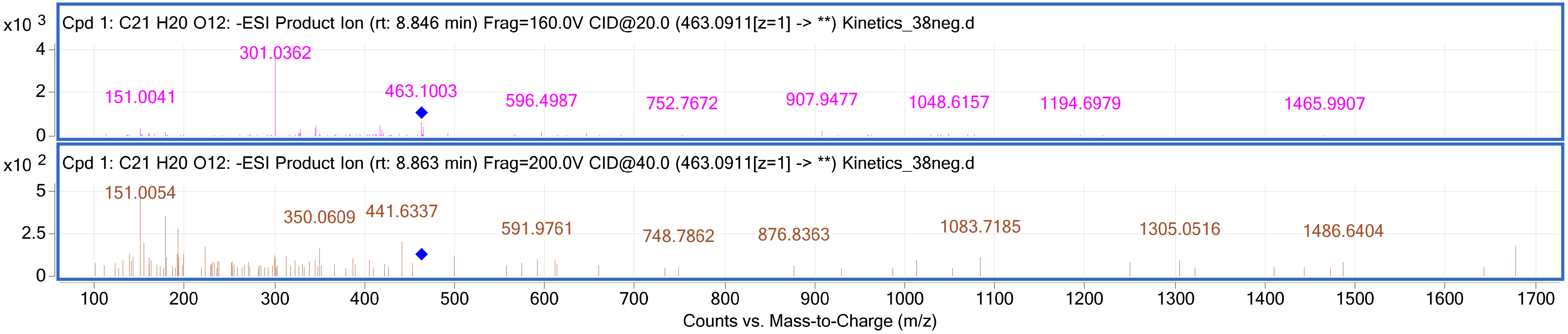

23. **$C_4H_6O_5$, m/z-133.0148, Malic acid**

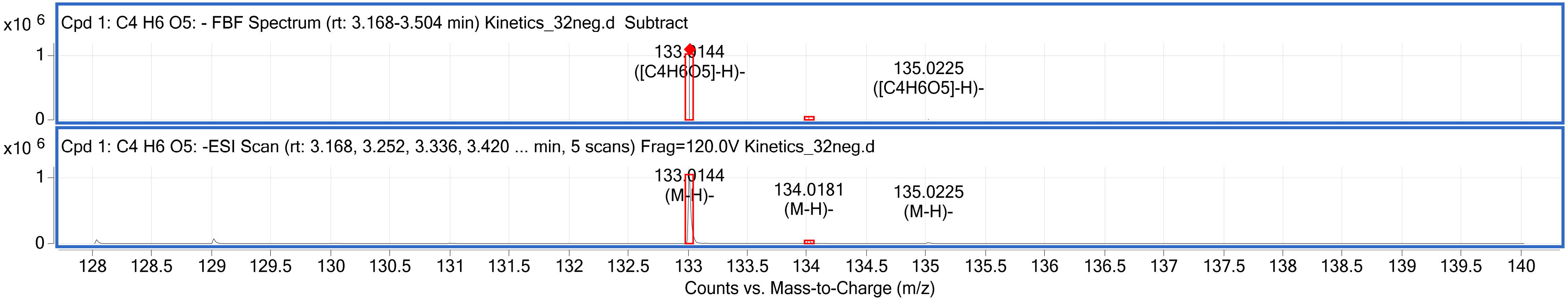

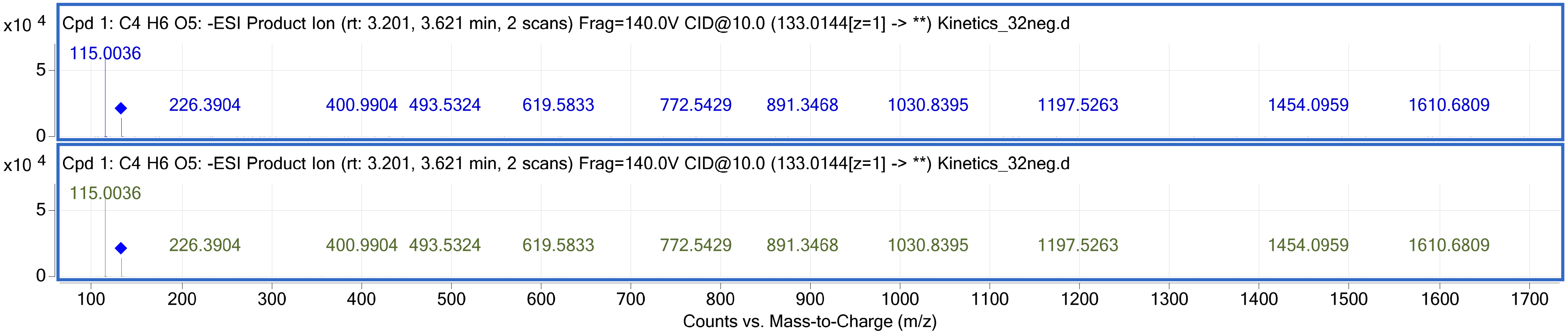

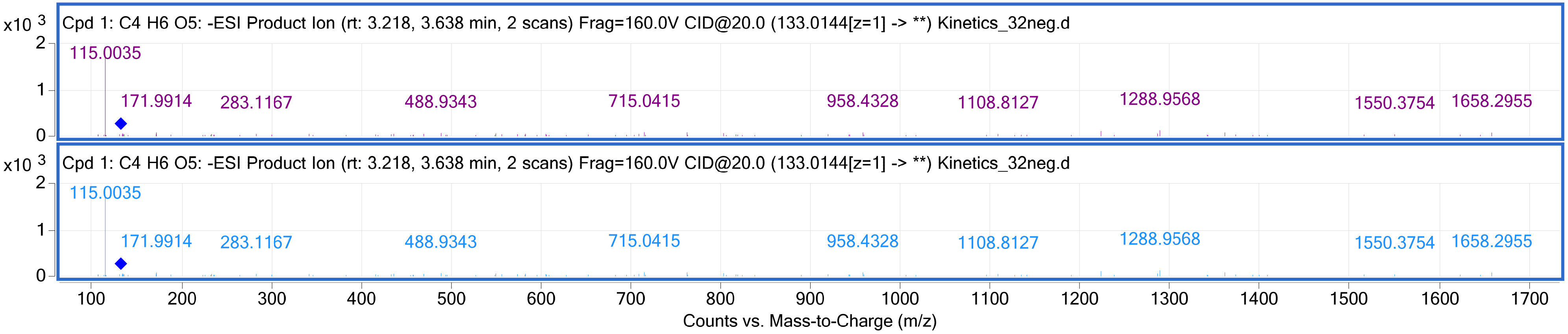

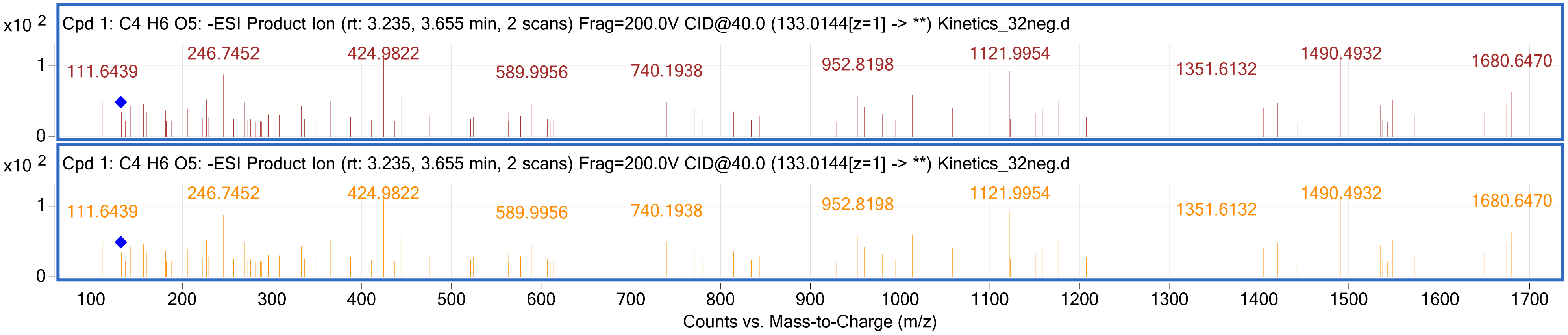

24. $C_{15}H_{12}O_5$, m/z-271.0623, Naringenin

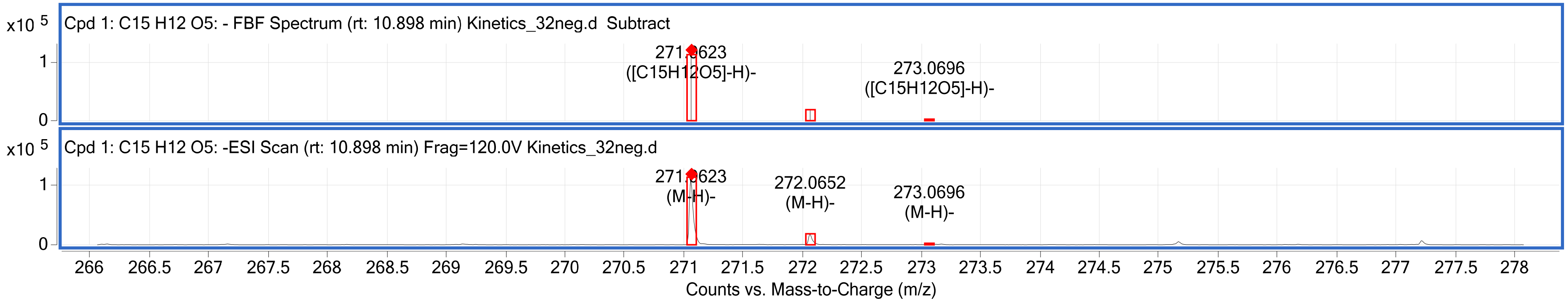

x10 5
Cpd 1: C15 H12 O5: - FBF Spectrum (rt: 10.898 min) Kinetics_32neg.d Subtract
271.0623
([C15H12O5]-H)-
273.0696
([C15H12O5]-H)-
x10 5
Cpd 1: C15 H12 O5: -ESI Scan (rt: 10.898 min) Frag=120.0V Kinetics_32neg.d
271.0623
(M-H)-
272.0652
(M-H)-
273.0696
(M-H)-
Counts vs. Mass-to-Charge (m/z)

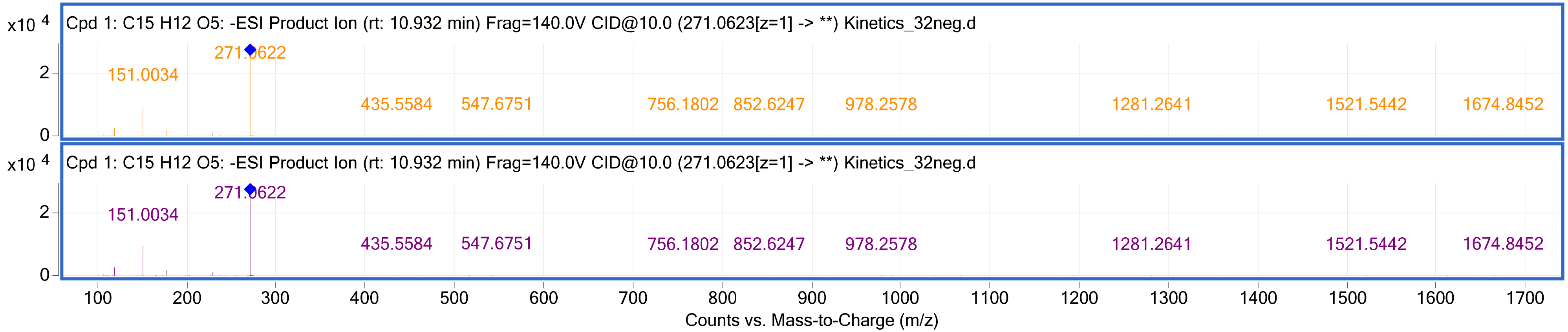

x10 4
Cpd 1: C15 H12 O5: -ESI Product Ion (rt: 10.932 min) Frag=140.0V CID@10.0 (271.0623[z=1] -> **) Kinetics_32neg.d
271.0622
151.0034
435.5584
547.6751
756.1802
852.6247
978.2578
1281.2641
1521.5442
1674.8452
x10 4
Cpd 1: C15 H12 O5: -ESI Product Ion (rt: 10.932 min) Frag=140.0V CID@10.0 (271.0623[z=1] -> **) Kinetics_32neg.d
271.0622
151.0034
435.5584
547.6751
756.1802
852.6247
978.2578
1281.2641
1521.5442
1674.8452
Counts vs. Mass-to-Charge (m/z)

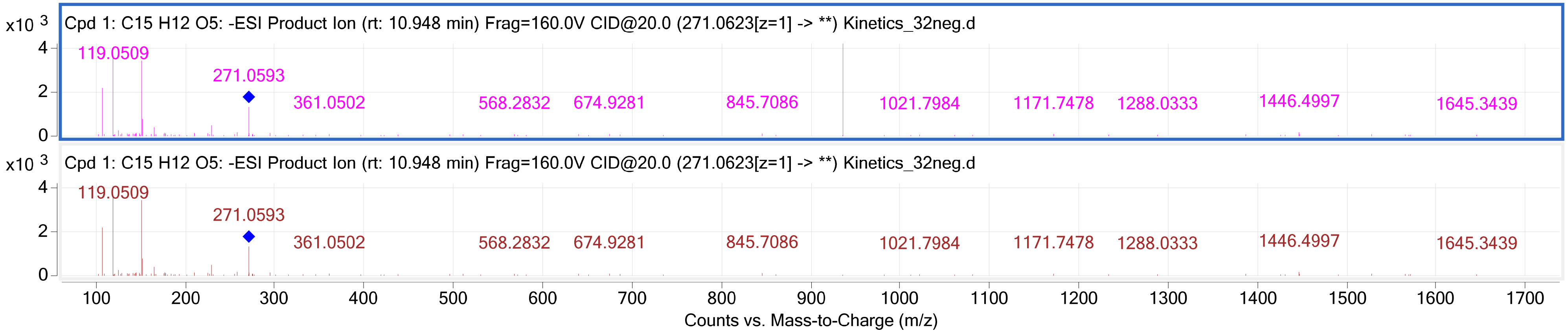

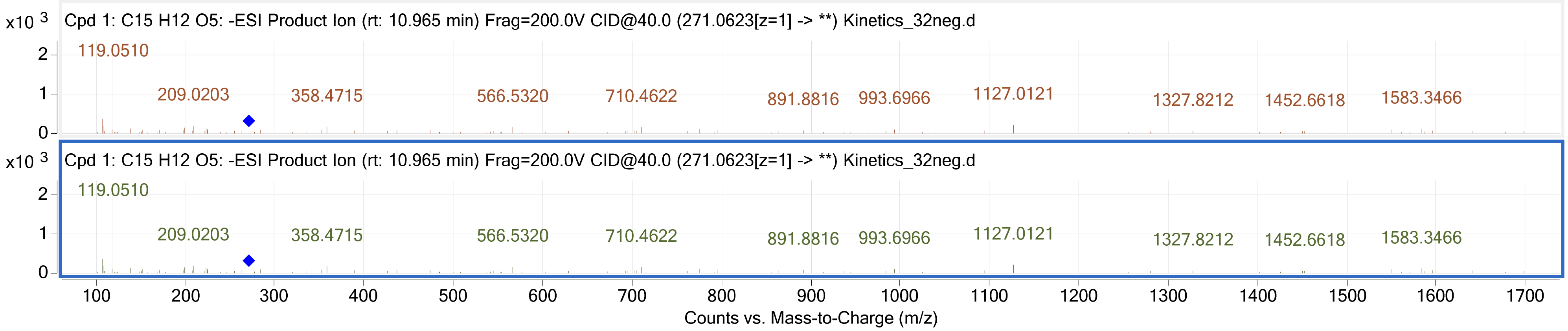

25. **$C_7H_{14}S_2$, m/z- 221.0675, N-propyl sec-butyl disulphide**

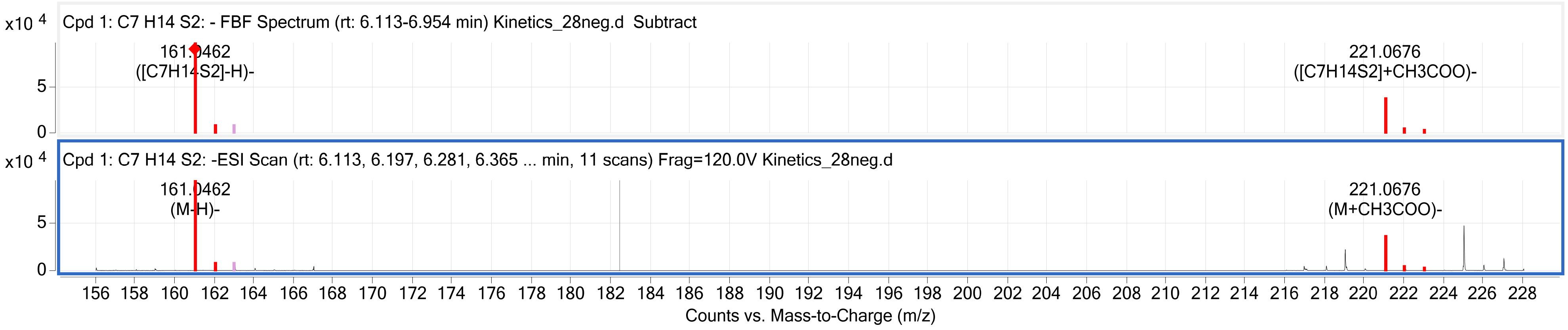

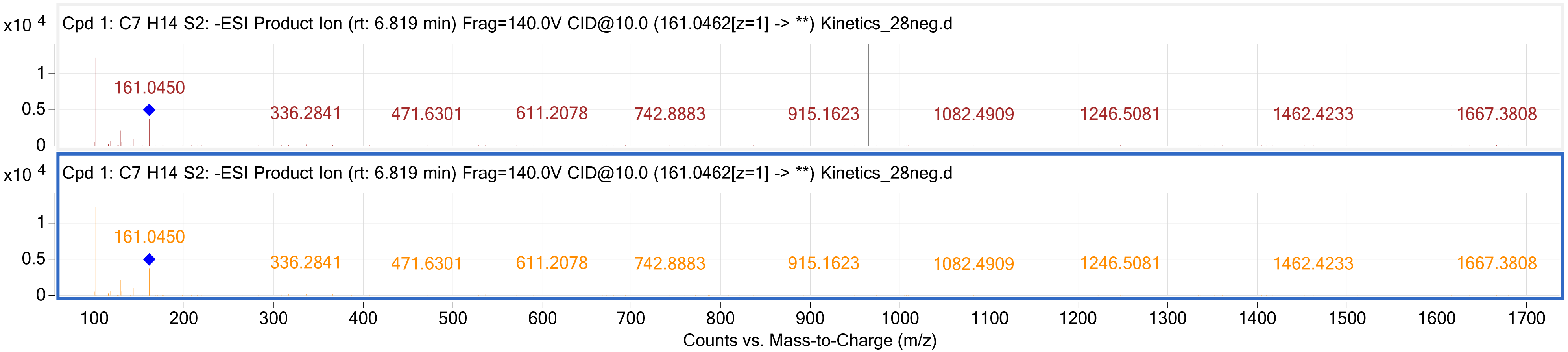
x10 4
Cpd 1: C7 H14 S2: -ESI Product Ion (rt: 6.819 min) Frag=140.0V CID@10.0 (161.0462[z=1] -> **) Kinetics_28neg.d
161.0450
336.2841
471.6301
611.2078
742.8883
915.1623
1082.4909
1246.5081
1462.4233
1667.3808
x10 4
Cpd 1: C7 H14 S2: -ESI Product Ion (rt: 6.819 min) Frag=140.0V CID@10.0 (161.0462[z=1] -> **) Kinetics_28neg.d
161.0450
336.2841
471.6301
611.2078
742.8883
915.1623
1082.4909
1246.5081
1462.4233
1667.3808
Counts vs. Mass-to-Charge (m/z)

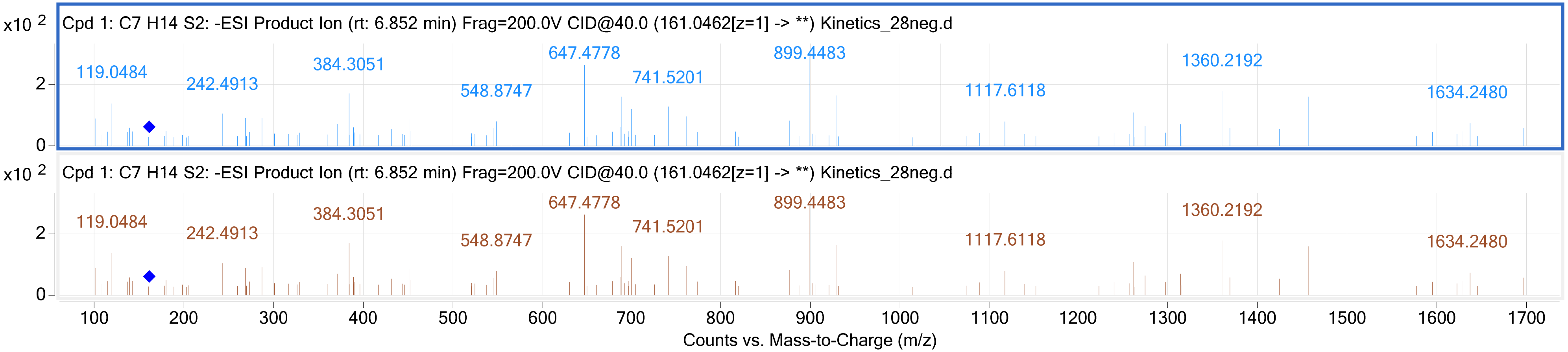
x10 2
Cpd 1: C7 H14 S2: -ESI Product Ion (rt: 6.852 min) Frag=200.0V CID@40.0 (161.0462[z=1] -> **) Kinetics_28neg.d
119.0484
242.4913
384.3051
548.8747
647.4778
741.5201
899.4483
1117.6118
1360.2192
1634.2480
x10 2
Cpd 1: C7 H14 S2: -ESI Product Ion (rt: 6.852 min) Frag=200.0V CID@40.0 (161.0462[z=1] -> **) Kinetics_28neg.d
119.0484
242.4913
384.3051
548.8747
647.4778
741.5201
899.4483
1117.6118
1360.2192
1634.2480
Counts vs. Mass-to-Charge (m/z)

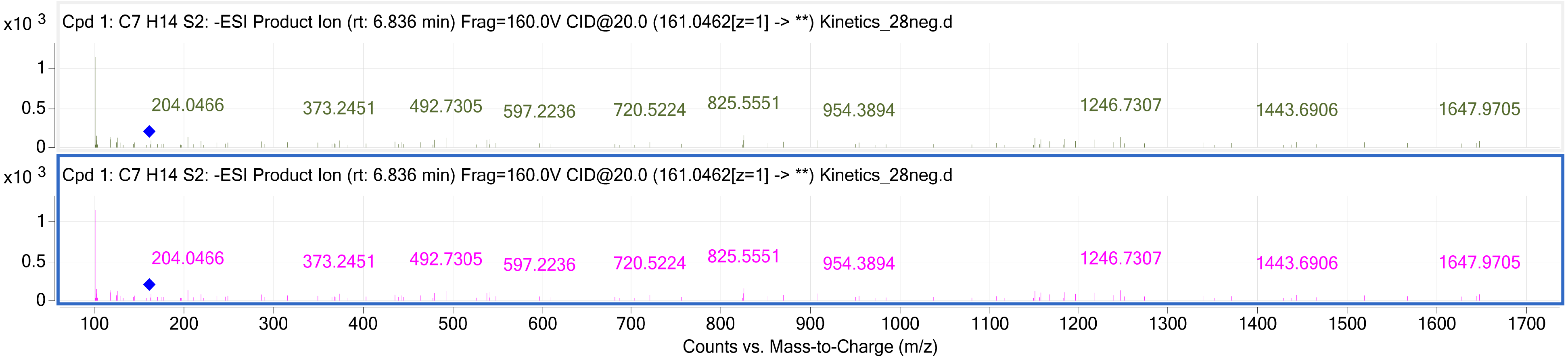
x10 3
Cpd 1: C7 H14 S2: -ESI Product Ion (rt: 6.836 min) Frag=160.0V CID@20.0 (161.0462[z=1] -> **) Kinetics_28neg.d
204.0466
373.2451
492.7305
597.2236
720.5224
825.5551
954.3894
1246.7307
1443.6906
1647.9705
x10 3
Cpd 1: C7 H14 S2: -ESI Product Ion (rt: 6.836 min) Frag=160.0V CID@20.0 (161.0462[z=1] -> **) Kinetics_28neg.d
204.0466
373.2451
492.7305
597.2236
720.5224
825.5551
954.3894
1246.7307
1443.6906
1647.9705
Counts vs. Mass-to-Charge (m/z)

26. $C_4H_4O_5$, m/z-191.0201, Oxaloacetate

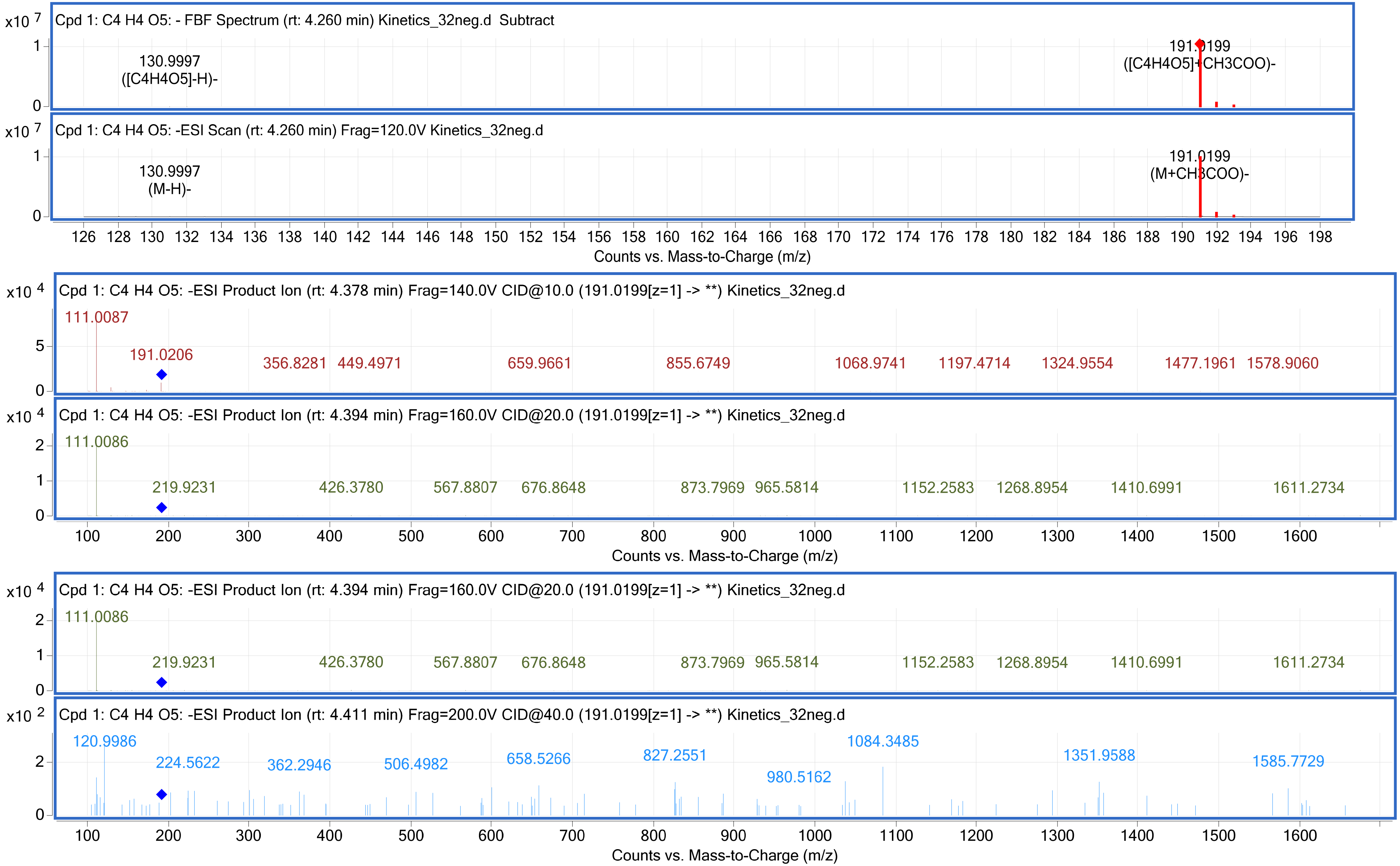

27. **$C_{14}H_{12}O_4$, m/z- 303.0885, Piceatannol**

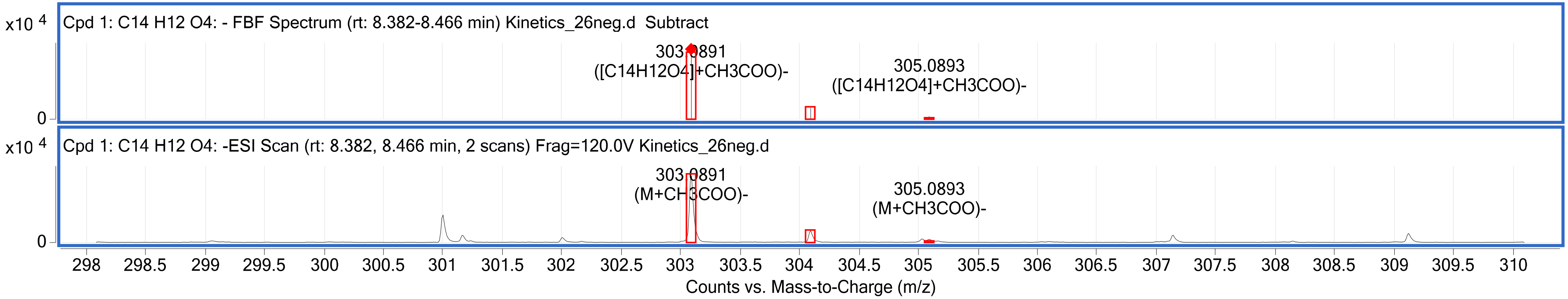

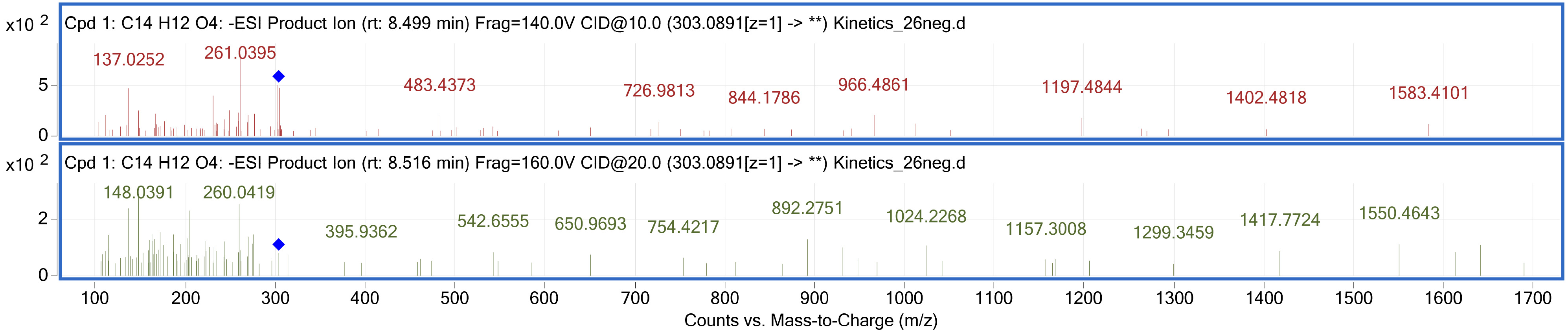

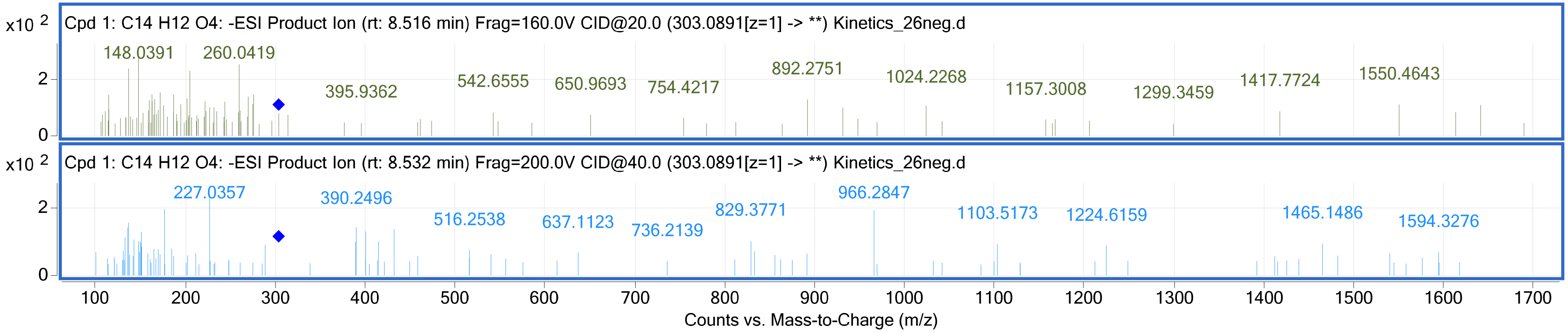

28. **$C_{15}H_{22}O_2$, m/z-293.1776, Procurcumenol**

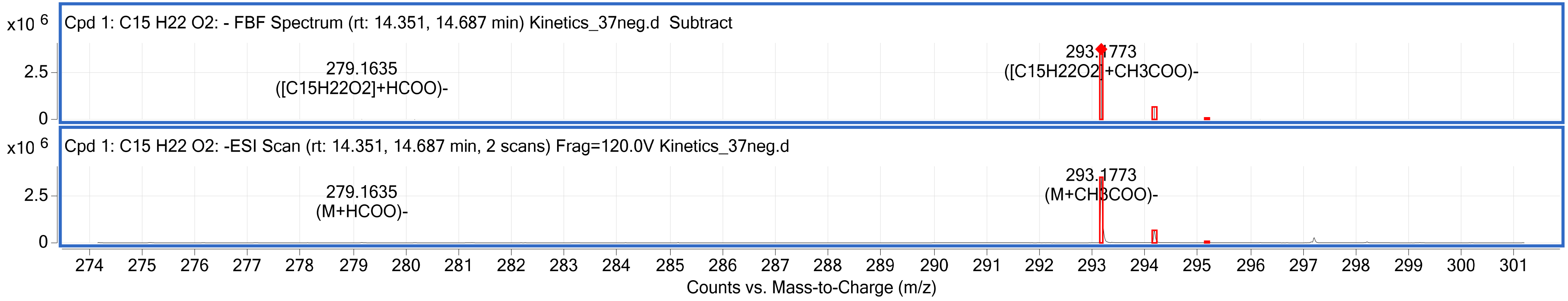

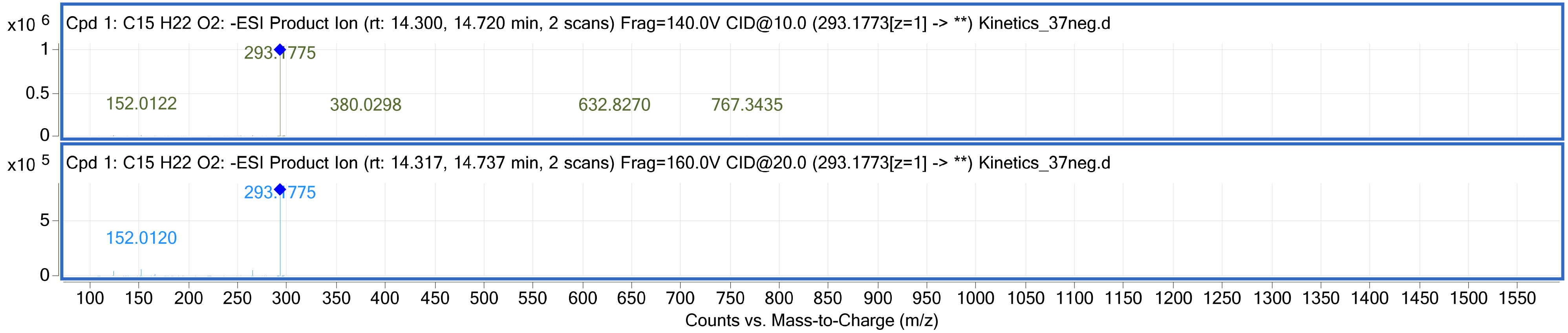

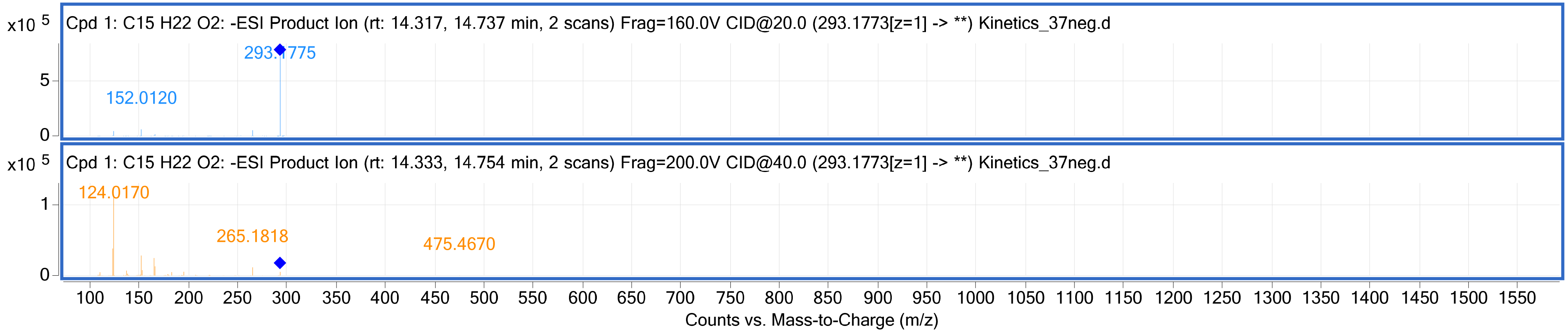

29. $C_{15}H_{10}O_7$, m/z-301.0367, Quercetin

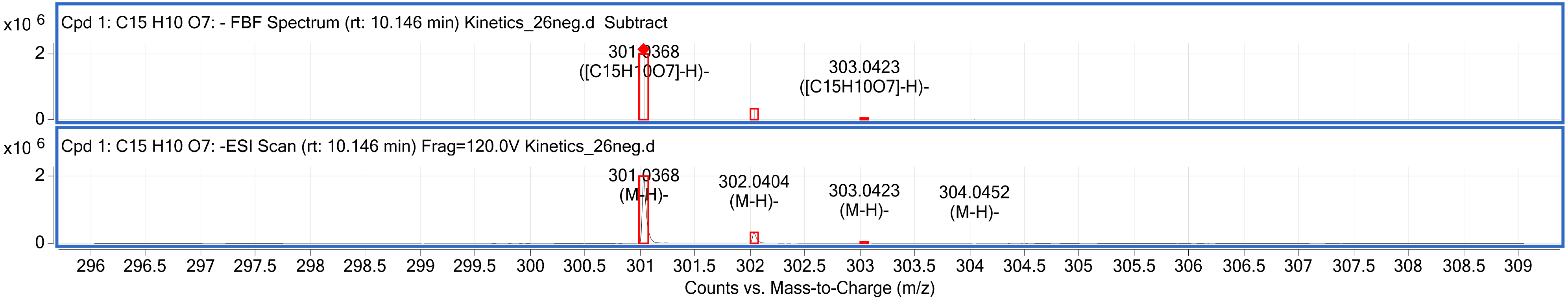

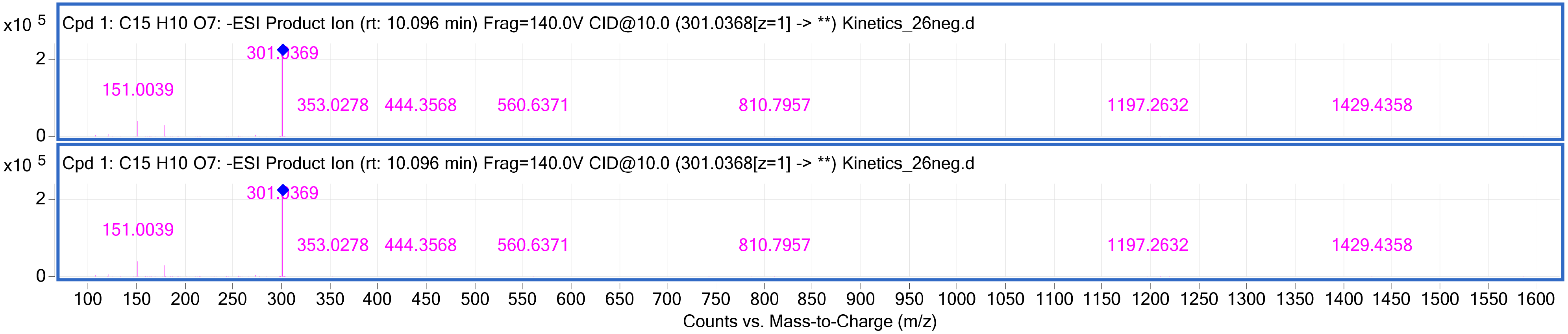

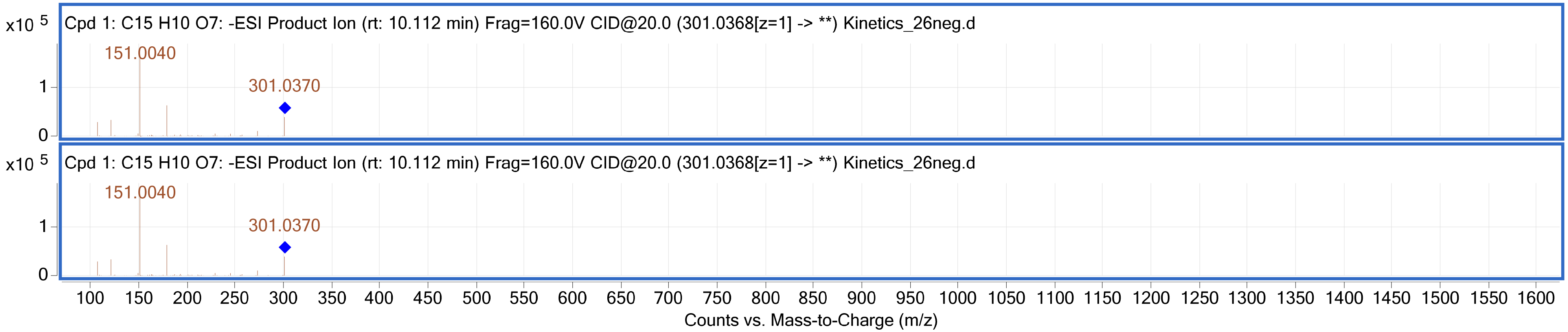
x10 5
Cpd 1: C15 H10 O7: -ESI Product Ion (rt: 10.112 min) Frag=160.0V CID@20.0 (301.0368[z=1] -> **) Kinetics_26neg.d
151.0040
301.0370
x10 5
Cpd 1: C15 H10 O7: -ESI Product Ion (rt: 10.112 min) Frag=160.0V CID@20.0 (301.0368[z=1] -> **) Kinetics_26neg.d
151.0040
301.0370
100 150 200 250 300 350 400 450 500 550 600 650 700 750 800 850 900 950 1000 1050 1100 1150 1200 1250 1300 1350 1400 1450 1500 1550 1600
Counts vs. Mass-to-Charge (m/z)

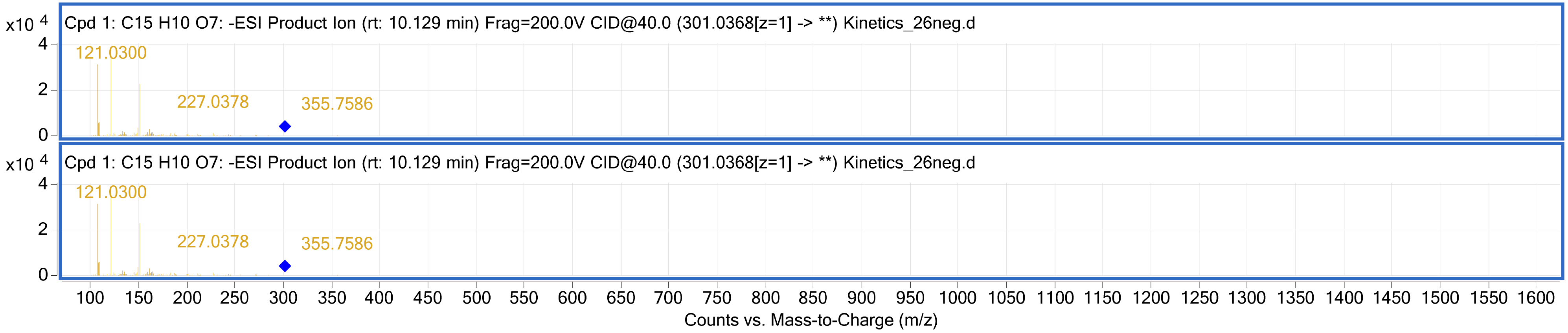
x10 4
Cpd 1: C15 H10 O7: -ESI Product Ion (rt: 10.129 min) Frag=200.0V CID@40.0 (301.0368[z=1] -> **) Kinetics_26neg.d
121.0300
227.0378
355.7586
x10 4
Cpd 1: C15 H10 O7: -ESI Product Ion (rt: 10.129 min) Frag=200.0V CID@40.0 (301.0368[z=1] -> **) Kinetics_26neg.d
121.0300
227.0378
355.7586
100 150 200 250 300 350 400 450 500 550 600 650 700 750 800 850 900 950 1000 1050 1100 1150 1200 1250 1300 1350 1400 1450 1500 1550 1600
Counts vs. Mass-to-Charge (m/z)

30. $C_{19}H_{30}O_4$, m/z-321.2093, Rapanone

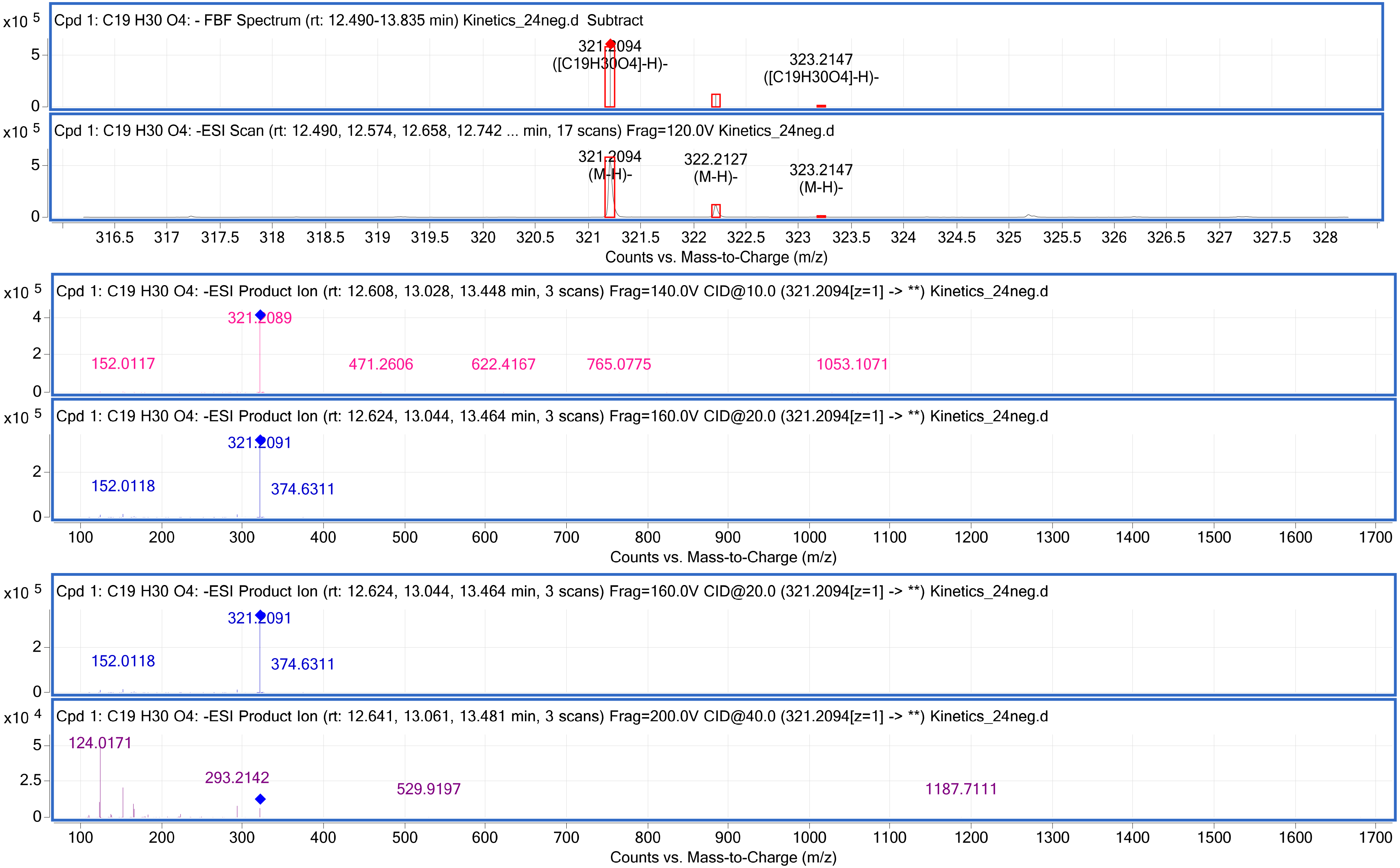

31. $C_{14}H_{28}$, m/z-255.2338, Tetradecene

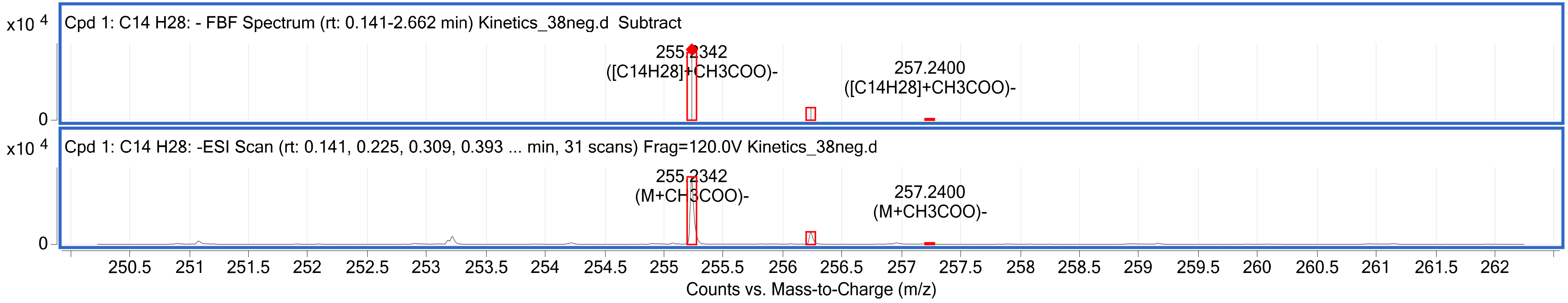

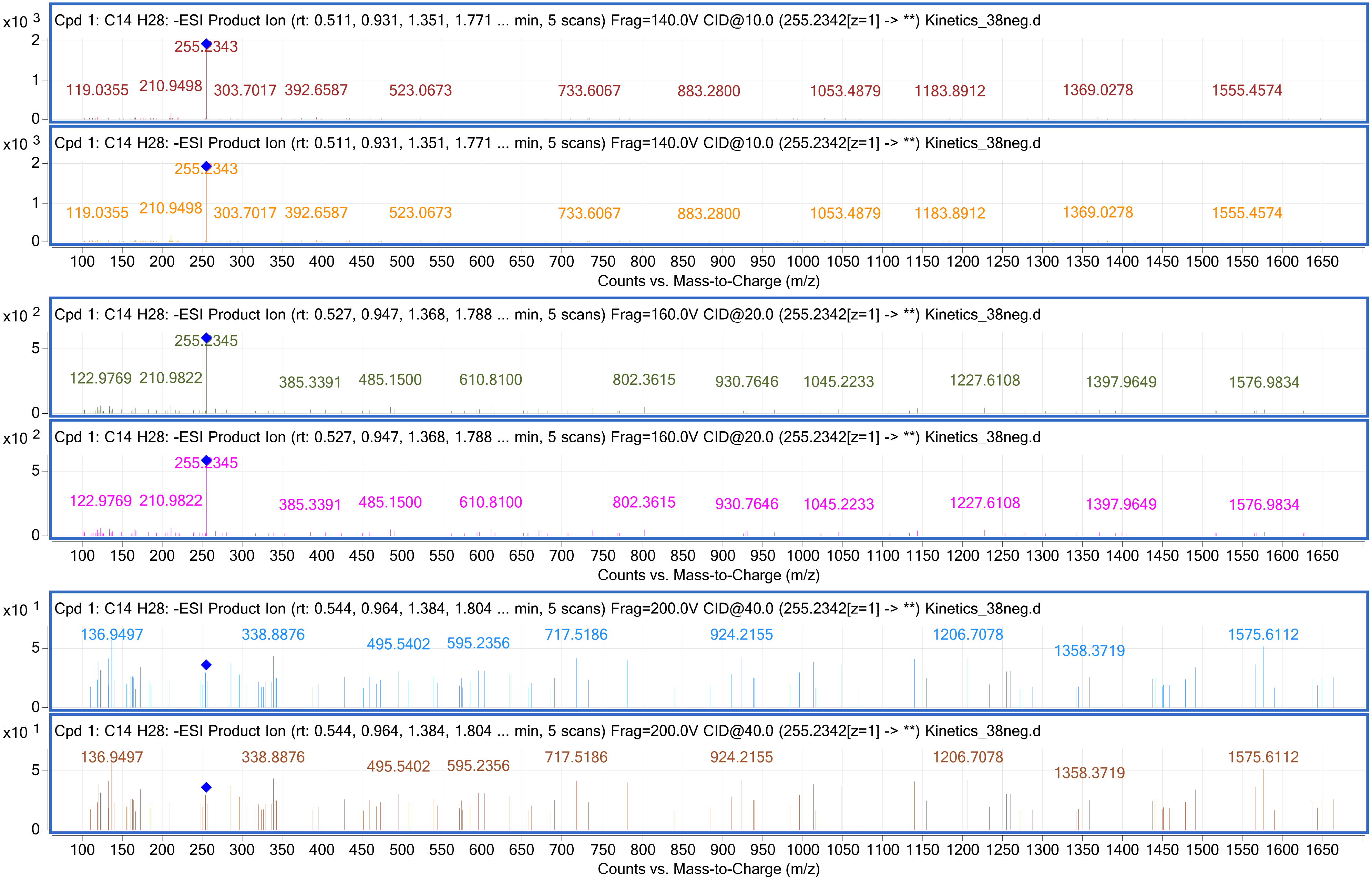
Cpd 1: C14 H28: -ESI Product Ion (rt: 0.511, 0.931, 1.351, 1.771 ... min, 5 scans) Frag=140.0V CID@10.0 (255.2342[z=1] -> **) Kinetics_38neg.d
x10 3
255.2343
119.0355 210.9498 303.7017 392.6587 523.0673 733.6067 883.2800 1053.4879 1183.8912 1369.0278 1555.4574
Cpd 1: C14 H28: -ESI Product Ion (rt: 0.511, 0.931, 1.351, 1.771 ... min, 5 scans) Frag=140.0V CID@10.0 (255.2342[z=1] -> **) Kinetics_38neg.d
x10 3
255.2343
119.0355 210.9498 303.7017 392.6587 523.0673 733.6067 883.2800 1053.4879 1183.8912 1369.0278 1555.4574
Counts vs. Mass-to-Charge (m/z)
Cpd 1: C14 H28: -ESI Product Ion (rt: 0.527, 0.947, 1.368, 1.788 ... min, 5 scans) Frag=160.0V CID@20.0 (255.2342[z=1] -> **) Kinetics_38neg.d
x10 2
255.2345
122.9769 210.9822 385.3391 485.1500 610.8100 802.3615 930.7646 1045.2233 1227.6108 1397.9649 1576.9834
Cpd 1: C14 H28: -ESI Product Ion (rt: 0.527, 0.947, 1.368, 1.788 ... min, 5 scans) Frag=160.0V CID@20.0 (255.2342[z=1] -> **) Kinetics_38neg.d
x10 2
255.2345
122.9769 210.9822 385.3391 485.1500 610.8100 802.3615 930.7646 1045.2233 1227.6108 1397.9649 1576.9834
Counts vs. Mass-to-Charge (m/z)
Cpd 1: C14 H28: -ESI Product Ion (rt: 0.544, 0.964, 1.384, 1.804 ... min, 5 scans) Frag=200.0V CID@40.0 (255.2342[z=1] -> **) Kinetics_38neg.d
x10 1
136.9497 338.8876 495.5402 595.2356 717.5186 924.2155 1206.7078 1358.3719 1575.6112
Cpd 1: C14 H28: -ESI Product Ion (rt: 0.544, 0.964, 1.384, 1.804 ... min, 5 scans) Frag=200.0V CID@40.0 (255.2342[z=1] -> **) Kinetics_38neg.d
x10 1
136.9497 338.8876 495.5402 595.2356 717.5186 924.2155 1206.7078 1358.3719 1575.6112
Counts vs. Mass-to-Charge (m/z)

32. $C_{11}H_{22}O$, m/z-229.1817, Undecan-2-one

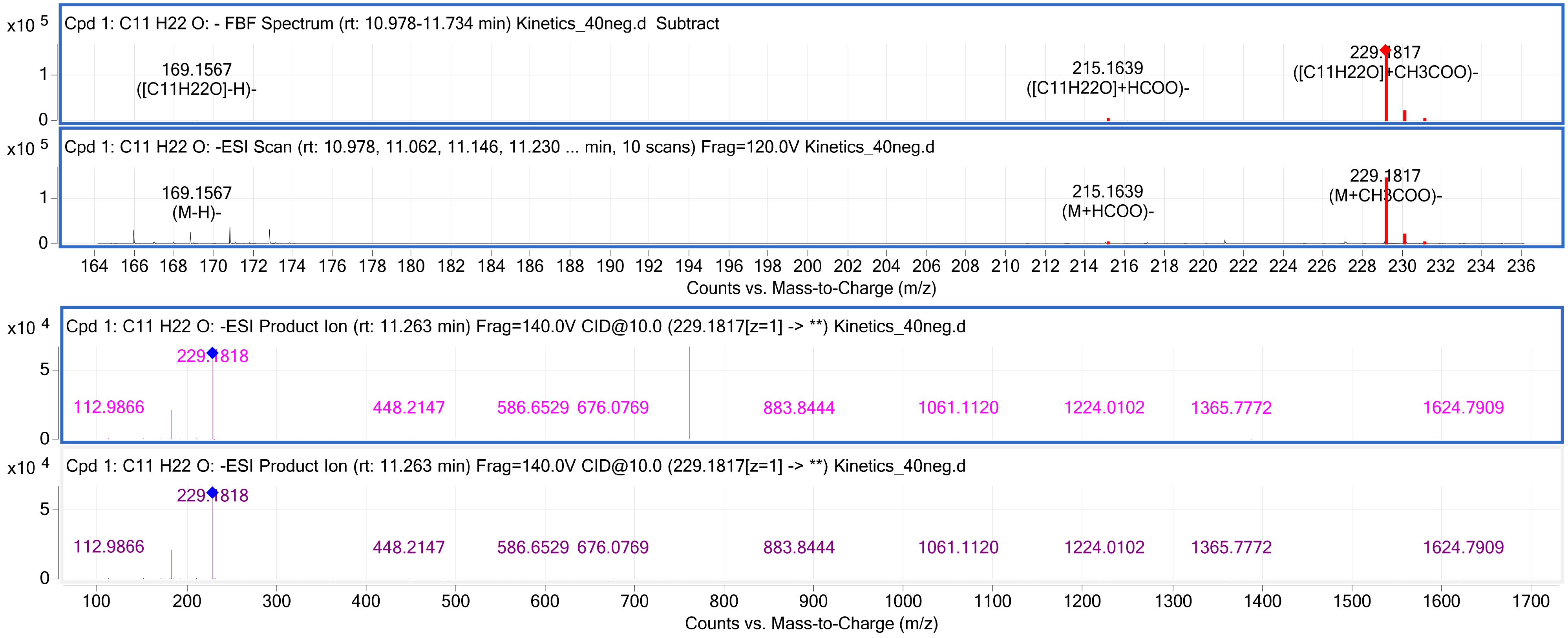

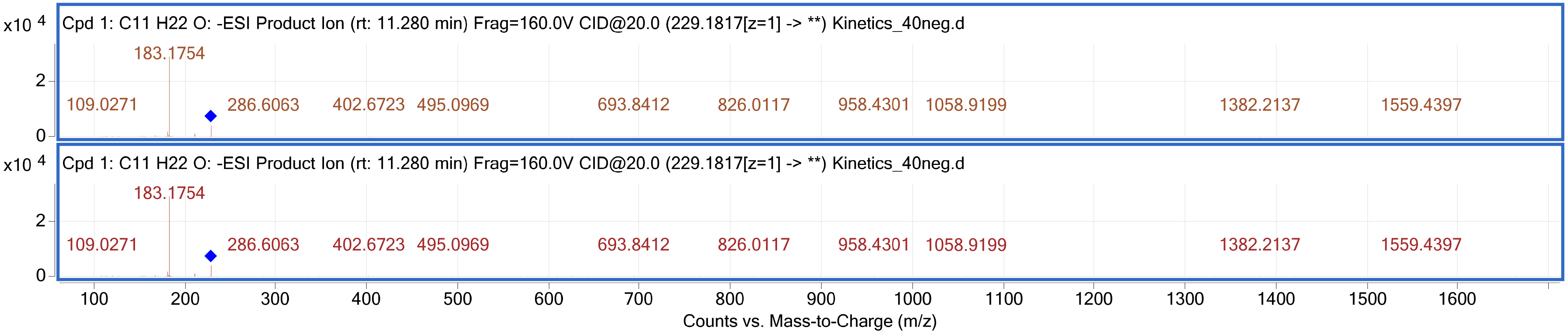

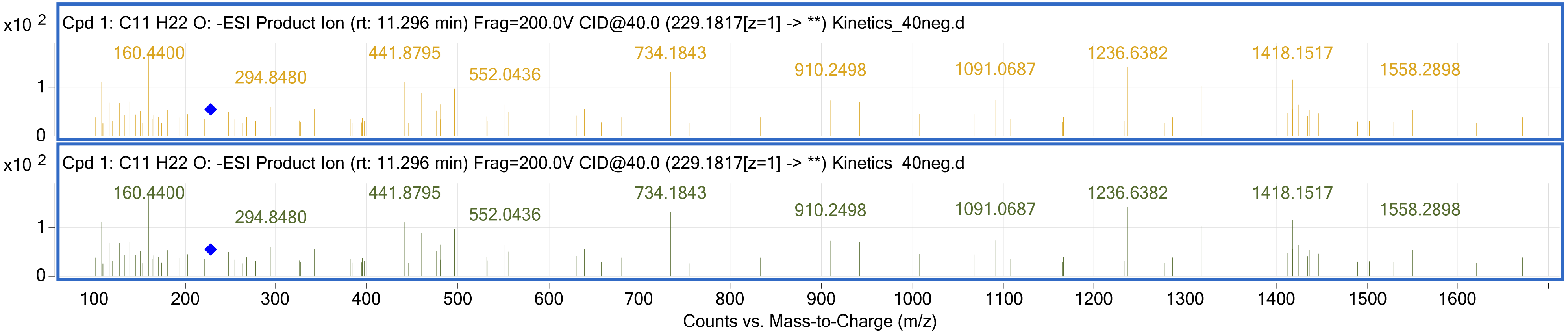

33. $C_8H_8O_4$, m/z-167.0356, Vanillic acid

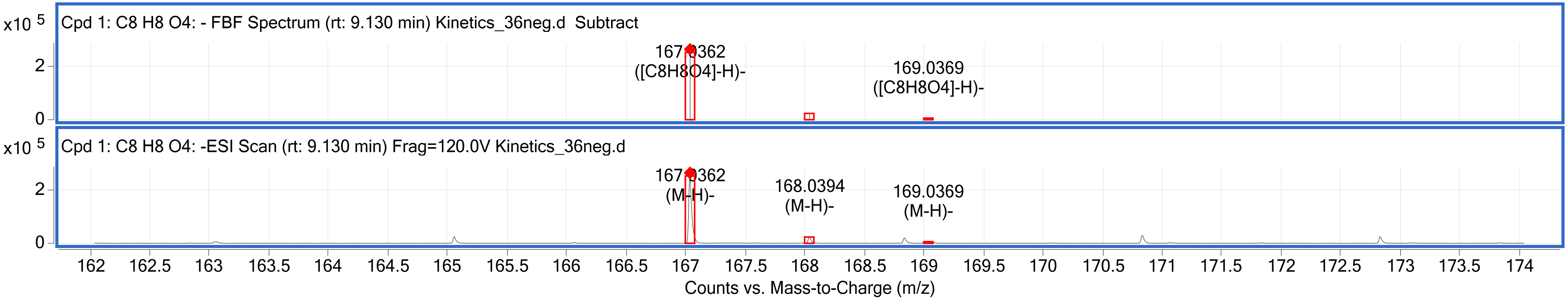

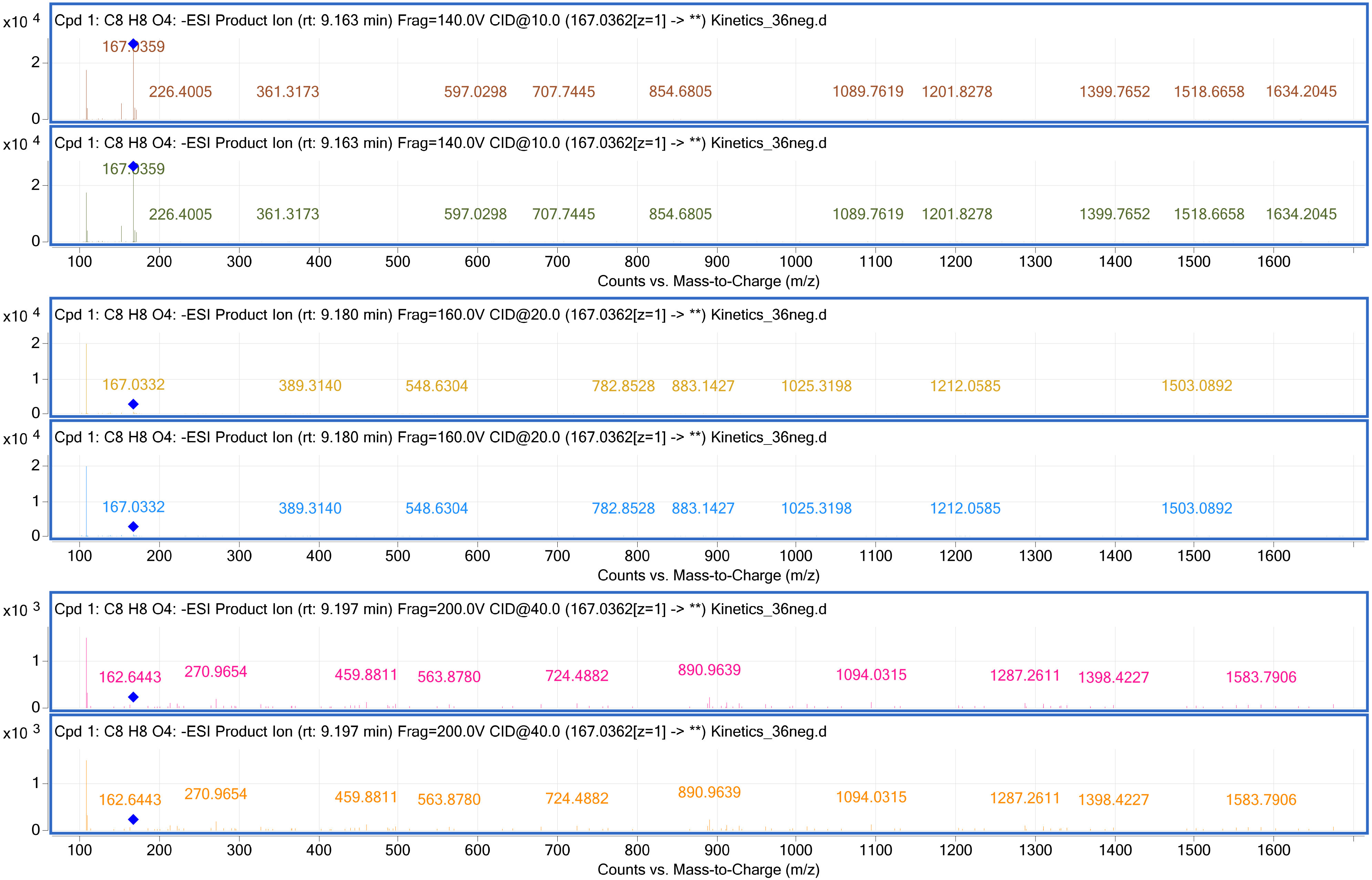

x10 4
Cpd 1: C8 H8 O4: -ESI Product Ion (rt: 9.163 min) Frag=140.0V CID@10.0 (167.0362[z=1] -> **) Kinetics_36neg.d
167.0359
226.4005
361.3173
597.0298
707.7445
854.6805
1089.7619
1201.8278
1399.7652
1518.6658
1634.2045
x10 4
Cpd 1: C8 H8 O4: -ESI Product Ion (rt: 9.163 min) Frag=140.0V CID@10.0 (167.0362[z=1] -> **) Kinetics_36neg.d
167.0359
226.4005
361.3173
597.0298
707.7445
854.6805
1089.7619
1201.8278
1399.7652
1518.6658
1634.2045
Counts vs. Mass-to-Charge (m/z)
x10 4
Cpd 1: C8 H8 O4: -ESI Product Ion (rt: 9.180 min) Frag=160.0V CID@20.0 (167.0362[z=1] -> **) Kinetics_36neg.d
167.0332
389.3140
548.6304
782.8528
883.1427
1025.3198
1212.0585
1503.0892
x10 4
Cpd 1: C8 H8 O4: -ESI Product Ion (rt: 9.180 min) Frag=160.0V CID@20.0 (167.0362[z=1] -> **) Kinetics_36neg.d
167.0332
389.3140
548.6304
782.8528
883.1427
1025.3198
1212.0585
1503.0892
Counts vs. Mass-to-Charge (m/z)
x10 3
Cpd 1: C8 H8 O4: -ESI Product Ion (rt: 9.197 min) Frag=200.0V CID@40.0 (167.0362[z=1] -> **) Kinetics_36neg.d
162.6443
270.9654
459.8811
563.8780
724.4882
890.9639
1094.0315
1287.2611
1398.4227
1583.7906
x10 3
Cpd 1: C8 H8 O4: -ESI Product Ion (rt: 9.197 min) Frag=200.0V CID@40.0 (167.0362[z=1] -> **) Kinetics_36neg.d
162.6443
270.9654
459.8811
563.8780
724.4882
890.9639
1094.0315
1287.2611
1398.4227
1583.7906
Counts vs. Mass-to-Charge (m/z)

34. $C_{18}H_{28}N_2O_4$, m/z-337.2135, Acebutolol

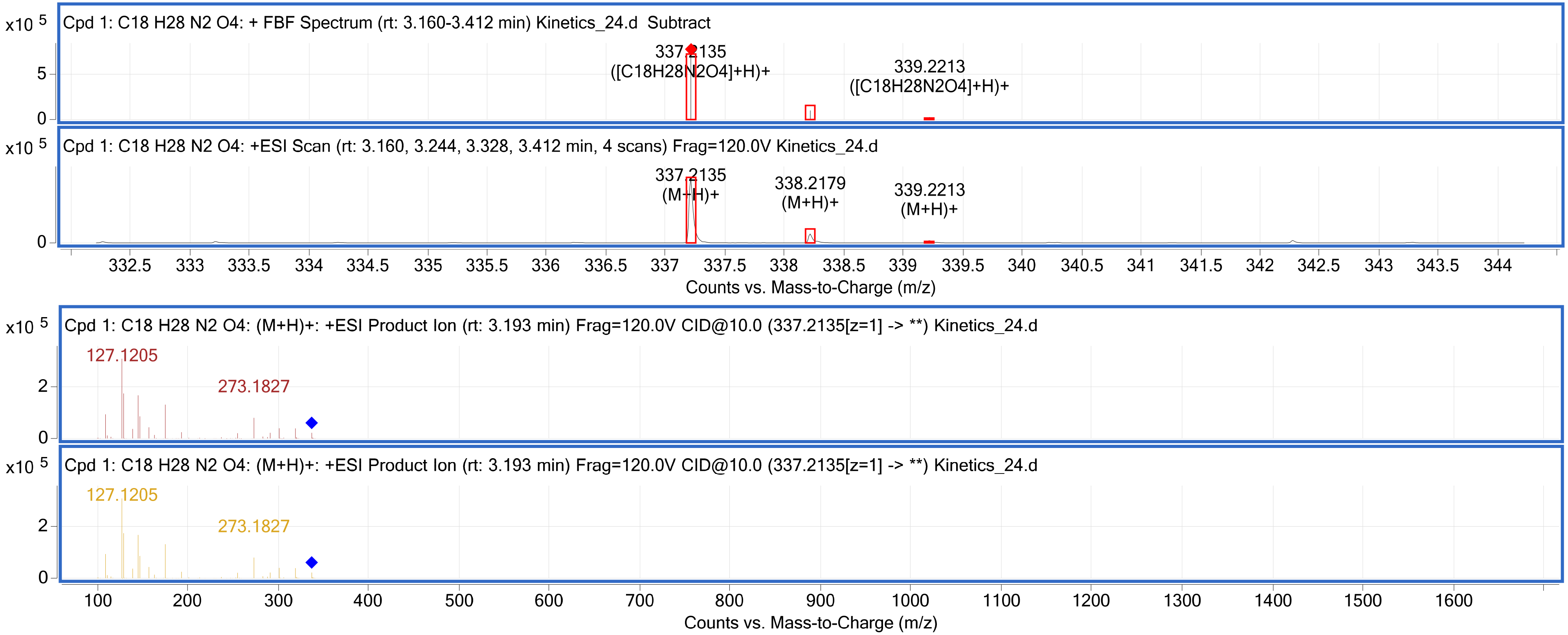

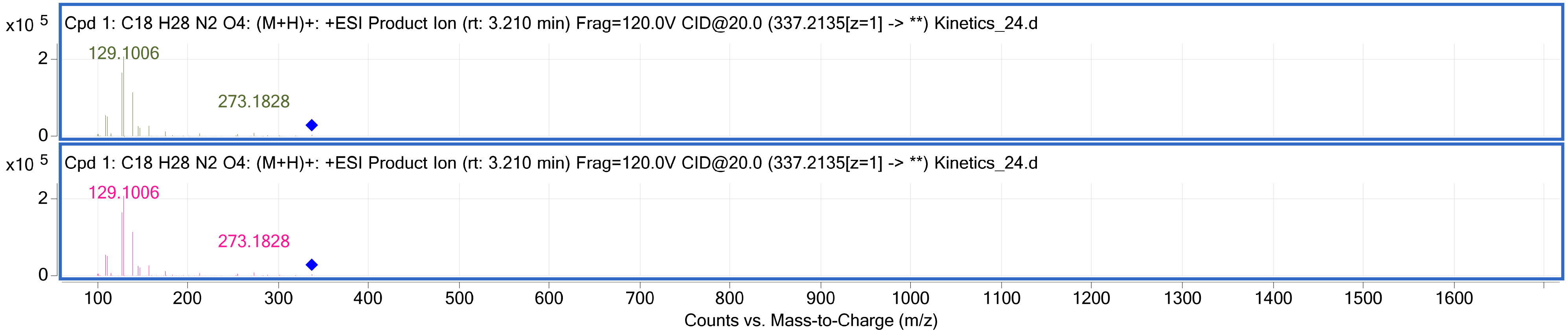

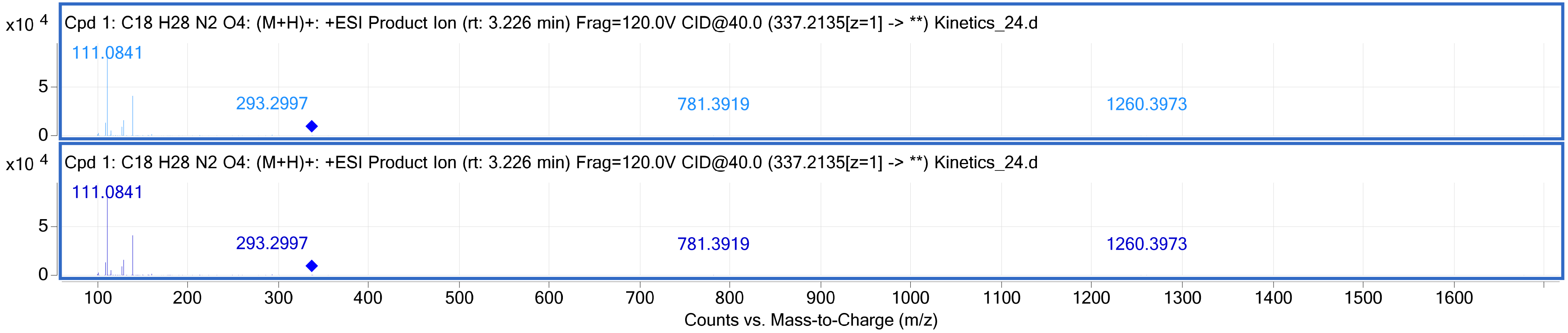

35. **$C_{21}H_{30}O_2$, m/z-337.2134, Cannabidiol**

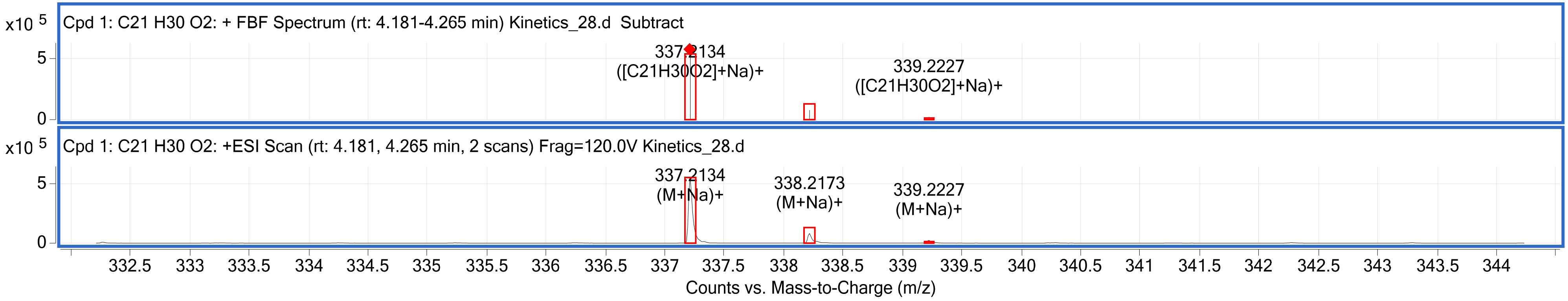

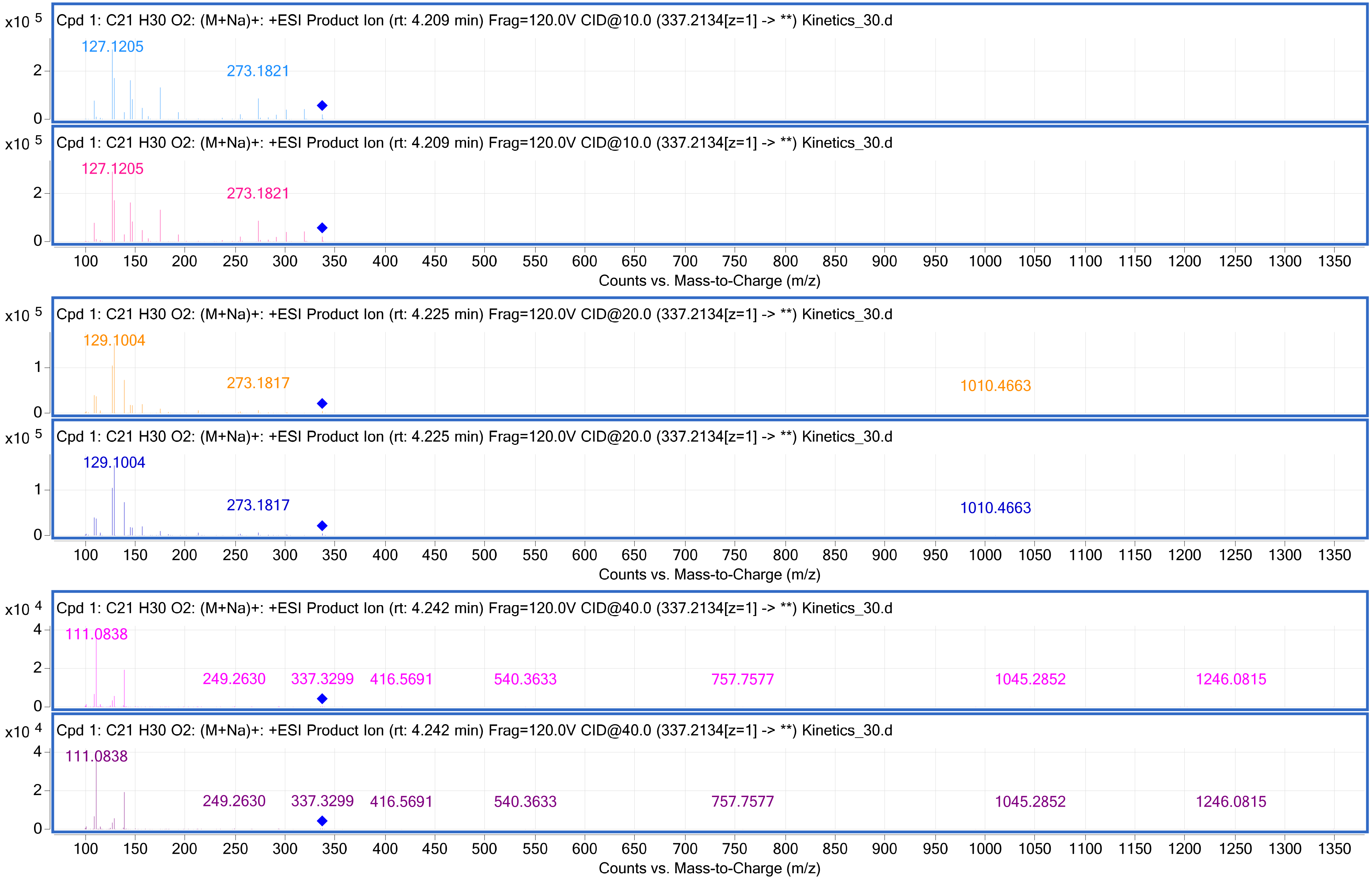

x10 5
Cpd 1: C21 H30 O2: (M+Na)+: +ESI Product Ion (rt: 4.209 min) Frag=120.0V CID@10.0 (337.2134[z=1] -> **) Kinetics_30.d
127.1205
273.1821
x10 5
Cpd 1: C21 H30 O2: (M+Na)+: +ESI Product Ion (rt: 4.209 min) Frag=120.0V CID@10.0 (337.2134[z=1] -> **) Kinetics_30.d
127.1205
273.1821
Counts vs. Mass-to-Charge (m/z)
x10 5
Cpd 1: C21 H30 O2: (M+Na)+: +ESI Product Ion (rt: 4.225 min) Frag=120.0V CID@20.0 (337.2134[z=1] -> **) Kinetics_30.d
129.1004
273.1817
1010.4663
x10 5
Cpd 1: C21 H30 O2: (M+Na)+: +ESI Product Ion (rt: 4.225 min) Frag=120.0V CID@20.0 (337.2134[z=1] -> **) Kinetics_30.d
129.1004
273.1817
1010.4663
Counts vs. Mass-to-Charge (m/z)
x10 4
Cpd 1: C21 H30 O2: (M+Na)+: +ESI Product Ion (rt: 4.242 min) Frag=120.0V CID@40.0 (337.2134[z=1] -> **) Kinetics_30.d
111.0838
249.2630
337.3299
416.5691
540.3633
757.7577
1045.2852
1246.0815
x10 4
Cpd 1: C21 H30 O2: (M+Na)+: +ESI Product Ion (rt: 4.242 min) Frag=120.0V CID@40.0 (337.2134[z=1] -> **) Kinetics_30.d
111.0838
249.2630
337.3299
416.5691
540.3633
757.7577
1045.2852
1246.0815
Counts vs. Mass-to-Charge (m/z)

36. $C_{12}H_{20}O_2$, m/z-219.1353 , Hexenyl-(3z)-hexenoate (3z-)

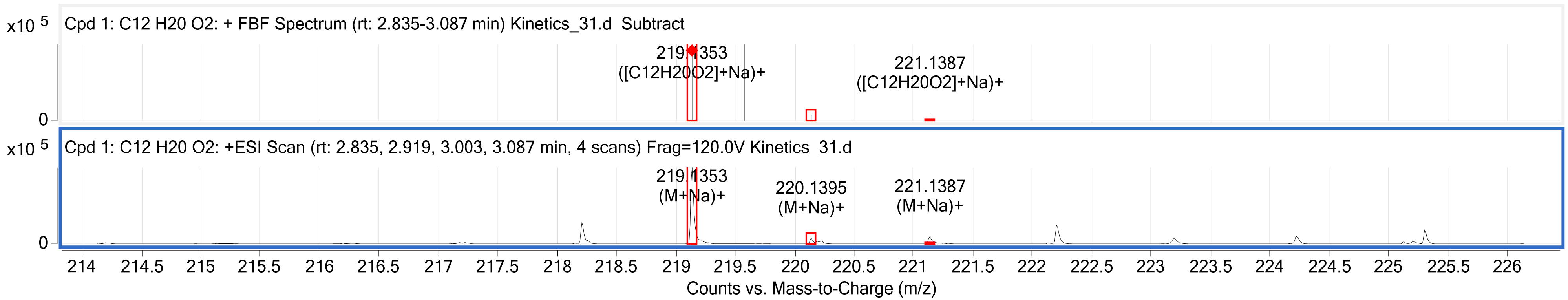

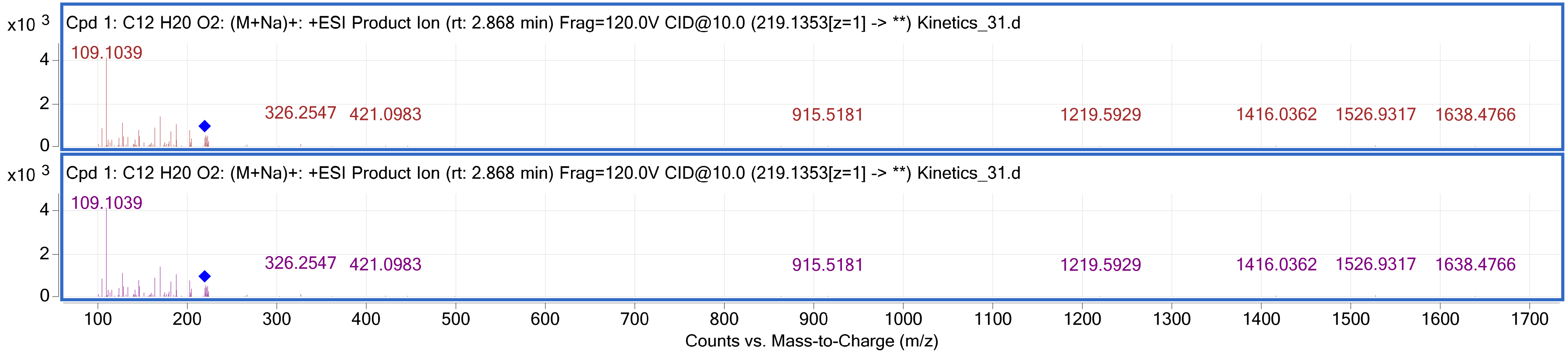

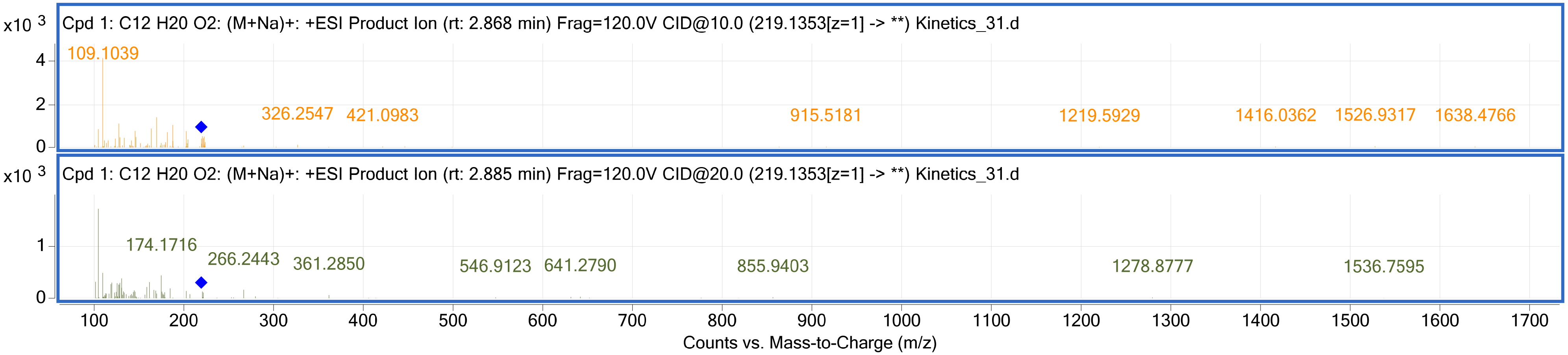
x10 3
Cpd 1: C12 H20 O2: (M+Na)+: +ESI Product Ion (rt: 2.868 min) Frag=120.0V CID@10.0 (219.1353[z=1] -> **) Kinetics_31.d
109.1039
326.2547
421.0983
915.5181
1219.5929
1416.0362
1526.9317
1638.4766
x10 3
Cpd 1: C12 H20 O2: (M+Na)+: +ESI Product Ion (rt: 2.885 min) Frag=120.0V CID@20.0 (219.1353[z=1] -> **) Kinetics_31.d
174.1716
266.2443
361.2850
546.9123
641.2790
855.9403
1278.8777
1536.7595
Counts vs. Mass-to-Charge (m/z)

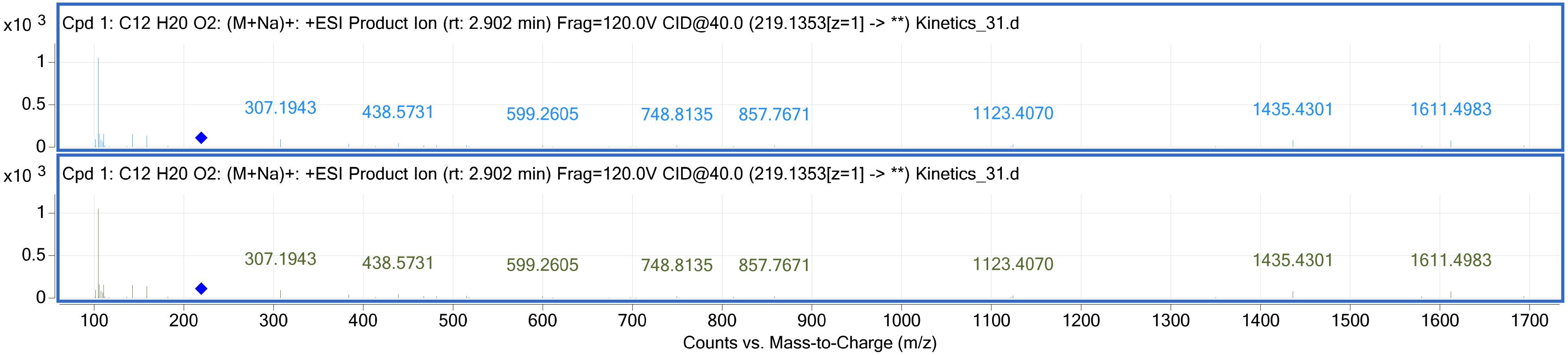
x10 3
Cpd 1: C12 H20 O2: (M+Na)+: +ESI Product Ion (rt: 2.902 min) Frag=120.0V CID@40.0 (219.1353[z=1] -> **) Kinetics_31.d
307.1943
438.5731
599.2605
748.8135
857.7671
1123.4070
1435.4301
1611.4983
x10 3
Cpd 1: C12 H20 O2: (M+Na)+: +ESI Product Ion (rt: 2.902 min) Frag=120.0V CID@40.0 (219.1353[z=1] -> **) Kinetics_31.d
307.1943
438.5731
599.2605
748.8135
857.7671
1123.4070
1435.4301
1611.4983
Counts vs. Mass-to-Charge (m/z)

Figure S3: Confirmation of Embelin from fruit samples. Embelin standard (Sigma-Aldrich) and fruit extract of Embelia was subjected to HPLC and detcted at 288 nm by DAD detector. (A) Peaks eluted from embelin standard and (B) embelia fruit sample are at the same retention time. Concentration of standard embelin was 0.125 ng ml^{-1}.

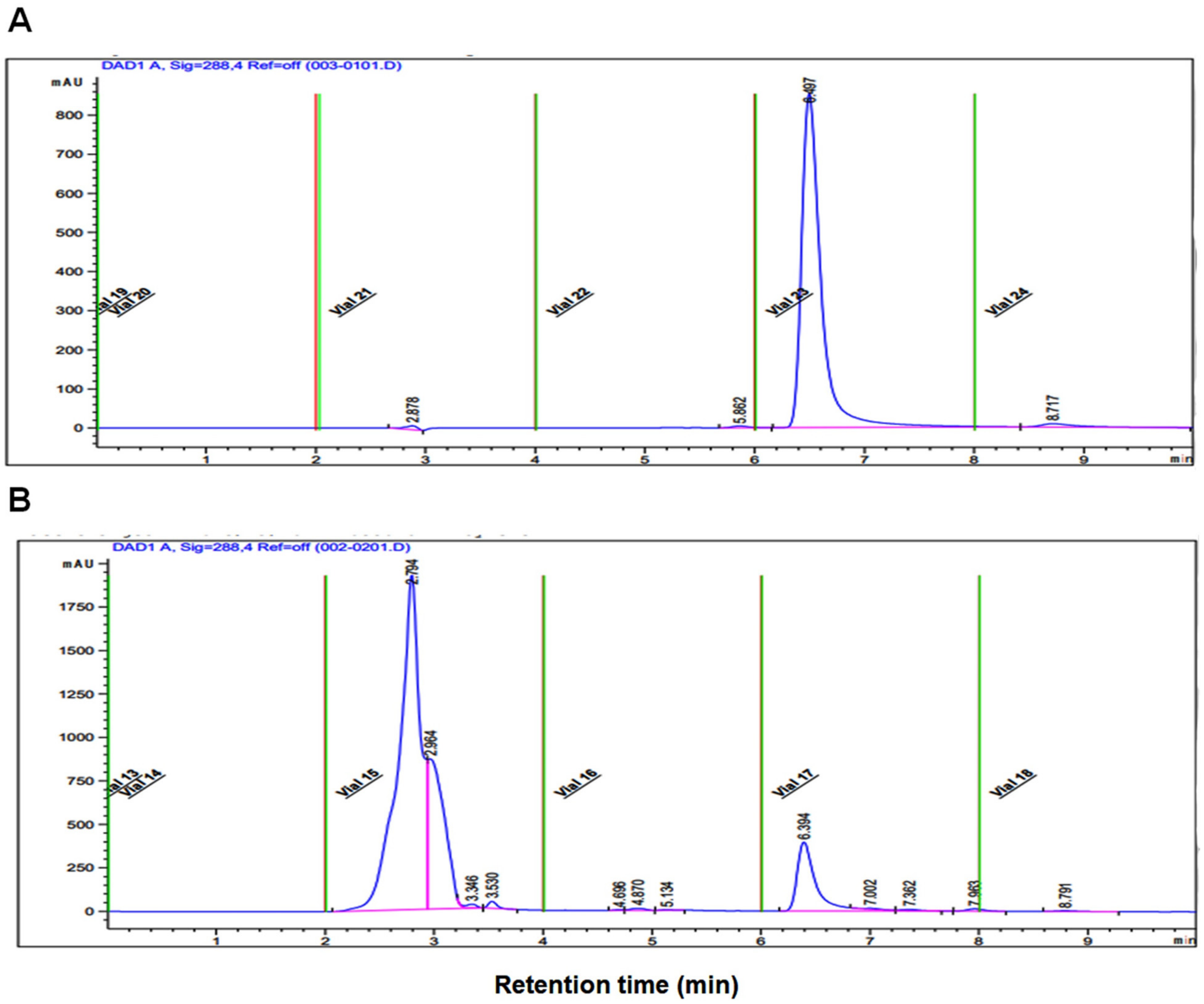

Supplement: Supplementary file 1 [file plants-11-02861-s001.zip › Supplemental figures.pdf]
