# Supplementary material for: Assessing the Impact of Geographical Distribution and Genetic Diversity on Metabolic Profiles of a Medicinal Plant, Embelia ribes Burm. f
Source: Plants (Basel). 2022 Oct 27;11(21):2861. doi: 10.3390/plants11212861 (PMC9653573; doi:10.3390/plants11212861)
Supplement: Supplementary file 1 [file plants-11-02861-s001.zip › Supplemental tables.pdf]

Table S1: NCBI blast results for *matK* sequences. *MatK* sequences obtained from Embelia accessions, E. *tsjeriam-cottam*, and *Measa indica* were analyzed for their similarity with the reported Embelia sequences using the blast function in the NCBI database. Table showed the query cover and percent similarity of obtained *makK* sequences with reported sequences of Embelia.

Sr. No	Sample accessions	Identification	Query cover (%)	Identity (%)
1	Manoli (Maharashtra)	*Embelia ribes*	92	99.87
2	Kodkani (Karnataka)	*Embelia ribes*	92	99.87
3	Nadpal (Karnataka)	*Embelia ribes*	93	99.87
4	Wayanad (Kerala)	*Embelia ribes*	92	99.87
5	Outgroup-1	*Embelia tsjeriam-cottam*	87	99.77
6	Outgroup-2	*Measa indica*	89	98.38

Table S2: Compounds identified tentatively in both positive and negative mode of LC-MS (1-144 in negative mode and 145-149 in positive mode). Total 149 compounds that are present in all five biological replicates of any of the one accession, were tentatively identified based on the formulae from both positive and negative mode of LC-MS. N, Nadpal; K, Kodkani; M, Manoli; W, Wayanad.

Sr No	Name	Formula	Mass	m/z	RT	Score	M	K	N	W
1	3-Heptanone, 5-methyl	$C_8H_{16}O$	128.1206	173.1188	9.298	86.07	-	+	+	+
2	6-Hydroxykaempferol-3-O-b-rutinoside6-O-b-D-glucoside	$C_{10}H_{20}O_2$	172.147	217.1453	11.905	93.14	-	-	-	+
3	Gigantol	$C_{16}H_{18}O_4$	274.1208	319.1189	7.797	80.77	-	-	-	+
4	Multiflorin B	$C_{27}H_{30}O_{15}$	594.1611	593.1536	7.285	85.76	-	+	-	+
5	(-)-Linalool	$C_{10}H_{18}O$	154.1362	199.1346	10.315	96.8	-	+	-	+
6	(+/-)-N,N-Dimethyl menthyl succinamide	$C_{12}H_{24}$	168.187	227.201	11.234	82.82	-	+	+	+
7	(S)-2-Methylbutanoic acid	$C_5H_{10}O_2$	102.0685	161.0824	7.788	84.54	-	+	-	+
8	1,2,3-Trihydroxybenzene	$C_6H_6O_3$	126.0319	125.0248	6.272	94.45	-	+	-	+
9	1,2-Benzoquinone	$C_6H_4O_2$	108.0217	167.0356	9.141	97.34	-	-	+	-
10	1,2-Epoxy-3,4-butanediol 4-methanesulfonate	$C_5H_{10}O_5S$	182.025	227.0226	7.458	83.96	-	+	-	-
11	1,4-Dihydroxyanthraquinone	$C_{14}H_8O_4$	240.0433	299.058	10.898	86.12	-	+	+	-
12	13-Tetradecanolide	$C_{14}H_{26}O_2$	226.1943	271.1925	15.688	91.46	-	+	-	-
13	16,17-Epiestriol	$C_{18}H_{24}O_3$	288.174	347.1881	14.096	76.26	-	+	-	-
14	1-Monopalmitin	$C_{19}H_{38}O_4$	330.2786	375.2774	10.646	83.79	-	+	-	+
15	1-Pentadecene	$C_{15}H_{30}$	210.2355	255.2337	4.679	96.49	-	+	-	+
16	1-Pentanesulfenothioic acid	$C_5H_{12}S_2$	136.0381	195.0517	2.999	81.86	-	+	-	+
17	2,2-Dimethylglutaric acid	$C_7H_{12}O_4$	160.0732	205.0722	7.368	83.03	+	-	-	+
18	2,3-Dihydroxy-3-methylvaleric acid	$C_6H_{12}O_4$	148.0734	147.0666	6.528	86.23	-	+	-	-
19	2,3-Dimethylmaleate	$C_6H_8O_4$	144.0428	143.0358	3.004	72.06	-	-	+	-

Sr No	Name	Formula	Mass	m/z	RT	Score	M	K	N	W
20	25-Hydroxyvitamin D2	$C_{28}H_{44}O_2$	412.3335	411.3262	13.257	79.68	-	+	-	+
21	2-Formylglutarate	$C_6H_8O_5$	160.0379	205.0361	6.365	97.27	+	+	+	+
22	2-Hexyldecanoic acid	$C_{16}H_{32}O_2$	256.2413	255.2341	8.466	93.3	-	+	+	+
23	2-Methyl-3'-hydroxyphenylpropionic acid	$C_{10}H_{12}O_3$	180.0795	225.0777	9.642	94.75	-	+	-	+
24	2-Methylene dodecanoic acid	$C_{13}H_{24}O_2$	212.1779	271.1918	15.260	86.09	+	+	-	+
25	2-Propylglutaric acid	$C_8H_{14}O_4$	174.0897	173.0825	8.634	85.09	-	+	-	+
26	3-(4-Hydroxy-3-methoxyphenyl)propionic acid	$C_{10}H_{12}O_4$	196.074	241.0724	6.695	80.13	-	+	-	-
27	3,4,5-Trihydroxyflavanone	$C_{15}H_{10}O_5$	270.0548	315.0528	10.814	90.25	-	+	-	+
28	3,4-Dihydroxybutyric acid	$C_4H_8O_4$	120.0425	179.0566	3.761	96.11	+	+	-	+
29	3,4-Dimethyl valeric acid	$C_7H_{14}O_2$	130.0998	175.0981	9.969	85.41	-	+	-	-
30	3-[(2S,3R,4S,5S,6R)-4,5-Dihydroxy-6-(hydroxymethyl)-3-[(2S,3R,4S,5R)-3,4,5-trihydroxyoxan-2-yl]oxyoxan-2-yl]oxy-2-(3,4-dihydroxyphenyl)-5-hydroxy-7-methoxychromen-4-one	$C_{27}H_{30}O_{16}$	610.1571	609.1497	7.957	81.38	-	+	-	+
31	3-Amino-3-(4-hydroxyphenyl)propanoate	$C_9H_{11}NO_3$	181.0742	180.0669	4.596	84.77	-	+	-	+
32	3-Furoic acid	$C_5H_4O_3$	112.0166	111.0093	6.024	85.76	-	-	-	+
33	3-Hydroxycapric acid	$C_{10}H_{20}O_3$	188.1422	187.1349	12.831	95.57	-	+	-	+
34	3-Hydroxydodecanoic acid	$C_{12}H_{24}O_3$	216.1734	215.1664	14.18	83.07	-	-	+	+
35	3-Hydroxyvalproic acid	$C_8H_{16}O_3$	160.1098	159.1026	10.221	85.39	+	+	-	+
36	3-Isopropylmalic acid	$C_7H_{12}O_5$	176.0691	175.062	7.71	96.44	-	+	-	+
37	3-Methyl-3-butenyl 3-methylbutanoate	$C_{10}H_{18}O_2$	170.1314	215.1297	10.654	96.42	-	+	-	-

| Sr No | Name | Formula | Mass | m/z | RT | Score | M | K | N | W |

38	3-o-Ethyl-L-ascorbic acid	$C_8H_{12}O_6$	204.0636	263.0783	7.037	75.14	-	+	-	-
39	4-Hydroxycyclohexylacetic acid	$C_8H_{14}O_3$	158.0945	157.0874	11.571	87.9	-	-	-	+
40	4'-Hydroxyfenoprofen	$C_{15}H_{14}O_4$	258.0904	303.0886	8.541	80.27	-	+	-	-
41	4-Hydroxyphenylethanol	$C_8H_{10}O_2$	138.0685	183.0667	10.393	86.13	-	+	-	-
42	4-Methyl-3-oxoadipate-enol-lactone	$C_7H_8O_4$	156.0433	215.0572	7.206	95.20	-	-	-	+
43	4-Methyl-octanoic acid	$C_9H_{18}O_2$	158.1311	217.1451	11.482	90.79	-	+	-	+
44	4-methylpentyl 3-methylbutanoate	$C_{11}H_{22}O_2$	186.1624	185.1546	13.424	86.72	-	+	-	+
45	5-Carboxyvanillic acid	$C_9H_8O_6$	212.033	211.0256	3.755	93.39	-	+	-	+
46	5-O-Methylembelin	$C_{18}H_{28}O_4$	308.1997	307.1924	13.928	80.26	-	+	-	+
47	8'-Hydroxyabscisate	$C_{15}H_{20}O_5$	280.132	279.1247	9.129	94.28	-	-	-	+
48	9,12,15-octadecatrienoic acid,2-phenyl-1,3-dioxan5-yl ester	$C_{28}H_{40}O_4$	440.2946	439.2879	15.515	86.76	-	+	+	-
49	Aconitic acid	$C_6H_6O_6$	174.0172	173.0099	6.281	97.02	-	+	-	+
50	Alfafuran	$C_{14}H_{10}O_5$	258.0538	303.0518	9.048	82.33	-	+	-	+
51	Alpha-Ionone	$C_{13}H_{20}O$	192.1517	251.1654	14.686	81.60	+	+	+	+
52	Aminofurantoin	$C_8H_8N_4O_3$	208.0599	267.0736	3.00	92.68	-	+	-	-
53	arbutin	$C_{12}H_{16}O_7$	272.0904	317.0888	4.853	93.31	+	+	-	+
54	Arjunolic acid	$C_{30}H_{48}O_5$	488.3526	547.3671	15.351	84.63	-	+	-	+
55	Artemether	$C_{16}H_{26}O_5$	298.1794	297.1719	15.431	91.87	-	+	-	+
56	Artesunate	$C_{19}H_{28}O_8$	384.1781	383.1701	13.34	75.20	-	+	-	-
57	Aucubin	$C_{15}H_{22}O_9$	346.1279	345.1208	7.281	91.01	-	+	-	+
58	Benzaldehyde	C_7H_6O	106.0423	151.0405	10.066	98.41	-	-	-	+
59	Benzofuran	C_8H_6O	118.0425	163.0407	8.881	97.45	-	+	-	-
60	Benzoic acid	C_7H_6O2	122.0373	121.03	9.138	96.01	-	+	-	+
61	Benzoquinoneacetic acid	$C_8H_6O_4$	166.027	211.0252	5.184	85.00	-	+	-	-
62	Benzyl 3-methylbutanoate	$C_{12}H_{16}O_2$	192.1158	251.1295	10.394	81.87	-	+	-	+
63	Beta dehydro-agarofuran	$C_{14}H_{22}O$	206.1674	265.1812	11.664	85.51	-	+	-	+

Sr No	Name	Formula	Mass	m/z	RT	Score	M	K	N	W
64	Bolegrevilol	$C_{13}H_{24}O_2$	212.1786	257.1768	14.595	90.82	-	-	-	+

65	Caffeine	$C_8H_{10}N_4O_2$	194.0799	239.0784	4.176	93.50	-	+	+	-
66	Capsidiol	$C_{15}H_{24}O_2$	236.1788	281.1771	11.065	80.15	-	+	-	+
67	Casbene	$C_{20}H_{32}$	272.251	317.2493	14.598	84.07	-	+	-	-
68	Cis-Gondoic acid	$C_{20}H_{38}O_2$	310.2875	309.2802	11.906	80.39	-	+	-	-
69	Citric acid	$C_6H_8O_7$	192.0278	191.0205	4.256	83.59	+	+	-	+
70	Citronellyl formate	$C_{11}H_{20}O_2$	184.1469	229.145	13.592	93.74	-	+	-	+
71	Coenzyme Q4	$C_{29}H_{42}O_4$	454.3102	453.3032	10.729	85.25	-	-	-	+
72	Coumaric acid	$C_9H_8O_3$	164.048	163.0407	8.881	97.45	-	+	-	-
73	Crystambine	$C_{35}H_{52}O_8$	600.3689	599.3624	14.352	76.01	-	+	-	-
74	Cynaroside	$C_{21}H_{20}O_{11}$	448.1031	447.0957	8.63	87.12	-	+	-	+
75	Bolegrevilol	$C_{28}H_{40}O_4$	440.2927	439.2854	15.53	80.54	+	+	-	+
76	D-Erythroascorbic acid 1'-a-D-glucoside	$C_{11}H_{16}O_{10}$	308.0761	353.0743	4.176	91.50	-	+	-	-
77	D-Glucoside	$C_7H_{14}O_6$	194.0798	239.0779	4.012	92.42	-	+	-	-
78	Di 2- propenyl disulphide	$C_6H_{10}S_2$	146.0221	191.0201	4.265	80.73	-	+	+	+
79	Diacetyl	$C_4H_6O_2$	86.0367	145.051	6.029	85.87	-	+	-	-
80	Dihydro-3-coumaric acid	$C_9H_{10}O_3$	166.0638	225.0775	9.637	95.72	-	+	+	-
81	Dihydroartemisinin	$C_{15}H_{24}O_5$	284.1622	283.156	8.206	74.76	-	+	-	+
82	Dihydrojasmonic Acid, Methyl Ester	$C_{13}H_{22}O_3$	226.1569	285.1706	14.68	83.57	-	+	-	+
83	Dihydroxyacetone	$C_3H_6O_3$	90.0321	135.0305	3.004	97.57	-	+	-	+
84	Diplosalsalate	$C_{16}H_{12}O_6$	300.0646	299.0573	10.981	93.57	-	+	-	+
85	DL-Î±-Lipoic Acid	$C_8H_{14}O_2S_2$	206.0436	205.0361	6.365	82.15	-	+	-	+
86	D-Mannonate	$C_6H_{12}O_7$	196.0587	195.0514	2.916	99.18	+	-	-	+
87	Dodecanedioic acid	$C_{10}H_{18}O_4$	202.1212	201.1139	9.974	97.41	+	+	-	+
88	Elemicin	$C_{12}H_{16}O_3$	208.1102	207.1031	12.752	86.82	-	-	+	-
89	Embelin	$C_{17}H_{26}O_4$	294.1835	293.1776	14.434	70.13	+	+	+	+
90	Embeliol	$C_{17}H_{28}O_4$	296.2002	341.1986	13.078	74.75	-	-	-	+
91	Embeliquinone	$C_{22}H_{36}O_4$	364.2626	363.2556	14.684	90.73	-	+	-	-

**Sr No**	**Name**	**Formula**	**Mass**	**m/z**	**RT**	**Score**	**M**	**K**	**N**	**W**
92	Ethyl 1-methylpropyl disulfide	$C_6H_{14}S_2$	150.0538	195.0518	2.92	81.00	-	+	-	+

93	Ethyl dodecanoate	$C_{14}H_{28}O_2$	228.2085	227.2011	14.598	85.81	-	+	-	+
94	E-trimenal	$C_{13}H_{22}O$	194.1677	253.1816	13.166	84.84	-	+	-	-
95	Forskolin	$C_{22}H_{34}O_7$	410.2312	469.2441	15.347	80.29	-	+	-	-
96	Fumaric acid	$C_4H_4O_4$	116.0113	115.0043	3.172	85.20	-	+	-	+
97	Furanone	$C_4H_4O_2$	84.0215	143.0353	7.122	85.97	-	+	-	+
98	Furodysin	$C_{15}H_{20}O$	216.1522	275.166	10.645	84.28	-	+	-	-
99	Galactonic acid	$C_6H_{12}O_7$	196.0587	195.0514	2.916	99.18	-	+	-	+
100	Gamolenic acid	$C_{18}H_{30}O_2$	278.2251	277.2185	11.905	80.77	-	+	-	+
101	Geinstin	$C_{18}H_{26}O_4$	306.1817	351.1822	14.516	81.39	-	+	-	+
102	Geranyl-hydroxybenzoate	$C_{17}H_{22}O_3$	274.157	319.1539	11.242	75.72	-	+	-	-
103	Glucoheptonic acid	$C_7H_{14}O_8$	226.0694	285.0838	3.084	92.46	-	+	-	+
104	Heneicosanoic acid	$C_{21}H_{42}O_2$	326.3192	371.3174	14.771	80.68	-	-	-	+
105	Hexadecanedioic acid	$C_{16}H_{30}O_4$	286.2151	285.2079	11.571	94.66	-	+	-	+
106	Î´-Hexalactone	$C_6H_{10}O_2$	114.0685	113.0613	8.298	85.61	-	+	-	-
107	Î±-Phenylcyclohexylglycolic acid	$C_{14}H_{18}O_3$	234.1263	279.1245	9.133	84.32	-	+	-	-
108	Isocaproic acid	$C_6H_{12}O_2$	116.084	161.0822	7.374	86.81	+	+	-	+
109	Isoquercitrin	$C_{21}H_{20}O_{12}$	464.098	463.0907	8.793	87.18	-	+	-	-
110	Isorhamnetin	$C_{16}H_{12}O_7$	316.0597	315.0524	10.726	78.44	-	-	-	+
111	Kaempferol 3-O-xyloside	$C_{20}H_{18}O_{10}$	418.0925	463.0907	8.213	87.18	-	+	-	+
112	Lactapiperanol C	$C_{16}H_{26}O_4$	282.1843	327.1828	13.592	86.18	-	+	-	+
114	Lespedezaflavanone F	$C_{27}H_{32}O_5$	436.225	435.2179	9.476	78.25	-	-	+	-
115	Lipoic acid, reduced	$C_8H_{16}O_2S_2$	208.0595	207.0516	3.756	84.86	-	+	-	+
116	Lumichrome	$C_{12}H_{10}N_4O_2$	242.0808	241.0734	7.788	84.68	+	+	-	+
117	Malic acid	$C_4H_6O_5$	134.0221	133.0148	4.097	98.07	-	+	-	+
118	N-(3-Carboxylpropyl)-5-amino-2-hydroxy-3-tridecyl-1,4-benzo-quinone	$C_{23}H_{38}NO_5$	408.2751	407.2676	10.148	80.83	-	-	+	-
119	Naringenin	$C_{15}H_{12}O_5$	272.0696	271.0622	10.906	93.52	-	+	-	-
120	N-propyl sec-butyl disulfide	$C_7H_{14}S_2$	162.0536	221.0676	6.785	85.79	+	+	+	+
**Sr No**	**Name**	**Formula**	**Mass**	**m/z**	**RT**	**Score**	**M**	**K**	**N**	**W**
121	Oxalic acid	$C_2H_2O_4$	89.9957	149.0098	3.084	93.78	-	+	-	-

122	Oxaloacetate	$C_4H_4O_5$	132.0065	191.0203	4.256	98.09	-	+	-	+
123	Pentan-3-one	$C_5H_{10}O$	86.0733	131.0713	9.814	85.65	-	-	+	+
124	Phenylglucoside	$C_{12}H_{16}O_6$	256.0959	255.0887	9.306	80.09	-	+	-	-
125	Piceatannol	$C_{14}H_{12}O_4$	244.0744	243.0674	8.370	71.91	-	+	-	-
126	p-Methylolphenol	$C_7H_8O_2$	124.0528	183.0667	10.393	86.50	-	+	-	-
127	Procurcumenol	$C_{15}H_{22}O_2$	234.1627	293.1776	15.534	81.44	-	+	+	+
128	Propiolic acid	$C_3H_2O_2$	70.0052	115.0035	15.445	86.19	-	+	-	-
129	Purifolin	$C_{24}H_{24}O_{13}$	520.1202	519.113	4.938	73.89	-	-	-	+
130	Quercetin	$C_{15}H_{10}O_7$	302.0441	301.0368	10.141	92.90	-	+	-	-
131	Quinol glucuronide	$C_{12}H1_4O_8$	286.07	331.0682	5.516	91.73	-	+	-	-
132	Rapanone	$C_{19}H_{30}O_4$	322.2153	321.2087	13.171	85.73	-	+	-	+
133	Salidroside	$C_{14}H_{20}O_7$	300.1223	345.1207	7.284	88.14	-	-	-	+
134	Stearic acid	$C_{18}H_{36}O_2$	284.2723	283.2652	11.911	93.75	+	+	+	+
135	Sundiversifolide	$C_{14}H_{22}O_3$	238.1571	297.1709	11.319	84.01	-	+	-	+
136	Tetradecene	$C_{14}H_{28}$	196.22	255.2339	0.572	96.94	+	+	-	+
137	Tetrahydrofuran	C_4H_8O	72.0577	117.0559	6.529	86.55	-	+	-	+
138	Theobromine	$C_7H_8N_4O_2$	180.0644	179.0571	3.004	96.13	+	+	+	+
139	Tricin	$C_{17}H_{14}O_7$	330.0755	329.0679	10.814	85.51	-	+	-	-
140	Tridecanoic acid	$C_{13}H_{26}O_2$	214.1943	213.187	15.272	95.31	+	+	-	+
141	Undecan-2-one	$C_{11}H_{22}O$	170.1678	215.1661	11.299	94.73	-	+	-	+
142	Uracil	$C_4H_4N_2O_2$	112.0275	111.0202	15.1	87.52	-	+	-	+
143	Vanillic acid	$C_8H_8O_4$	168.0427	167.0355	9.133	85.42	-	+	-	+
144	Xanthoxic acid	$C_{15}H_{22}O_4$	266.1535	265.1461	12.835	85.79	-	+	-	-
145	Hexenyl(3Z)-Hexenoate,(3Z)	$C_{12}H_{20}O_2$	196.1456	219.1352	2.833	70.31	-	+	-	+
146	10-Oxogeranial	$C_{10}H_{14}O_2$	166.10	167.1071	2.583	78.90	-	+	+	+
147	Acebutolol	$C_{18}H_{28}N_2O_4$	336.2061	337.2131	3.160	84.47	-	+	-	+
148	Cannabidiol	$C_{21}H_{30}O_2$	314.2244	337.2134	4.181	84.58	-	+	+	-
149	Trimipramine	$C_{20}H_{26}N_2$	294.2095	295.2168	11.072	86.08	-	+	+	-

Table S3: Contribution of differentially regulated compounds in groupings of the accessions. Results of pairwise analysis of similarity percentage (SIMPER test) with Bray-Curtis dissimilarity index showed 15 major compounds (50% cut-off) contributed in accession groupings.

Compounds	Contribution %	Cumulative contribution%
Acebutolol	3.958	3.958
Cannabidiol	3.958	7.916
3-Isopropylmalic acid	3.534	11.45
Decanedioic acid	3.534	14.98
delta-Tridecalactone	3.534	18.52
Citric acid	3.534	22.05
tetradecene	3.534	25.59
procurcumenol	3.453	29.04
hexenyl-(3z)-hexenoate (3z-)	3.453	32.49
di-2-Propenyl disulfide	3.453	35.94
3-Furoic acid	3.003	38.95
Malic acid	3.003	41.95
Oxaloacetate	3.003	44.95
2-Formylglutarate	3.003	47.95
3-Amino-3-(4-hydroxyphenyl) propanoate	3.003	50.96

Table S4: Reported functions of the identified compounds from Embelia accessions.

Sr no	Compound name	Specific category	Function	Reference
1	1,2-benzoquinone	O-benzoquinones	Fungiside, antimicrobial	M., H. A. Ahmed, and H.A. M. Mossabl, 2014.
2	1-pentanesulfenothoic acid	Hydropersulfides	Plant and bacterial metabolite (Function not known)	Pubchem
3	2-formylglutarate	Branched fatty acids	Plant metabolite (Function not known)	Pubchem
4	3,4,5-trihydroxyflavanone	Flavanones	Antidepressent, antimutagenic an antioxidant	1) Barboza, R. S., et al. 2018 2) Grigalius,I. and Vilma P. 2017
5	3,4-dihydroxybutyric acid	Hydroxy fatty acids	Satiety or the feeling of fullness and suppresses food intake	Kumar, P., et al. 2005
6	3-amino-3-(4-hydroxyphenyl) propanoate	β-amino acids and derivatives	Anticancer	Chen, Y., et al. 2009
7	3-furoic acid	Furans	Plant metabolite (Function not known)	Pubchem
8	3-isopropylmalic acid	Hydroxy fatty acids	Plant and bacterial metabolite (Function not known)	Pubchem
9	Aconitic acid	Tricarboxylic acid	Anticancers	Kumar, V. and Nanthini R.2018
10	Benzoic acid	Benzenoids	Fungistatic	Gould, A.G., and Edna K. C.1930
11	Bolegrevilol	Diterpenoid	Antioxident	Polterait, O.,1997
12	Citric acid	Tricarboxylic acid	Anticoagulant	Chen, H. et al, 2021
13	Coumaric acid	Hydroxycinnamic acids	Antihemorrhagic activity	De Sousa F. et al. 2021
14	Decanedioic acid	Medium-chain dicarboxylic acids	Antagonists of the oestrogen receptor alpha (er-alpha)	Pubchem
15	Delta-tridecalactone	Delta-lactone	Anticancers	Pubchem
16	Di-2-propenyl disulfide	Diallyl disulfide	Antineoplastic agent, an antifungal agent	Pyun, M-S., and S. Shin, 2006
17	Dl-alpha-lipoic acid	Dithiol short chain fatty acid	Anti-oxidant	Shay, K.P.et al. 2009
18	Embelin	P-benzoquinones	Antimicrobial, antihelminthic, antipyretic, antioxidant,	Radhakrishnan, N., et al. 2011

Sr no	Compound name	Specific category	Function	Reference
19	Ethyl-1-methylpropyl disulfide	Dialkyldisulfides	Plant metabolite (Function not known)	Farhadi, F., et al. 2020
20	Galactonic acid	Sugar acids	Plant and animal metabolite (Function not known)	Wagner, C., Michael S. and Joachim K. 2003
21	Hexadecanedioic acid	Long-chain dicarboxylic acid acids	Anticancer	Bharath, B., et al. 2021
22	Isoquercitrin	Flavonol glycosides	osteogenesis regulator, an antioxidant,<br>a histamine antagonist, an antipruritic drug	Pubchem
23	Malic acid	2-hydroxydicarboxylic acid	Xerostomia, depression, and hypertension.	Gómez-Moreno, G.2013
24	Naringenin	Flavanones	Modulates immune system activity, antioxidant, anti-atherogenic and anti-inflammatory	Pubchem
25	N-propyl sec-butyl disulfide	Dialkyldisulfides	Essential oil, antimicrobial	Daneshkazemi, A., et al. 2019
26	Oxaloacetate	Dicarboxylic acid	Antagonists of the androgen receptor (ar) signalling pathway using	Pubchem
27	Piceatannol	Stilbenes	Antineoplastic agent, a plant metabolite, a hypoglycaemic agent, an apoptosis inducer	Banik, K., et al. 2020
28	Procurcumenol	Guaiane sesquiterpenes	Anti-inflammatory	Nishidono, Y., et al. 2020
29	Quercetin	Flavonols	Combined with chemotherapeutic drugs, in vitro, produces anti-inflammatory and anti-allergy effect	Chirumbolo, S2010
30	Rapanone	P-benzoquinones	Inhibits mitochondrial respiration and induces hepg2 cell death	Pubchem
31	Tetradecene	Unsaturated aliphatic hydrocarbons	Antagonists of the estrogen receptor alpha	Pubchem

Sr no	Compound name	Specific category	Function	Reference
32	Undecan-2-one	Fatty acyl	Anticancer	Tanoh, E. A., et al2018
33	Vanillic acid	Hydroxybenzoic acids	Inhibits inflammatory pain by inhibiting neutrophil recruitment, oxidative stress, cytokine production, and NFKB activation	Pubchem
34	Acebutolol	Alkyl-phenylketones	Has stabilizing and quinidine-like effects on cardiac rhythm as well as weak inherent sympathomimetic action.	Pubchem
35	Cannabidiol	Cannabinoids	Anti-proliferative, anti-angiogenic and pro-apoptotic activity through various mechanisms,	Sainz-Cort, A., Claudia M.-S., and Enric E. 2020
36	Hexenyl-(3z)-hexenoate (3z-)	Wax monoesters	Xerostomia, depression, and hypertension.	Al-S.and Ali E. 2017

Table S5: List of ISSR primers with their nucleotide sequences and annealing temperatures used for amplification. A total nine polymorphic ISSR primers were used for genetic diversity analysis.

Sr No	Primer	Sequence	T_m (˚C)
1	ISSR 809	$(AG)_8G$	54
2	ISSR 810	$(GA)_8T$	54
3	ISSR 811	$(GA)_8C$	54
4	ISSR 816	$(CA)_8T$	53
5	ISSR 821	$(GT)_8T$	50
6	ISSR 849	$(GT)_8YA$	50
7	ISSR 853	$(TC)_8RT$	50
8	ISSR 857	$(AC)_8YG$	60
9	ISSR 881	$(G)_{12}(T)_3$	50

Table S6: Analysis of genetic diversity in Embelia accession by ISSR primers.

Sr. No.	ISSR primer	Monomorphic bands	Polymorphic bands	Total bands	% polymorphism	% polymorphic bands (PPB)
1	ISSR 809	00	12	12	100	12.76
2	ISSR 810	00	09	09	100	9.57
3	ISSR 811	00	08	08	100	8.51
4	ISSR 816	03	06	09	66.6	6.38
5	ISSR 821	00	10	10	100	10.63
6	ISSR 849	00	11	11	100	11.70
7	ISSR 853	00	10	10	100	10.63
8	ISSR 857	00	13	13	100	13.82
9	ISSR 881	01	11	12	91.6	11.70
